# The Role of Oxidative Stress in Nervous System Aging

**DOI:** 10.1371/journal.pone.0068011

**Published:** 2013-07-02

**Authors:** Catrina Sims-Robinson, Junguk Hur, John M. Hayes, Jacqueline R. Dauch, Peter J. Keller, Susan V. Brooks, Eva L. Feldman

**Affiliations:** 1 Department of Neurology, University of Michigan, Ann Arbor, Michigan, United States of America; 2 Department of Molecular and Integrative Physiology, University of Michigan, Ann Arbor, Michigan, United States of America; University of Florida, United States of America

## Abstract

While oxidative stress is implicated in aging, the impact of oxidative stress on aging in the peripheral nervous system is not well understood. To determine a potential mechanism for age-related deficits in the peripheral nervous system, we examined both functional and morphological changes and utilized microarray technology to compare normal aging in wild-type mice to effects in copper/zinc superoxide dismutase-deficient (*Sod1^−/−^*) mice, a mouse model of increased oxidative stress. *Sod1^−/−^* mice exhibit a peripheral neuropathy phenotype with normal sensory nerve function and deficits in motor nerve function. Our data indicate that a decrease in the synthesis of cholesterol, which is vital to myelin formation, correlates with the structural deficits in axons, myelin, and the cell body of motor neurons in the *Sod1^+/+^* mice at 30 months and the *Sod1^−/−^* mice at 20 months compared with mice at 2 months. Collectively, we have demonstrated that the functional and morphological changes within the peripheral nervous system in our model of increased oxidative stress are manifested earlier and resemble the deficits observed during normal aging.

## Introduction

Neuromuscular system function declines with age and manifests as dramatic decreases in muscle strength and size, often referred to as sarcopenia [1]. Skeletal muscle atrophy and weakness lead to the loss of functional mobility and independence for many older adults [2]. Age-related changes in the central nervous system are well documented and include neuronal loss, demyelination, and deficits in cognitive function; however, little has been reported concerning age-related changes in the peripheral nervous system beyond a decline in nerve conduction velocities (NCVs) [3]. A clear understanding of the mechanisms underlying age-related changes in the peripheral nervous system is necessary to fully understand and prevent the decline in neuromuscular function that often accompanies aging.

The oxidative stress or free radical theory of aging, proposed by Denham Harman, suggests that free radicals cause oxidative damage to proteins, DNA, and lipids, and that this damage accumulates over time [4], [5], [6]. Oxidative stress is the result of an imbalance between pro-oxidants and antioxidants [5]. To date, both invertebrate and vertebrate models have been generated in which one or more antioxidants are either ablated or over-expressed; however, the role of oxidative stress in aging vertebrates, including rat, mouse, and human, remains unclear, likely due to the complexity of the aging process [7].

All cells contain multiple enzymes that target and neutralize free radicals. Superoxide dismutase (SOD) partners with another antioxidant enzyme, catalase, to defend against oxidative damage by converting the free radical pro-oxidant superoxide anion into molecular oxygen and hydrogen peroxide. There are three mammalian forms of SOD: cytoplasmic copper/zinc or SOD1, mitochondrial manganese or SOD2, and extracellular or SOD3. SOD1-deficient (*Sod1* ^−/−^) mice appear normal at birth, but over time exhibit increased levels of oxidative stress in muscle and plasma, display a chronic peripheral neuropathy, accelerated age-associated hind limb muscle mass loss, neuromuscular junction degeneration, muscle weakness, and a 30% reduction in lifespan [8], [9], [10], [11]. Furthermore, we previously reported that denervation in the muscles of the *Sod1^−/−^* mice resulted in fiber loss and muscle atrophy [12]. Mice lacking SOD2 (*Sod2^−/−^*) die shortly after birth from cardiomyopathy and neurodegeneration [13]; whereas *Sod2^+/−^* mice are viable until adulthood with no change in lifespan and exhibit elevated markers of oxidative damage and increased sensitivity to oxidative stress [14]. Mice lacking SOD3 (*Sod3^−/−^*) are healthy and exhibit a normal lifespan [15]. Thus, given the severity of the muscle phenotype and resemblance to human aging, the *Sod1* ^−/−^ mouse model is more informative than models with other SOD mutations for studying the effects of oxidative stress on aging in the peripheral nervous system. The aim of this study is to better understand the contribution of oxidative stress in peripheral nervous system aging. Here, we accomplish this aim by correlating functional and morphological changes in sensory and motor neurons with microarray and bioinformatics analyses of nerve during normal aging in *Sod1* ^+/+^ mice and in *Sod1* ^−/−^ mice, a model with increased oxidative stress.

## Materials and Methods

All protocols and procedures were approved by the University of Michigan Committee on the Use and Care of Animals (UCUCA; approval number PRO00003694), and are in compliance with the University guidelines, State and Federal regulations, and the standards of the “Guide for the Care and Use of Laboratory Animals.” The University’s Animal Welfare Assurance Number on file with the NIH Office of Laboratory Animal Welfare (OLAW) is A3114-01, and the University is accredited by the Association for the Assessment and Accreditation of Laboratory Animal Care International (AAALAC, Intl.). The mice for this study were generated and genotyped as previously described [9], [16]. The *Sod1* ^+/+^ and *Sod1* ^−/−^ mice used in the current study were bred and maintained by the Transgenic Animal Core (University of Texas Health Science Center, San Antonio, Texas) and shipped to the University of Michigan at 2, 8, 20, and 30 mo (*Sod1* ^+/+^) or 2, 8, and 20 mo (*Sod1* ^−/−^). At the end of the study, the mice were euthanized with an overdose of Avertin followed by bilateral pneumothorax. The sciatic nerve (SCN), spinal cord, dorsal root ganglia (DRG), and/or ventral root were processed as described below.

### Thermal Thresholds

Tail flick analgesia was measured according to previously published protocols [17]. Briefly, for the tail flick assay, mice were placed in an acrylic holder atop a tail flick analgesia meter (IITC Life Science, Woodland Hills, CA) with the tail in contact with an adjustable red light emitter (range 60°–170°C). The time from activation of the beam to the animal response is detected and electronically recorded.

### Nerve Conduction Studies

Measures of NCV were performed at 2, 8, 20, and 30 mo of age per our published protocols [17]. Mice were anesthetized with Avertin (200 mg/kg) and body temperature was maintained at 34°C using a warming lamp and dermal temperature probe. The platinum needle electrodes (ViaSys, Madison, WI) were cleaned between measurements using 70% alcohol. Tail distal motor latency (TDML) is an orthodromic measurement determined by stimulating the proximal 30 mm segment of the tail. Latency was measured from the initial onset of the compound muscle action potential. The sural NCV, a measure of sensory nerve function, was determined by recording in the dorsum of the foot and stimulating antidromically with supramaximal stimulation at the ankle and recorded at the dorsum of the foot. NCV was calculated by dividing the distance by the take-off latency of the sensory nerve action potential. The sciatic-tibial motor NCV (SMNCV) was determined by recording in the dorsum of the foot and orthodromically stimulating with supramaximal stimulation first at the ankle then at the sciatic notch. Latencies were measured from the initial onset of the compound muscle action potential. Final NCV was calculated by dividing the difference of the ankle from the notch distance by the difference in the ankle and notch latencies.

### Tissue Processing

For microarray analysis, one SCN was stored in RNAlater (Ambion, Inc., An Applied Biosystems Buisness, Austin, TX) at −80°C until use for RNA isolation and gene expression analysis. For western immunoblotting, the tissue was snap frozen in liquid nitrogen and stored at −80°C until use for western immunoblotting. For lipofuscin analysis, light or transmission electron microscopy, animals were intracardially perfused with 15 ml of phosphate buffered saline (PBS 0.1 M, pH 7.2) and 30 ml 2% paraformaldehyde prior to dissection. For light and transmission electron microscopy, L_5_ spinal cord and ventral root were dissected and post-fixed overnight in 4% paraformaldehyde/2.5% glutaraldehyde. Tissue was processed by the Microscopy Imaging Laboratory (MIL; University of Michigan, Ann Arbor, MI).

### Lipofuscin Analysis

For lipofuscin analysis, sections were heated on a slide warmer for 10 min, hydrated in PBS for 5 min, and coverslipped with Prolong anti-fade mounting medium containing DAPI. The images were captured using a Spot-RT camera (Diagnostic Instruments Inc., Sterling Heights, MI) attached to a Nikon Microphot-FXA microscope. The resulting images of lipofuscin were analyzed for intensity using MetaMorph software (Universal Imaging Corp., West Chester, PA).

### Western Immunoblotting

Western immunoblotting was performed as previously described [18]. SCN, lumbar spinal cord, and DRG were homogenized in tissue protein extraction reagent (Pierce, Rockford, IL) containing a protease inhibitor cocktail (Calbiochem, San Diego, CA). The lysates were separated by SDS-PAGE and transferred to a nitrocellulose membrane. Tris-buffered saline with Tween-20 supplemented with 5% non-fat dry milk was used to decrease non-specific binding and dilute the following primary antibodies: nitrotyrosine (NT), malondialdehyde (MDA) (both from Abcam, Cambridge, MA), cleaved caspase-3 (Millipore, Billerica, MA), 7-dehydrocholesterol reductase (DHCR7; Abcam), 24-dehydrocholesterol reductase (DHCR24, Cell Signaling, Danvers, MA), and mevalonate diphospho decarboxylase (Santa Cruz, Dallas, Texas). The signal was visualized using LumiGLO enhanced chemiluminescence reagent (Cell Signaling Technology, Danvers, MA). Images were captured using the Chemidoc XRS system and analyzed by Quantity One software (Bio-Rad Laboratory, Hercules, CA).

### RNA Preparation

Total RNA was isolated from one SCN (the other SCN was used for western immunoblotting as described above) using the RNeasy Mini Kit (QIAGEN, Inc., Valencia, CA), including an on-column deoxyribonuclease digestion, following the manufacturer’s protocol. RNA quality and quantity were assessed by microfluid electrophoresis using an RNA 6000 Pico LabChip on a 2100 Bioanalyzer (Agilent Technologies, Inc., Santa Clara, CA). Samples with a minimum RNA Integrity Number of seven were used for microarray hybridization [19].

### Affymetrix Microarrays

Samples meeting the RNA quality criteria were analyzed by microarray (*Sod1* ^−/−^ mice at 8 mo (n = 6) or 20 mo (n = 5); *Sod1* ^+/+^ mice at 8 mo (n = 6), 20 mo (n = 6), and 30 mo (n = 5)). Total RNA (75 ng) was amplified and biotin labeled using the Ovation Biotin-RNA Amplification and Labeling System (NuGEN Technologies, Inc., San Carlos, CA) according to the manufacturer’s protocol. Amplification and hybridization were performed by the University of Michigan Comprehensive Cancer Center Affymetrix and Microarray Core Facility (University of Michigan, Ann Arbor, MI) using the Affymetrix GeneChip Mouse Genome 430 2.0 Array. The intensities of the target hybridizations to their respective probe features were detected by laser scan of the array. Image files were generated by Affymetrix GeneChip software (MAS5).

The Affymetrix raw data files (CEL files) were analyzed using a local copy of GenePattern, a bioinformatics platform from the Broad Institute [20]. The samples were Robust Multi-array Average (RMA) normalized using the BrainArray Custom Chip Definition File (CDF) version 15 [21]. Microarray quality was assessed using the AffyAnalysisQC R package (http://arrayanalysis.org/) with Bioconductor [22]. Arrays deemed as potential outliers by two or more quality assessment modules in the AffyAnalysisQC package were excluded.

### Microarray Data Analysis

#### Identification of differentially expressed genes (DEGs)

DEGs were obtained either between genotypes (*Sod1^+/+^* and *Sod1^−/−^*) at the same age or between different ages (2, 20, and 30 mo) within each genotype. Differential expression of genes was determined using the Intensity-Based Moderated T-statistic (IBMT) test [23] with a false discovery rate (FDR)<0.05.

#### Functional annotation and enrichment analyses

The Database for Annotation, Visualization and Integrated Discovery (DAVID) (http://david.abcc.ncifcrf.gov) [24] was used to identify over-represented biological functions and pathways among the DEGs. The p-values for annotation terms in DAVID were determined using a modified Fisher’s Exact Test, and terms with a Benjamini-Hochberg corrected P-value<0.05 were selected as significantly over-represented biological functions. A heat-map was generated using top 10 most over-represented biological functions in each DEG set, clustered based on the significance values (log-transformed P-values).

#### Literature survey of cholesterol-related DEGs

To prioritize the DEGs for protein-assay validation, we surveyed the literature using a literature mining tool to examine the relevance and importance of our DEGs to the enriched biological function, *cholesterol metabolism*. SciMiner (http://jdrf.neurology.med.umich.edu/SciMiner) [25], our in-house web-based literature mining tool, was used to identify genes and how often they appear in the cholesterol- and aging-related literature, defined by a PubMed query of “aging AND cholesterol”. As of 09/12/2012, the PubMed query resulted in 5,257 papers, from which SciMiner identified 1,179 genes. The number of papers for each gene was statistically evaluated using a Fisher’s Exact Test against the frequency in the complete PubMed abstracts to determine its relative importance to the topic [26]. Table S1 includes the number of papers and p-value from the statistical test for each DEG, if included in the literature-mined 1,179 genes.

### Real-time qRT-PCR

The expression of eight of the down-regulated DEGs related to cholesterol/sterol metabolism and two DEGs that were up-regulated with a fold change greater than 4 was confirmed by real-time qRT-PCR using the same samples as the microarray. Reverse transcription was performed using the iScript cDNA Synthesis kit (Bio-Rad, Hercules, CA). Real-time PCR amplification and SYBR Green fluorescence detection were performed using the Applied Biosystems StepOnePlus Real-time PCR System (Life Technologies Corporation, Carlsbad, CA). The fluorescence threshold value (C_T_) was calculated using StepOne system software. The mRNA levels were normalized to an endogenous reference gene [glyceraldehyde-3-phosphate dehydrogenase (*Gapdh*);/cycle threshold (C_T_)] and then relative to a control group (C_T_), and were expressed as 2^−/C^_T_. The average was calculated from two runs per sample.

### Analysis of Axons and Myelin

The spinal cord ventral roots were processed by the MIL (University of Michigan, Ann Arbor, MI) for transmission electron microscopy (TEM). The samples were rinsed in PBS and post-fixed in 1% osmium tetroxide. After rinsing again in PBS and distilled H_2_O, the samples were stained in aqueous 3% uranyl acetate. The samples were dehydrated in ethanol, propylene oxide, and infiltrated with Spurr’s resin. After polymerizing for 24 h at 60°C, the samples were sectioned using an ultra-microtome at 500 nm for thick sections and 90 nm for thin sections. Images were captured on a Philips CM-100 TEM with a digital camera.

The area and equivalent diameter of the inner axon and the axon (including the myelin) was determined using MetaMorph image analysis software (Molecular Devices Corporation, Sunnyvale, CA). A blinded observer assessed at least 3 images per animal from the proximal and distal ventral root. The area of myelin was calculated by subtracting the inner axonal area from the total axonal area.

### Analysis of Cell Body of Motor Neurons

For light microscopy, sections of the lumbar spinal cord (1 µm) were stained with toluidine blue to determine size and number of motor neurons per our published protocols [27]. Bright field images were captured on a Nikon Microphot FXA microscope with a SPOT-RT digital camera. The resulting images were analyzed for area (µm^2^) using MetaMorph. For TEM, the spinal cord was processed by MIL and imaged as described above for the ventral roots.

### Statistical Analysis

Data analyses were performed using Prism, version 5 (GraphPad Software, Inc., La Jolla, CA). Assumptions about Gaussian distribution of data were made using the D’Agostino & Pearson omnibus normality test. Groups were compared using the ANOVA test using either age or genotype as the independent variable. The t-test was used when appropriate and for the comparison of *Sod1* ^−/−^ mice at 20 mo to *Sod1* ^+/+^ mice at 30 mo. All values are reported with standard error of the mean.

## Results

### Levels of Oxidative Damage in Sensory and Motor Neurons

**Figure 1:**
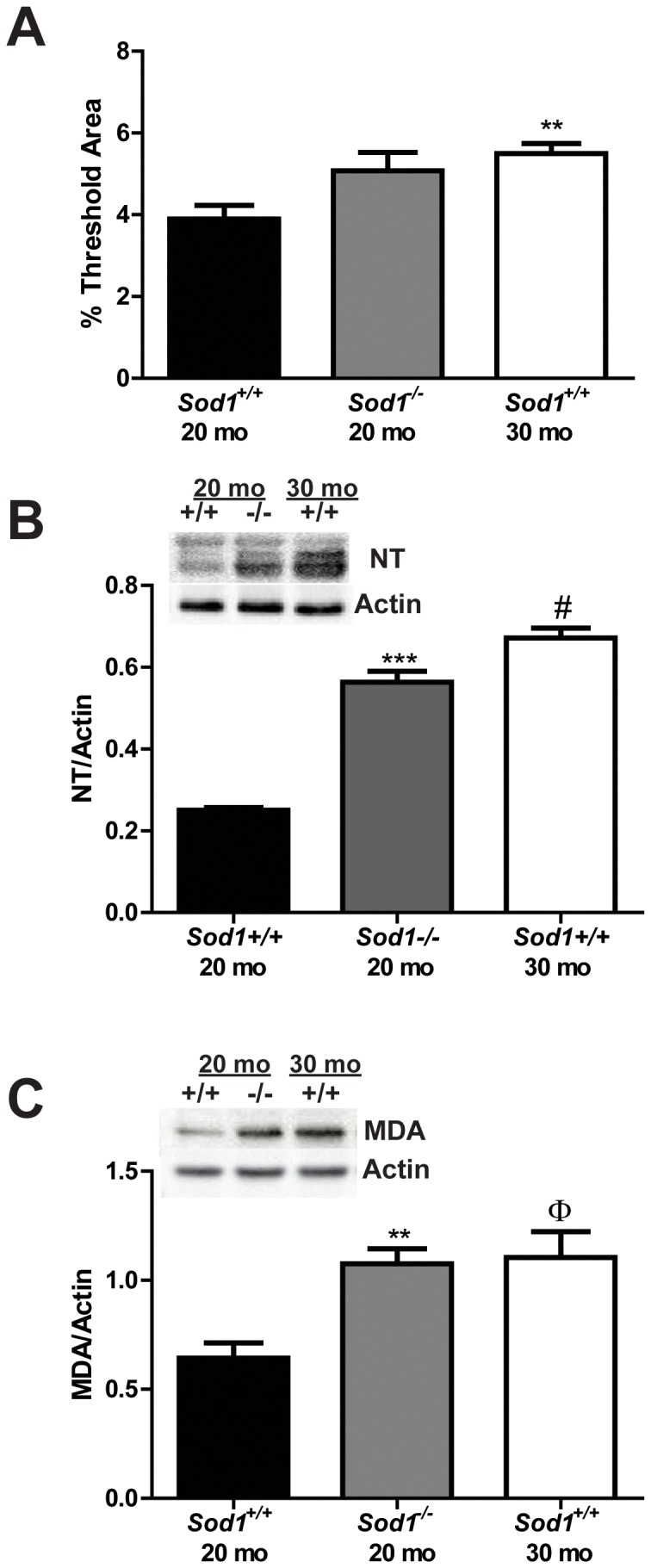
Assessment of oxidative damage in the cell body of sensory neurons. Oxidative damage was assessed in DRG neurons of *Sod1* mice by A) quantitatively assessing the autofluorescence of lipofuscin, and by western immunoblotting of B) nitrated protein (nitrotyrosine; NT) and C) oxidative lipid degradation (malondialdehyde; MDA). The *Sod1* ^+/+^ mice at 20 mo, *Sod1* ^−/−^ mice at 20 mo, and *Sod1* ^+/+^ mice at 30 mo are represented by white, black, and gray bars, respectively. **p<0.01 and ***p<0.0001 compared to age-matched *Sod1* ^+/+^ mice; Φp<0.05 and ^#^p<0.0001 compared to *Sod1* ^+/+^ mice at 20 mo; n≥4.

To determine whether the systemic increase in oxidative stress leads to oxidative damage in the cell bodies of sensory neurons (dorsal root ganglia; DRG) and in the motor neuron micro-environment (spinal cord) in *Sod1* ^+/+^ and *Sod1* ^−/−^ mice, we quantitatively assessed oxidative damage by lipofuscin autofluorescence and western immunoblotting of nitrated proteins and lipid peroxidation, which is oxidative lipid degradation. We did not observe a significant increase in oxidative damage with lipofuscin, nitrated proteins, or lipid peroxidation between the *Sod1^−/−^* mice and the *Sod1^+/+^* mice at 8 mo (Figure S1). The levels of liposfuscin in sensory neurons were increased 1.3-fold in the *Sod1^−/−^* mice at 20 mo (not significant), and 1.4-fold in the *Sod1^+/+^* mice at 30 mo compared with the *Sod1^+/+^* mice at 20 mo (Figure 1A). A 2-fold increase in nitrated proteins, indicative of nitrosative stress, and a 1.7-fold increase in lipid peroxidation was revealed in sensory neurons of the *Sod1* ^−/−^ mice at 20 mo compared to the *Sod1* ^+/+^ mice at 20 mo and 30 mo (Figures 1B and C, respectively). Because oxidative stress is known to induce programmed cell death in a number of cell types, including sensory neurons [28], we utilized western immunoblotting to examine the cleavage of capsase-3, an indicator of apoptosis. We did not observe a significant difference in cleaved caspase-3 in sensory neurons in the *Sod1* ^−/−^ mice at 20 mo or the *Sod1* ^+/+^ mice at 30 mo (data not shown).

**Figure 2:**
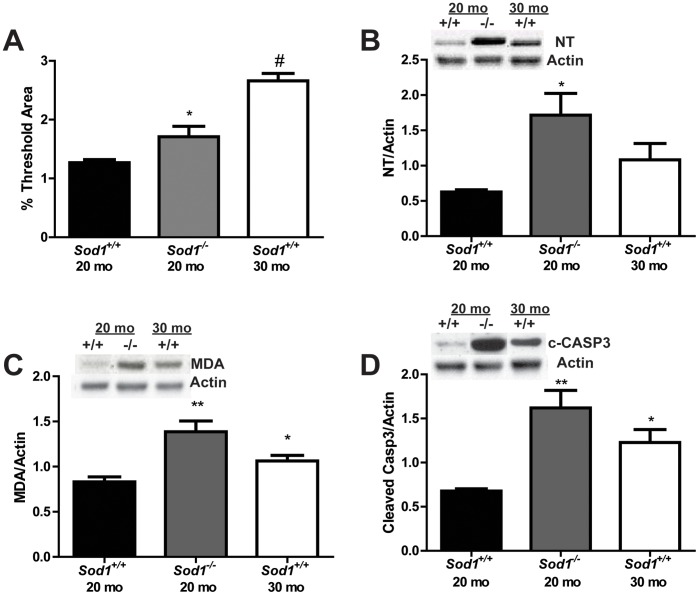
Assessment of oxidative damage in the motor neuron micro-environment. Oxidative damage was assessed in the spinal cord of in *Sod1* mice by A) quantitatively assessing the autofluorescence of lipofuscin, and by western immunoblotting of B) nitrated protein (NT) and C) oxidative lipid degradation (MDA). The *Sod1* ^+/+^ mice at 20 mo, *Sod1* ^−/−^ mice at 20 mo, and *Sod1* ^+/+^ mice at 30 mo are represented by white, black, and gray bars, respectively. D) Apoptosis was assessed in the motor neurons of *Sod1* mice by cleaved caspase-3 western immunoblotting. Densitometry revealed an increase in cleaved caspase-3 in the in *Sod1* ^−/−^ mice at 20 mo compared to the *Sod1* ^+/+^ mice at 20 mo. *p<0.05, **p<0.01; n≥4 for all groups.

We also examined oxidative damage in the motor neuron micro-environment by quantitatively assessing lipofuscin, nitrated proteins, and lipid peroxidation. We did not observe a significant increase in oxidative damage with lipofuscin, nitrated proteins, or lipid peroxidation between the *Sod1^−/−^* mice and the *Sod1^+/+^* mice at 8 mo (Figure S2). The levels of liposfuscin in the motor neuron micro-environment were increased 1.3-fold in the *Sod1^−/−^* mice at 20 mo and 2.1-fold in the *Sod1^+/+^* mice at 30 mo compared with the *Sod1^+/+^* mice at 20 mo (Figure 2A). Nitrated proteins were increased 2.7 fold in the motor neuron environment of *Sod1* ^−/−^ mice at 20 mo compared to *Sod1* ^+/+^ mice at 20 mo (Figure 2B). Furthermore, oxidative lipid degradation was increased 1.7- and 1.3-fold in the motor neuron environment of *Sod1^−/−^* mice at 20 mo and *Sod1^+/+^* mice at 30 mo, respectively, compared to the *Sod1^+/+^* mice at 20 mo (Figure 2C). The cleavage of caspase-3 was increased in the motor neuron environment in the *Sod1* ^−/−^ mice at 20 mo compared to the *Sod1* ^+/+^ mice at 20 mo (Figure 2D). These data suggest that oxidative stress is more detrimental to motor neurons than sensory neurons.

### Sensory and Motor Nerve Function

**Figure 3:**
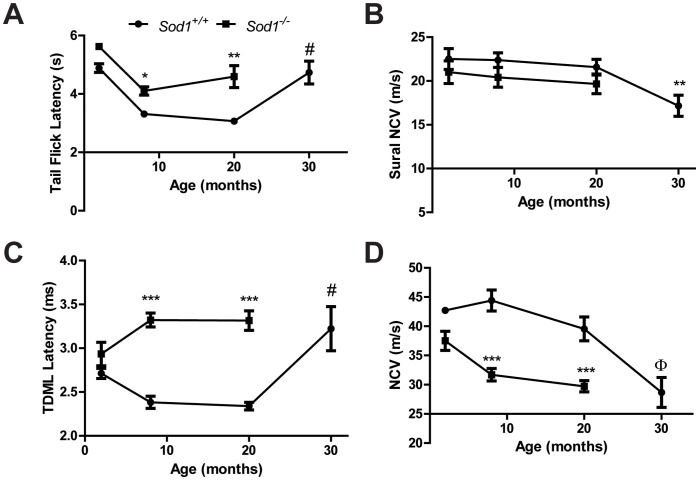
Behavioral and functional measures of sensory and motor nerve function in *Sod1* mice. A) The behavioral tail flick response to a thermal stimulus in the *Sod1* ^+/+^ mice at 2, 8, 20, and 30 mo (n = 7, 16, 10, and 7, respectively) and *Sod1* ^−/−^ mice at 2, 8 and 20 mo (n = 4, 21, and 8, respectively) is a measure of both sensory and motor nerve function. B) Sensory nerve function was assessed using sural nerve conduction velocity (NCV) in the *Sod1* ^+/+^ mice at 8, 20, and 30 mo (n = 13, 9, and 12, respectively) and *Sod1* ^−/−^ mice at 8 and 20 mo (n = 10 and 7, respectively). Motor nerve function was examined by measuring C) Tail distal motor latency (TDML) in the *Sod1* ^+/+^ mice at 2, 8, 20, and 30 mo (n = 8, 21, 12, and 9, respectively) and *Sod1* ^−/−^ mice at 2, 8 and 20 mo (n = 6, 17 and 4) and by measuring D) sciatic motor NCV in the *Sod1* ^+/+^ mice at 2, 8, 20, and 30 mo (n = 7, 15, 7, and 9, respectively) and *Sod1* ^−/−^ mice at 2, 8 and 20 mo (n = 6, 19 and 6). *p<0.05, **p<0.01, and ***p<0.0001 compared to age-matched *Sod1* ^+/+^ mice; Φp<0.01 and ^#^p<0.0001 compared to *Sod1* ^+/+^ mice at 20 mo.

To assess changes in nerve function, we assessed thermal analgesia in the *Sod1* ^+/+^ and *Sod1* ^−/−^ mice using tail flick latency, as a greater latency represents nerve dysfunction. Tail flick latencies (s) were not significantly different in the *Sod1* ^−/−^ and *Sod1* ^+/+^ mice at 2 mo (5.6±0.06 and 4.9±0.15, respectively), but were significantly increased in the *Sod1* ^−/−^ mice at both 8 mo (4.0±0.18) and 20 mo (4.3±0.42) compared to the *Sod1* ^+/+^ mice at 8 mo (3.2±0.08) and 20 mo (2.8±0.10). The tail flick latency significantly increased with age in the *Sod1* ^+/+^ and *Sod1* ^−/−^ mice at 8 mo compared to the 2 mo, and *Sod1* ^+/+^ mice at 30 mo compared to the 20 mo (Figure 3A).

Thermal analgesia is a spinal reflex and requires proper function of both motor and sensory components. To assess specific differences in these neuronal populations, we first characterized the sensory nerve function using sural NCV (m/s). Sural NCV was not significantly different in the *Sod1* ^−/−^ mice at 8 mo (20.1±1.7) or 20 mo (20.7±1.7) compared to the *Sod1* ^+/+^ mice at 8 mo (22.9±1.1) or 20 mo (22.0±1.5); however, the *Sod1^+/+^* mice at 30 mo (17.2±1.2) had a sensory deficit compared with the *Sod1^+/+^* mice at 20 mo (Figure 3B).

To assess functional changes in motor nerve function, motor latency and NCV were evaluated. Tail distal motor latency (TDML; ms) was comparable in the *Sod1* ^−/−^ and *Sod1* ^+/+^ mice at 2 mo (2.9±0.13 and 2.7±0.06, respectively). The latency was significantly increased in the *Sod1* ^−/−^ mice at 8 mo (3.4±0.09) and 20 mo (3.4±0.09) compared to the *Sod1* ^+/+^ mice at 8 mo (2.4±0.07) and 20 mo (2.3±0.04). Additionally, TDML significantly increased with age in the *Sod1* ^+/+^ mice at 30 mo compared to the 20 mo and in the *Sod1* ^−/−^ mice at 8 mo compared to the 2 mo (Figure 3C). A similar trend was observed in the SMNCV (m/s) with no change in the *Sod1* ^−/−^ compared to *Sod1* ^+/+^ mice at 2 mo (37.5±1.7 and 42.7±1.7, respectively). A reduction was observed in the SMNCV in *Sod1* ^−/−^ mice at 8 mo (30.6±1.1) and 20 mo (28.3±2.8) and in the *Sod1* ^+/+^ mice at 30 mo (28.7±2.6) compared to the *Sod1* ^+/+^ mice at 8 mo (44.6±1.8) and at 20 mo (42.9±2.1). SMNCV also decreased with age in the *Sod1* ^+/+^ mice at 30 mo compared to the 20 mo and in the *Sod1* ^−/−^ mice at 8 mo compared to the 2 mo (Figure 3D). These data indicate that the aged *Sod1* ^+/+^ mice (30 mo) and the *Sod1^−/−^* mice have deficits in motor nerve function but not sensory nerve function. Thus, we next characterized and compare the alterations in motor neurons in the *Sod1* ^−/−^ mice compared with the *Sod1* ^+/+^ mice.

### Mechanisms Contributing to Deficits in the Peripheral Nerve

**Figure 4:**
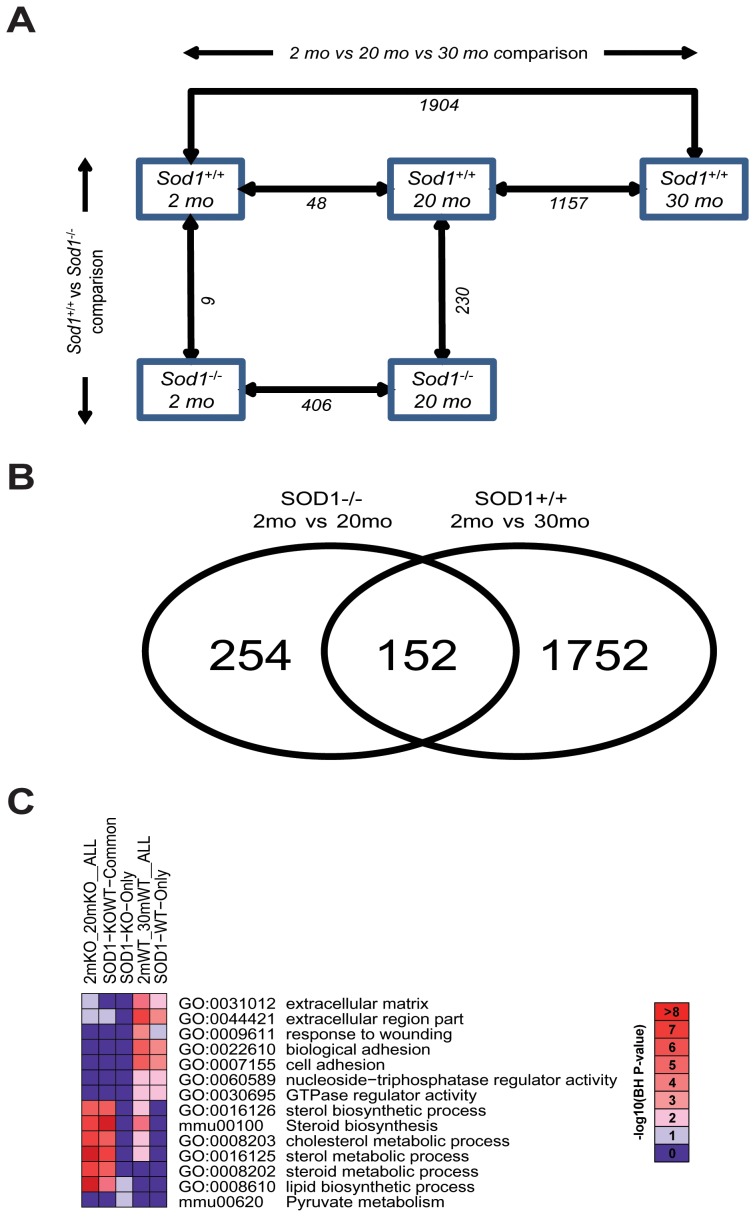
Assessment of gene expression in the sciatic nerve during aging. Alterations in the gene expression in the sciatic nerve during normal aging (*Sod1^+/+^* mice at 2 mo and 30 mo) and during increased oxidative stress (*Sod1^−/−^* mice at 2 mo and 20 mo), were assessed using Affymetrix microarray technology. A) A comparison of the data sets for differential gene expression and the number of DEGs obtained in each comparison. IBMT FDR 5% was used to determine DEGs in age (horizontal) and genotype (vertical) group comparisons. The numbers of identified DEGs at each comparison are noted under the arrows. B) A Venn-diagram of the 2 mo/30 mo *Sod1^+/+^* mouse comparison (normal aging) and the 2 mo/20 mo *Sod1^−/−^* mouse comparison (increased oxidative stress). C) The five DEG sets, including the two original DEG sets (denoted as 2mKO_20mKO__ALL (406 DEGs) and 2mWT_30mWT__ALL (1,904 DEGs)) and three subsets (common DEGs (denoted as SOD1-KOWT-Common (152 DEGs))) or unique DEGs to either set (SOD1-KO-Only (254 DEGs) or SOD1-WT-Only (1,752 DEGs)) from Figure 3B, were subject to functional enrichment analysis using DAVID. Benjamini-Horchberg (BH)-corrected P-values of the top 10 most significant functional terms are represented in a heat-map with –log_10_(BH-corrected P-value) as color index and number.

To examine the potential mechanisms underlying the contribution of oxidative stress to age-related deficits in the peripheral nervous system, we employed microarray technology and bioinformatics approaches to systematically analyze the changes in the gene expression profiles in the SCN, which contain peripheral axons. Figure 4A illustrates the comparisons between age (normal aging in the *Sod1^+/+^* mice at 2 mo versus 30 mo; oxidative stress-mediated effects in the *Sod1^−/−^* mice at 2 mo versus 20 mo) and genotype (*Sod1^+/+^* and *Sod1^−/−^*) groups and the number of DEGs determined by IBMT (see Table S1 for a complete list of the DEGs). During normal aging, only 48 genes are differently expressed in the 20 mo compared with the 2 mo *Sod1^+/+^* mice; however, 1,904 genes were differentially expressed in the 30 mo compared with the 2 mo *Sod1^+/+^* mice. At 2 mo, only 9 genes were significantly different between *Sod1^+/+^* and *Sod1^−/−^* mice, suggesting that the lack of *Sod1* does not affect early development. The changes in genes expression in the peripheral nerve mediated by oxidative stress includes 406 DEGs in the 20 mo compared with the 2 mo *Sod1^−/−^* mice.

To determine potential mechanisms for oxidative stress-mediated effects in the peripheral nerve, we focused on the 2 mo/30 mo *Sod1^+/+^* mouse comparison and the 2 mo/20 mo *Sod1^−/−^* mouse comparison. Approximately one-third of the DEGs in the *Sod1^−/−^* mouse overlapped with the *Sod1^+/+^* mice (Figure 4B). A functional enrichment analysis was performed to identify significantly over-represented biological functions in terms of Gene Ontology and pathway terms of these DEGs, and a heat-map was generated to summarize the most significantly over-represented functions (Figure 4C). The common DEGs between the two sets were highly enriched with processes related to cholesterol and sterol metabolism.

**Figure 5:**
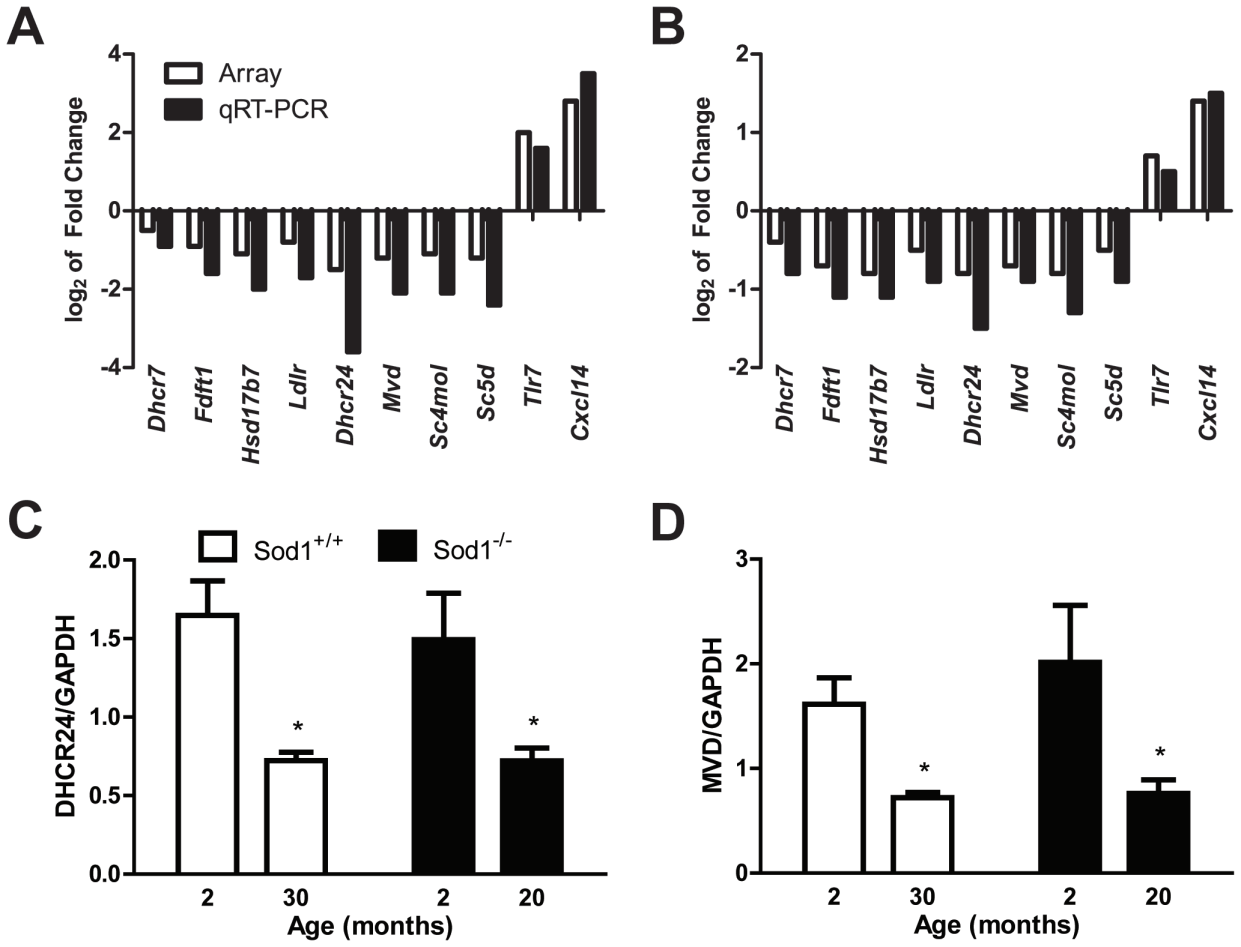
qRT-PCR verification of the expression levels of the DEGs in the sciatic nerve. The log_2_ transformation of the fold-change of the relative mRNA expression levels in the 10 DEGs selected for qRT-PCR (black bars) parallels the direction of changes in the microarray (white bars) in A) normally aged mice, the *Sod1^+/+^* mice at 20 mo compared with 2 mo mice, and B) in a model of increased oxidative stress, the *Sod1^−/−^* mice at 30 mo compared with 2 mo mice. Western immunoblotting densitometry was used to validate the protein expression levels of two genes that were differentially expressed and encode enzymes in the cholesterol/sterol synthesis pathway, C) DHCR24 and D) MVD; *p<0.05 and **p<0.01.

To validate the microarray expression data, qRT-PCR was performed on eight DEGs related to cholesterol and sterol metabolism (all down-regulated) and two DEGs related to inflammation due to the high up-regulated fold change (>4): 7-dehydrocholesterol reductase, *Dhcr7* (−1.41-fold change in *Sod1^+/+^* and −1.30-fold change in *Sod1^−/−^* mice); farnesyl diphosphate farnesyl transferase 1, *Fdft1* (−1.86-fold change in *Sod1^+/+^* and −1.58-fold change in *Sod1^−/−^* mice); hydroxysteroid (17-beta) dehydrogenase 7, *Hsd17b7* (−2.18-fold change in *Sod1^+/+^* and −1.77-fold change in *Sod1^−/−^* mice); low density lipoprotein receptor, *Ldlr* (−1.75-fold change in *Sod1^+/+^* and −1.42-fold change in *Sod1^−/−^* mice); 24-dehydrocholesterol reductase, *Dhcr24* (−2.77-fold change in *Sod1^+/+^* and −1.79-fold change in *Sod1^−/−^* mice); mevalonate (diphospho) decarboxylase, *Mvd* (−2.33 fold change in *Sod1^+/+^* and −1.66-fold change in *Sod1^−/−^* mice); sterol-C4-methyl oxidase-like, *Sc4mol* (−2.07-fold change in *Sod1^+/+^* and −1.75-fold change in *Sod1^−/−^* mice); sterol-C5-desaturase (fungal ERG3, delta-5-desaturase) homolog (S. cerevisae), *Sc5d* (−2.31-fold change in *Sod1^+/+^* and −1.38-fold change in *Sod1^−/−^* mice); toll-like receptor 7, *Tlr7* (4.11-fold change in *Sod1^+/+^* and 1.62-fold change in *Sod1^−/−^* mice); chemokine (C-X-C motif) ligand 14, *Cxcl14* (7.02-fold change in *Sod1^+/+^* and 2.66-fold change in *Sod1^−/−^* mice). All ten tested genes demonstrated significant differential expression and the fold changes paralleled the directionality of down- or up-regulation in the microarray results (Figures 5A and B).

To confirm the biological relevance of the cholesterol/sterol metabolism-related down-regulated DEGs during normal aging in the *Sod1^+/+^* mice and aging with increased oxidative stress in the *Sod1^−/−^* mice, we evaluated the protein levels of two DEGs–DHCR24 (end of the pathway) and MVD (beginning of the pathway), which were significantly over-represented in the literature based on our text mining tool (SciMiner) results [25]. The relative protein levels of DHCR24 decreased by 56% during normal aging in the *Sod1^+/+^* mice at 30 mo compared with the *Sod1^+/+^* mice at 2 mo (0.72±0.54 and 1.6±0.22, respectively), and by 52% in the *Sod1^−/−^* mice at 20 mo compared with the *Sod1^−/−^* mice at 2 mo (0.72±0.082 and 1.5±0.30, respectively; Figure 5C). Similarly, the relative protein levels of MVD decreased by 55% during normal aging in the *Sod1^+/+^* mice at 30 mo compared with the *Sod1^+/+^* mice at 2 mo (0.72±0.05 and 1.61±0.25, respectively), and by 62% in the *Sod1^−/−^* mice at 20 mo compared with the *Sod1^−/−^* mice at 2 mo (0.76±0.13 and 2.0±0.54, respectively; Figure 5D).

### Structural Deficits in Axons, Myelin, and the Cell Body of Motor Neurons

**Figure 6:**
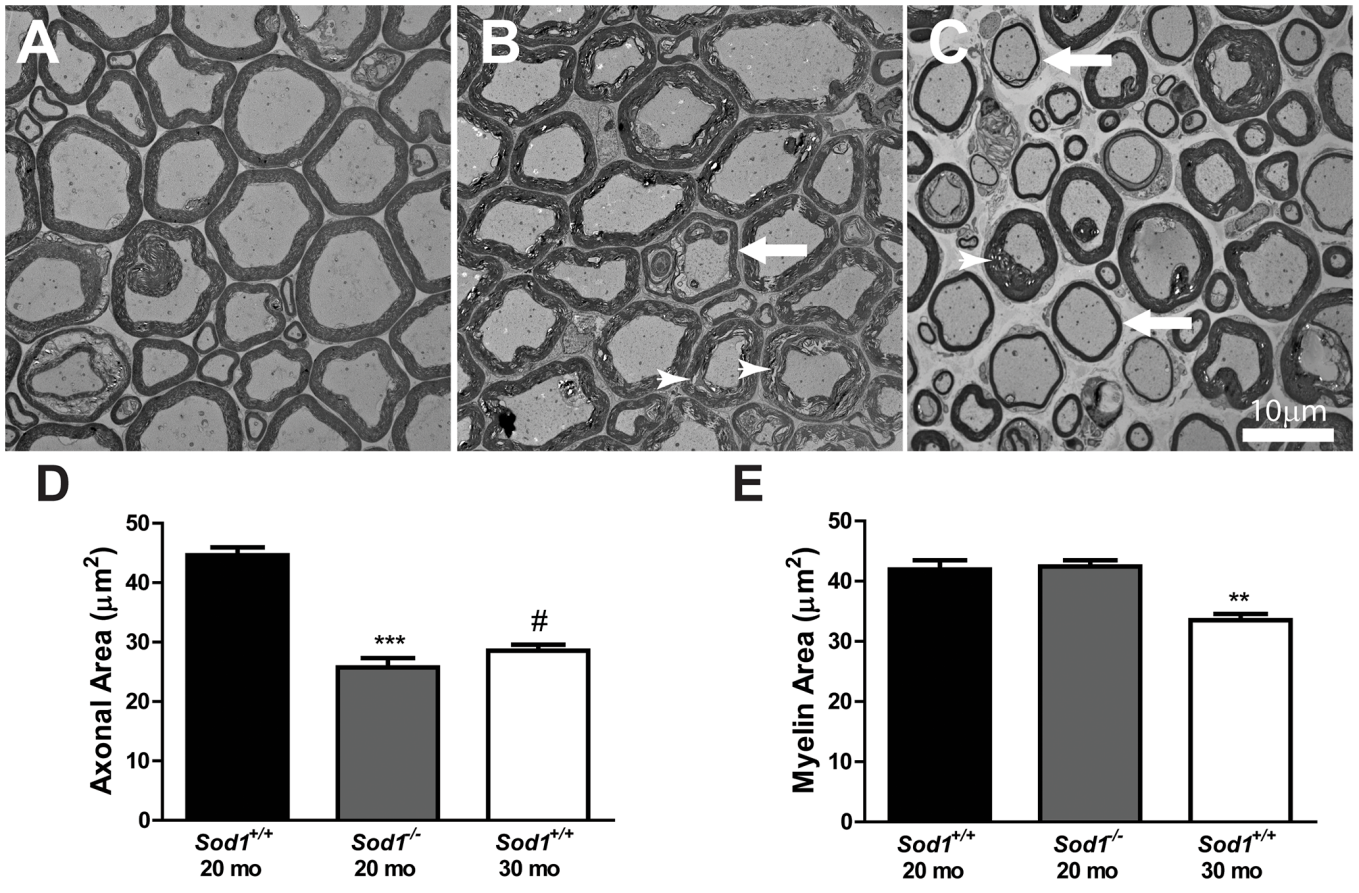
Structural deficits in the axons and myelin of motor neurons in the *Sod1* mice. Transmission electron microscopy (TEM) of the ventral roots from A) *Sod1* ^+/+^ mice at 20 mo, B) *Sod1* ^−/−^ mice at 20 mo, and C) *Sod1* ^+/+^ mice at 30 mo revealed thinning myelin (arrow) and delaminating myelin (arrow head). Scale bar = 10 µm. The area of D) axons and E) myelin in the ventral root was measured by light microscopy in the *Sod1* ^+/+^ mice at 20 and 30 mo (n = 9, 3, and 5, respectively) and *Sod1* ^−/−^ mice at 20 mo (n = 19, and 10). **p<0.001, ***p<0.0001, and ^#^p<0.0001 compared to *Sod1* ^+/+^ mice at 20 mo.

Abnormalities in cholesterol metabolism can lead to abnormalities in axons and myelin [29], [30]. Thus, the deficits we observed in the NCV may be indicative of abnormalities in axons or myelin. Analysis of the spinal cord ventral root did not reveal a significant difference between the axonal area or myelin area in the *Sod1* ^−/−^ mice at 8 mo compared with the *Sod1* ^+/+^ mice at 8 mo (data not shown). On the other hand, structural changes in the *Sod1* ^−/−^ mice at 20 mo included alterations in axonal size and delaminating myelin, and structural changes in the *Sod1* ^+/+^ mice at 30 mo demonstrated alterations in axonal size and thinning myelin (Figure 6A–C). Quantitative analysis of the axonal area in the ventral root revealed a 1.7-fold decrease in the *Sod1* ^−/−^ mice at 20 mo and a 1.6-fold decrease the *Sod1* ^+/+^ mice at 30 mo compared with the *Sod1* ^+/+^ mice at 20 mo (Figure 6D). Although the area of myelin was not significantly different in the *Sod1* ^−/−^ mice at 20 mo compared with the *Sod1* ^+/+^ mice at 20 mo, the area of myelin decreased 1.3-fold in the *Sod1* ^+/+^ mice at 30 mo compared with the *Sod1* ^+/+^ mice at 20 mo (Figure 6E).

**Figure 7:**
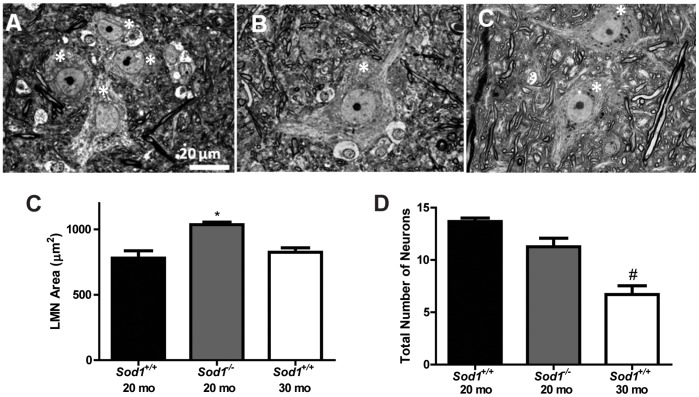
Structural deficits in the cell body of motor neurons using light microscopy. The cell body of lower motor neurons (LMN; asterisks), located in the spinal cord, was assessed by light microscopy. A) *Sod1* ^+/+^ mice at 20 mo, B) *Sod1* ^−/−^ mice at 20 mo, and C) *Sod1* ^+/+^ mice at 30 mo. Scale bar = 20 µm. D) The area of LMN in the spinal cord (L_5_) and E) the number of LMN was quantified. *p<0.05 compared to age-matched *Sod1* ^+/+^ mice and ^#^p<0.0001 compared to *Sod1* ^+/+^ mice at 20 mo.

A deficit in the axon or myelin is not necessarily indicative of cell body damage. Therefore, we assessed anatomical alterations in the cell body of motor neurons utilizing light microscopy. Analysis of the lumbar spinal cord did not reveal any difference in area or total number of motor neurons in the *Sod1* ^−/−^ mice at 8 mo compared with the *Sod1* ^+/+^ mice at 8 mo (data not shown); however, analysis of the aged mice revealed an increase in the area of motor neurons in *Sod1* ^−/−^ mice at 20 mo compared to the *Sod1* ^+/+^ at 20 mo and 30 mo (Figure 7A–D), with a trending decrease in the total number of L_5_ lower motor neurons in the *Sod1* ^−/−^ mice at 20 mo and a significant decrease in the *Sod1* ^+/+^ at 30 mo compared to the *Sod1* ^+/+^ at 20 mo (Figure 7E).

**Figure 8:**
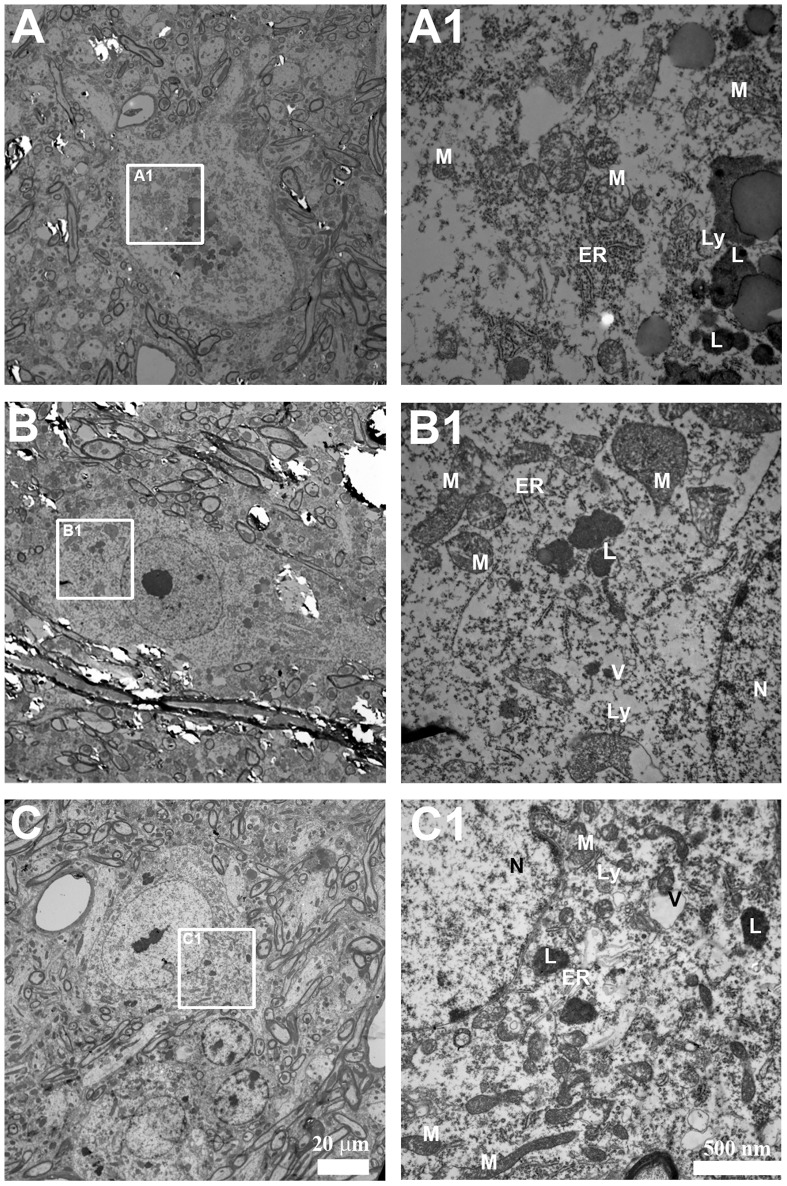
Ultrastructural deficits in the cell body of motor neurons using TEM. Representative images of motor neurons in A, A1) *Sod1* ^+/+^ mice at 20 mo, B, B1) *Sod1* ^−/−^ mice at 20 mo, and C, C1) *Sod1* ^+/+^ mice at 30 mo. Scale bar = 20 µm (A, B, and C) and 500 nm (A1, B1, and C1). ER = endoplasmic reticulum; L = lipofuscin; Ly = lysosome; M = mitochondria; N = nucleus; v = vacuoles.

To further explore the anatomical alterations in the cell body of motor neurons we also utilized transmission electron microscopy (TEM; Figure 8). TEM of motor neurons in the *Sod1* ^−/−^ mice at 20 mo compared with the *Sod1* ^+/+^ mice at 20 mo revealed a lack of distinct Nissl bodies associated with the endoplasmic reticulum, darkening of the cytoplasm, disorganized organelles, and formation of vacuoles. Similar to the *Sod1* ^−/−^ mice at 20 mo, although less pronounced, TEM of motor neurons in the *Sod1* ^+/+^ mice at 30 mo also revealed a lack of Nissl bodies associated with the endoplasmic reticulum, darkening of the cytoplasm, disorganized organelles, and the formation of vacuoles compared with the *Sod1* ^+/+^ mice at 20 mo.

## Discussion

Increasing oxidative stress is implicated in normal aging, sarcopenia, and decreased neuromuscular function. Building on our previous observation that denervation of the muscle resulted in the loss of muscle fibers and muscle atrophy [10], [12], in the current study we compared normal age-related deficits in the peripheral nervous system superimposed on those observed in an established model of increased systemic oxidative stress, the *Sod1* ^−/−^ mouse. We demonstrate increased oxidative damage in sensory neurons and in the motor neuron micro-environment, as well as deficits in peripheral nerve function, in *Sod1* ^−/−^ mice similar to normal aging observed in wild-type, *Sod1* ^+/+^ mice.

The increase in oxidative damage in the *Sod1^−/−^* mice and aged *Sod1^+/+^* mice parallels an increase in thermal thresholds in the tail, a response that requires both sensory and motor neurons. These changes in thresholds may reflect length-dependent changes, similar to those observed in diabetic neuropathy [17], [31]. To determine whether the changes in thermal threshold are due to alterations in sensory and/or motor nerve function, each component was evaluated separately. Sensory nerve function was evaluated using sural NCV, which did not demonstrate a significant difference between the *Sod1^+/+^* and *Sod1^−/−^* mice. This is consistent with a previous study that demonstrated that the compound action potentials in the sural nerve of *Sod1* ^−/−^ mice at 5–7 mo were not significantly different than their wild-type littermates; however, data in older mice were not evaluated in that study [11]. In contrast to sensory nerve function, a significant and earlier onset of motor deficits observed in the *Sod1* ^−/−^ mice compared to a deficit in the *Sod1* ^+/+^ mice only at 30 mo, is consistent with previous observations of significant deficits in NCV involving the caudal nerve (tail) and motor axons of the tibial nerve (SMNCV) [11], [32], [33]. The lack of phenotype in sensory nerve function compared with motor nerve function is not due to a difference in the levels of oxidative damage, as both sensory and motor neurons have elevated levels of oxidative damage. Our study indicates that motor neurons are more susceptible than sensory neurons to systemic, chronic oxidative stress and damage in the *Sod1* ^−/−^ mice. Our data are in general agreement with a previous study, which demonstrated greater dysfunction in the ventral root (motor) compared to the dorsal root (sensory) axons in *Sod1* ^−/−^ mice [11]. The motor nerve function deficits in our *Sod1^−/−^* model of increased oxidative stress resembles deficits observed during normal aging in the *Sod1^+/+^* mouse, suggesting that oxidative stress plays a role in the age-related decline in motor function.

NCV deficits in the *Sod1^−/−^* mice may be due to either axonal degeneration or demyelination of the peripheral nerve. We used microarray technology, to correlate the functional deficits with alterations in gene expression in the peripheral (sciatic) nerve, in which Schwann cells are the primary cellular component [34], from 2 mo to 30 mo in the *Sod1^+/+^* mouse and from 2 mo to 20 mo in the *Sod1^−/−^* mouse. Schwann cells support the preservation and function of peripheral nerve axons [35]. Bioinformatics analyses revealed that the most significantly enriched processes that overlapped in these two DEG sets are associated with cholesterol/sterol metabolism. A recent study demonstrated that abnormal lipid metabolism leads to axonal degeneration [36]. Cholesterol synthesis is vital for peripheral nervous system function, especially for axonal support and myelination by Schwann cells [37]. The transcriptional down-regulation of genes involved in the cholesterol synthesis pathway leads to myelin degeneration [38]. Furthermore, during loss of axonal contact with the motor endplate causes Schwann cells to reduce expression of the two major myelin proteins of the peripheral nervous system, myelin protein zero (*P0*) and peripheral myelin protein 22 (*Pmp22*). Interestingly, *P0* is down-regulated in both the *Sod1^+/+^* mice at 30 mo compared to 2 mo and in the *Sod1^−/−^* mice at 20 mo compared to the 2 mo. *Pmp22* is down-regulated in the *Sod1^+/+^* mice at 30 mo compared to 2 mo but not in the *Sod1^−/−^* mice at 20 mo compared to the 2 mo. Thus, abnormalities in cholesterol metabolism may lead to abnormalities in both axons and myelin [29], [30].

The survival of motor axons is dependent upon the presence of SOD1 [33]. Thus, it is not surprisingly that there is a loss of innervation in the hindlimb muscle in the *Sod1^−/−^*mice [12], [33]. This loss of innervation provides evidence of axonal degeneration. The reduction we observed in axonal area in the motor axons of the ventral root in *Sod1* ^−/−^ and *Sod1* ^+/+^ mice at 30 mo is in agreement with previous work by Flood and colleagues (1999) in the *Sod1* ^−/−^ mice [11], and provides additional evidence of axonal degeneration. Although demyelination occurred in the normally aged *Sod1^+/+^*, we did not observe a significant difference in the area of myelin in the *Sod1^−/−^* mice. These data suggest that axonal degeneration rather than myelin degeneration occurs during aging associated with increased levels of oxidative damage.

Aberrant cholesterol metabolism is observed in neurodegenerative diseases [39]. A recent report linked aberrant cholesterol metabolism to mitochondrial dysfunction, axonal degeneration, and neuropathy [36]. The ultrastructural evaluation of the cell body of motor neurons in the *Sod1^−/−^* mice compared with the *Sod1^+/+^* mice revealed the presence of abnormally shaped mitochondria. A previous study reported mitochondrial deficits in the *Sod1^−/−^* mice [40]. Thus, the loss of muscle fibers, atrophy, and axonal degeneration observed in the *Sod1^−/−^* mice and during normal aging [12] correlates with deficient cholesterol synthesis. A reduction in the levels of molecules involved in cholesterol trafficking and intracellular accumulations of cholesterol, and its precursors and metabolites were observed in neurons exposed to chronic levels of oxidative stress [41]. Hence, it is possible that the Schwann cells adapt to chronic levels of oxidative stress associated with aging by reducing cholesterol metabolic pathways; however, further studies are required to confirm this interaction.

If oxidative stress is solely responsible for aging, then the following three conditions must also be true: 1) oxidative damage increases with age, 2) manipulations to decrease oxidative stress would result in decreased cellular damage and an increased lifespan, and 3) manipulations that increase lifespan would result in a decrease in oxidative stress [42]. Over-expression of *Sod1*, a manipulation to decrease oxidative stress, increases the lifespan in *Drosophila* [43]; however, the same result was not produced in mice [8], [44]. Although genetic manipulations in animal models that produce an increased lifespan appear to correlate with a reduction in oxidative stress/damage [42], oxidative stress is not solely responsible for aging. This is underscored by the fact that administration of antioxidants in clinical trials have either failed to provide a beneficial effect or significantly increased mortality [45]. Oxidative stress does, however, play a major role and increases with age in both human tissue and animal models [42].

Our study reveals that although the systemic presence of increased oxidative stress in the *Sod1^−/−^* mice is sufficient to cause functional deficits, structural abnormalities correlate with oxidative damage. The *Sod1* ^−/−^ mice are functionally normal at 2 mo of age but display an early onset of decreased motor function by 8 mo similar to the deficits associated with normal aging in the *Sod1* ^+/+^ mice at 30 mo. Studies investigating the differences in antioxidant capacities between motor and sensory neurons may provide useful information for understanding the differences in the sensitivity to oxidative damage. A previous study reported that Schwann cells express different motor and sensory phenotypes that regulate axonal support and regeneration [46]; thus, studying the potential differences in these properties in peripheral nerve may provide insight into why motor axons are more susceptible to oxidative damage than sensory axons. Furthermore, our future studies will evaluate the adaption response of Schwann cells *in vitro* to chronic oxidative stress. This is necessary to fully elucidate the role of cholesterol pathways in aging nervous system. Collectively, our data indicate that oxidative stress mediates motor nerve function deficits in the aging peripheral nervous system and that these deficits are due to abnormalities in axonal degeneration.

## Supporting Information

Figure S1 **Assessment of oxidative damage in the cell body of sensory neurons in young** ***Sod1*** **mice.** Oxidative damage was assessed in dorsal root ganglia neurons of 8 mo *Sod1* mice by A) quantitatively assessing the autofluorescence of lipofuscin and western immunoblotting, and densitometry analysis of B) nitrated proteins (nitrotyrosine, NT) and C) oxidative lipid degradation (malondialdehyde, MDA). *Sod1^+/+^* and *Sod1^−/−^* mice are represented by light gray and black/white strip bars, respectively; n≥4.
(TIFF)

Figure S2 **Assessment of oxidative damage in the motor neuron micro-environment in young** ***Sod1*** **mice.** Oxidative damage was assessed in the spinal cord of in 8 mo *Sod1* mice by A) quantitatively assessing the autofluorescence of lipofuscin and western immunoblotting, and densitometry analysis of B) nitrated proteins (NT) and C) oxidative lipid degradation (MDA). *Sod1^+/+^* and *Sod1^−/−^* mice are represented by light gray and black/white strip bars, respectively; n≥4.
(TIFF)

Table S1 **List of DEGs.** Fold-changes are given if the gene is a DEG. DEG/Set count indicates the number of DEG sets (out of 6 sets in total) in which the corresponding gene has been identified as a DEG. The total number of DEGs in each set is denoted in red. The two columns highlighted in yellow are the primary DEG sets used in the present study. Any DEGs that were also identified in the cholesterol- and aging-related literature are noted in the last three columns. #Paper: the number of papers in which the corresponding gene was identified by SciMiner; Enrichment: the fold-enrichment of the frequency (the number of papers with the corresponding gene/total number of cholesterol- and aging-related literature) compared to the frequency of the gene in the whole PubMed abstracts; BH P-value: Benjamini-Hochberg corrected p-value of the Fisher’s exact test to test the significance of the gene in the cholesterol- and aging-related literature. The genes are sorted by ‘DEG count’, and the fold-changes in the ‘2mKO_20mKO’ and ‘20mWT_30mWT’ sets.
(PDF)

## References

[1] Vandervoort AA (2002) Aging of the human neuromuscular system. Muscle Nerve 25: 17–25. 11754180 10.1002/mus.1215

[2] Roubenoff R (2001) Origins and clinical relevance of sarcopenia. Can J Appl Physiol 26: 78–89. 11291626 10.1139/h01-006

[3] Peters A (2002) The effects of normal aging on myelin and nerve fibers: a review. J Neurocytol 31: 581–593. 14501200 10.1023/a:1025731309829

[4] Van Remmen H, Hamilton ML, Richardson A (2003) Oxidative damage to DNA and aging. Exerc Sport Sci Rev 31: 149–153. 12882482 10.1097/00003677-200307000-00009

[5] Sohal RS, Weindruch R (1996) Oxidative stress, caloric restriction, and aging. Science 273: 59–63. 8658196 10.1126/science.273.5271.59 PMC2987625

[6] Beckman KB, Ames BN (1998) The free radical theory of aging matures. Physiol Rev 78: 547–581. 9562038 10.1152/physrev.1998.78.2.547

[7] Salmon AB, Richardson A, Perez VI (2010) Update on the oxidative stress theory of aging: does oxidative stress play a role in aging or healthy aging? Free Radic Biol Med 48: 642–655. 20036736 10.1016/j.freeradbiomed.2009.12.015 PMC2819595

[8] Perez VI, Bokov A, Van Remmen H, Mele J, Ran Q, et al (2009) Is the oxidative stress theory of aging dead? Biochim Biophys Acta 1790: 1005–1014. 19524016 10.1016/j.bbagen.2009.06.003 PMC2789432

[9] Muller FL, Song W, Liu Y, Chaudhuri A, Pieke-Dahl S, et al (2006) Absence of CuZn superoxide dismutase leads to elevated oxidative stress and acceleration of age-dependent skeletal muscle atrophy. Free Radic Biol Med 40: 1993–2004. 16716900 10.1016/j.freeradbiomed.2006.01.036

[10] Jang YC, Lustgarten MS, Liu Y, Muller FL, Bhattacharya A, et al (2010) Increased superoxide in vivo accelerates age-associated muscle atrophy through mitochondrial dysfunction and neuromuscular junction degeneration. FASEB J 24: 1376–1390. 20040516 10.1096/fj.09-146308 PMC2987499

[11] Flood DG, Reaume AG, Gruner JA, Hoffman EK, Hirsch JD, et al (1999) Hindlimb motor neurons require Cu/Zn superoxide dismutase for maintenance of neuromuscular junctions. Am J Pathol 155: 663–672. 10433959 10.1016/S0002-9440(10)65162-0 PMC1866863

[12] Larkin LM, Davis CS, Sims-Robinson C, Kostrominova TY, Remmen HV, et al (2011) Skeletal muscle weakness due to deficiency of CuZn-superoxide dismutase is associated with loss of functional innervation. Am J Physiol Regul Integr Comp Physiol 301: R1400–1407. 21900648 10.1152/ajpregu.00093.2011 PMC3213934

[13] Lebovitz RM, Zhang H, Vogel H, Cartwright J Jr, Dionne L, et al (1996) Neurodegeneration, myocardial injury, and perinatal death in mitochondrial superoxide dismutase-deficient mice. Proc Natl Acad Sci U S A 93: 9782–9787. 8790408 10.1073/pnas.93.18.9782 PMC38506

[14] Van Remmen H, Qi W, Sabia M, Freeman G, Estlack L, et al (2004) Multiple deficiencies in antioxidant enzymes in mice result in a compound increase in sensitivity to oxidative stress. Free Radic Biol Med 36: 1625–1634. 15182862 10.1016/j.freeradbiomed.2004.03.016

[15] Sentman ML, Granstrom M, Jakobson H, Reaume A, Basu S, et al (2006) Phenotypes of mice lacking extracellular superoxide dismutase and copper- and zinc-containing superoxide dismutase. J Biol Chem 281: 6904–6909. 16377630 10.1074/jbc.M510764200

[16] Huang TT, Yasunami M, Carlson EJ, Gillespie AM, Reaume AG, et al (1997) Superoxide-mediated cytotoxicity in superoxide dismutase-deficient fetal fibroblasts. Arch Biochem Biophys 344: 424–432. 9264557 10.1006/abbi.1997.0237

[17] Sullivan KA, Hayes JM, Wiggin TD, Backus C, Su Oh S, et al (2007) Mouse models of diabetic neuropathy. Neurobiol Dis 28: 276–285. 17804249 10.1016/j.nbd.2007.07.022 PMC3730836

[18] Vincent AM, Perrone L, Sullivan KA, Backus C, Sastry AM, et al (2007) Receptor for advanced glycation end products activation injures primary sensory neurons via oxidative stress. Endocrinology 148: 548–558. 17095586 10.1210/en.2006-0073

[19] Schroeder A, Mueller O, Stocker S, Salowsky R, Leiber M, et al (2006) The RIN: an RNA integrity number for assigning integrity values to RNA measurements. BMC Mol Biol 7: 3. 16448564 10.1186/1471-2199-7-3 PMC1413964

[20] Reich M, Liefeld T, Gould J, Lerner J, Tamayo P, et al (2006) GenePattern 2.0. Nat Genet 38: 500–501. 16642009 10.1038/ng0506-500

[21] Dai M, Wang P, Boyd AD, Kostov G, Athey B, et al (2005) Evolving gene/transcript definitions significantly alter the interpretation of GeneChip data. Nucleic Acids Res 33: e175. 16284200 10.1093/nar/gni179 PMC1283542

[22] Gautier L, Cope L, Bolstad BM, Irizarry RA (2004) affy–analysis of Affymetrix GeneChip data at the probe level. Bioinformatics 20: 307–315. 14960456 10.1093/bioinformatics/btg405

[23] Sartor MA, Tomlinson CR, Wesselkamper SC, Sivaganesan S, Leikauf GD, et al (2006) Intensity-based hierarchical Bayes method improves testing for differentially expressed genes in microarray experiments. BMC Bioinformatics 7: 538. 17177995 10.1186/1471-2105-7-538 PMC1781470

[24] Huang da W, Sherman BT, Lempicki RA (2009) Systematic and integrative analysis of large gene lists using DAVID bioinformatics resources. Nat Protoc 4: 44–57. 19131956 10.1038/nprot.2008.211

[25] Hur J, Schuyler AD, States DJ, Feldman EL (2009) SciMiner: web-based literature mining tool for target identification and functional enrichment analysis. Bioinformatics 25: 838–840. 19188191 10.1093/bioinformatics/btp049 PMC2654801

[26] Sims-Robinson C, Zhao S, Hur J, Feldman EL (2012) Central nervous system endoplasmic reticulum stress in a murine model of type 2 diabetes. Diabetologia. 10.1007/s00125-012-2573-6 PMC3391332 22581041

[27] Boulis NM, Turner DE, Imperiale MJ, Feldman EL (2002) Neuronal survival following remote adenovirus gene delivery. J Neurosurg 96: 212–219. 12450285 10.3171/spi.2002.96.2.0212

[28] Russell JW, Golovoy D, Vincent AM, Mahendru P, Olzmann JA, et al (2002) High glucose-induced oxidative stress and mitochondrial dysfunction in neurons. FASEB J 16: 1738–1748. 12409316 10.1096/fj.01-1027com

[29] de Chaves EI, Rusinol AE, Vance DE, Campenot RB, Vance JE (1997) Role of lipoproteins in the delivery of lipids to axons during axonal regeneration. J Biol Chem 272: 30766–30773. 9388216 10.1074/jbc.272.49.30766

[30] Salzer JL (2003) Polarized domains of myelinated axons. Neuron 40: 297–318. 14556710 10.1016/s0896-6273(03)00628-7

[31] Vincent AM, Hayes JM, McLean LL, Vivekanandan-Giri A, Pennathur S, et al (2009) Dyslipidemia-induced neuropathy in mice: the role of oxLDL/LOX-1. Diabetes 58: 2376–2385. 19592619 10.2337/db09-0047 PMC2750230

[32] Shefner JM, Reaume AG, Flood DG, Scott RW, Kowall NW, et al (1999) Mice lacking cytosolic copper/zinc superoxide dismutase display a distinctive motor axonopathy. Neurology 53: 1239–1246. 10522879 10.1212/wnl.53.6.1239

[33] Fischer LR, Li Y, Asress SA, Jones DP, Glass JD (2012) Absence of SOD1 leads to oxidative stress in peripheral nerve and causes a progressive distal motor axonopathy. Exp Neurol 233: 163–171. 21963651 10.1016/j.expneurol.2011.09.020 PMC4068963

[34] Verheijen MH, Chrast R, Burrola P, Lemke G (2003) Local regulation of fat metabolism in peripheral nerves. Genes Dev 17: 2450–2464. 14522948 10.1101/gad.1116203 PMC218081

[35] Nave KA, Trapp BD (2008) Axon-glial signaling and the glial support of axon function. Annu Rev Neurosci 31: 535–561. 18558866 10.1146/annurev.neuro.30.051606.094309

[36] Viader A, Sasaki Y, Kim S, Strickland A, Workman CS, et al (2013) Aberrant schwann cell lipid metabolism linked to mitochondrial deficits leads to axon degeneration and neuropathy. Neuron 77: 886–898. 23473319 10.1016/j.neuron.2013.01.012 PMC3594792

[37] Fu Q, Goodrum JF, Hayes C, Hostettler JD, Toews AD, et al (1998) Control of cholesterol biosynthesis in Schwann cells. J Neurochem 71: 549–555. 9681444 10.1046/j.1471-4159.1998.71020549.x

[38] Toews AD, Hostettler J, Barrett C, Morell P (1997) Alterations in gene expression associated with primary demyelination and remyelination in the peripheral nervous system. Neurochem Res 22: 1271–1280. 9342732 10.1023/a:1021941215310

[39] Liu JP, Tang Y, Zhou S, Toh BH, McLean C, et al (2010) Cholesterol involvement in the pathogenesis of neurodegenerative diseases. Mol Cell Neurosci 43: 33–42. 19660552 10.1016/j.mcn.2009.07.013

[40] Fischer LR, Glass JD (2010) Oxidative stress induced by loss of Cu,Zn-superoxide dismutase (SOD1) or superoxide-generating herbicides causes axonal degeneration in mouse DRG cultures. Acta Neuropathol 119: 249–259. 20039174 10.1007/s00401-009-0631-z PMC4334446

[41] Clement AB, Gamerdinger M, Tamboli IY, Lutjohann D, Walter J, et al (2009) Adaptation of neuronal cells to chronic oxidative stress is associated with altered cholesterol and sphingolipid homeostasis and lysosomal function. J Neurochem 111: 669–682. 19712059 10.1111/j.1471-4159.2009.06360.x

[42] Bokov A, Chaudhuri A, Richardson A (2004) The role of oxidative damage and stress in aging. Mech Ageing Dev 125: 811–826. 15541775 10.1016/j.mad.2004.07.009

[43] Parkes TL, Elia AJ, Dickinson D, Hilliker AJ, Phillips JP, et al (1998) Extension of Drosophila lifespan by overexpression of human SOD1 in motorneurons. Nat Genet 19: 171–174. 9620775 10.1038/534

[44] Huang TT, Carlson EJ, Gillespie AM, Shi Y, Epstein CJ (2000) Ubiquitous overexpression of CuZn superoxide dismutase does not extend life span in mice. J Gerontol A Biol Sci Med Sci 55: B5–9. 10719757 10.1093/gerona/55.1.b5

[45] Bjelakovic G, Nikolova D, Gluud LL, Simonetti RG, Gluud C (2007) Mortality in randomized trials of antioxidant supplements for primary and secondary prevention: systematic review and meta-analysis. JAMA 297: 842–857. 17327526 10.1001/jama.297.8.842

[46] Hoke A, Redett R, Hameed H, Jari R, Zhou C, et al (2006) Schwann cells express motor and sensory phenotypes that regulate axon regeneration. J Neurosci 26: 9646–9655. 16988035 10.1523/JNEUROSCI.1620-06.2006 PMC6674436

