# Supplementary figures and images for: The Role of Oxidative Stress in Nervous System Aging
Source: PLoS One. 2013 Jul 2;8(7):e68011. doi: 10.1371/journal.pone.0068011 (PMC3699525; doi:10.1371/journal.pone.0068011)

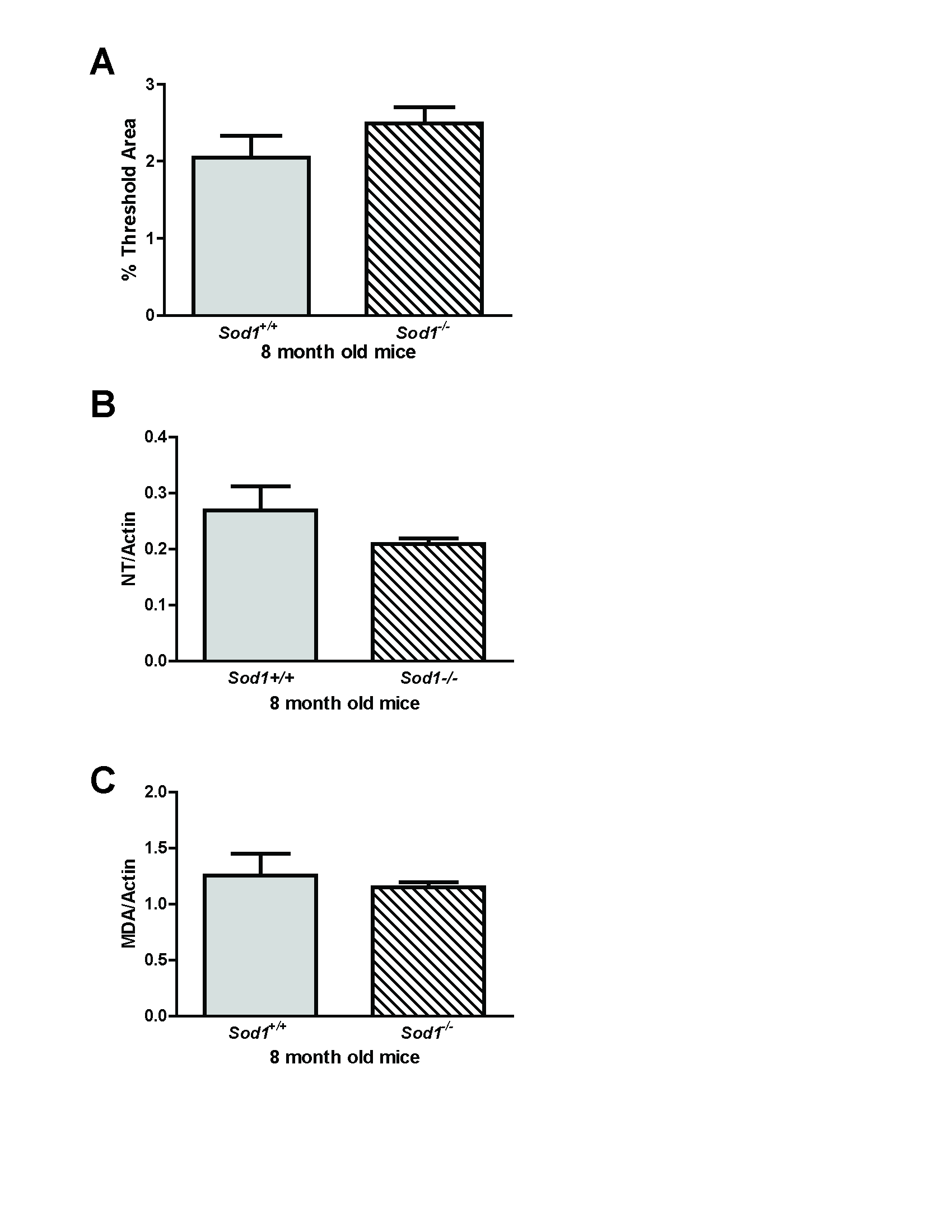

Supplement: Figure S1 — Assessment of oxidative damage in the cell body of sensory neurons in young Sod1 mice. Oxidative damage was assessed in dorsal root ganglia neurons of 8 mo Sod1 mice by A) quantitatively assessing the autofluorescence of lipofuscin and western immunoblotting, and densitometry analysis of B) nitrated proteins (nitrotyrosine, NT) and C) oxidative lipid degradation (malondialdehyde, MDA). Sod1+/+ and Sod1−/− mice are represented by light gray and black/white strip bars, respectively; n≥4. (TIFF) [file pone.0068011.s001.tiff]

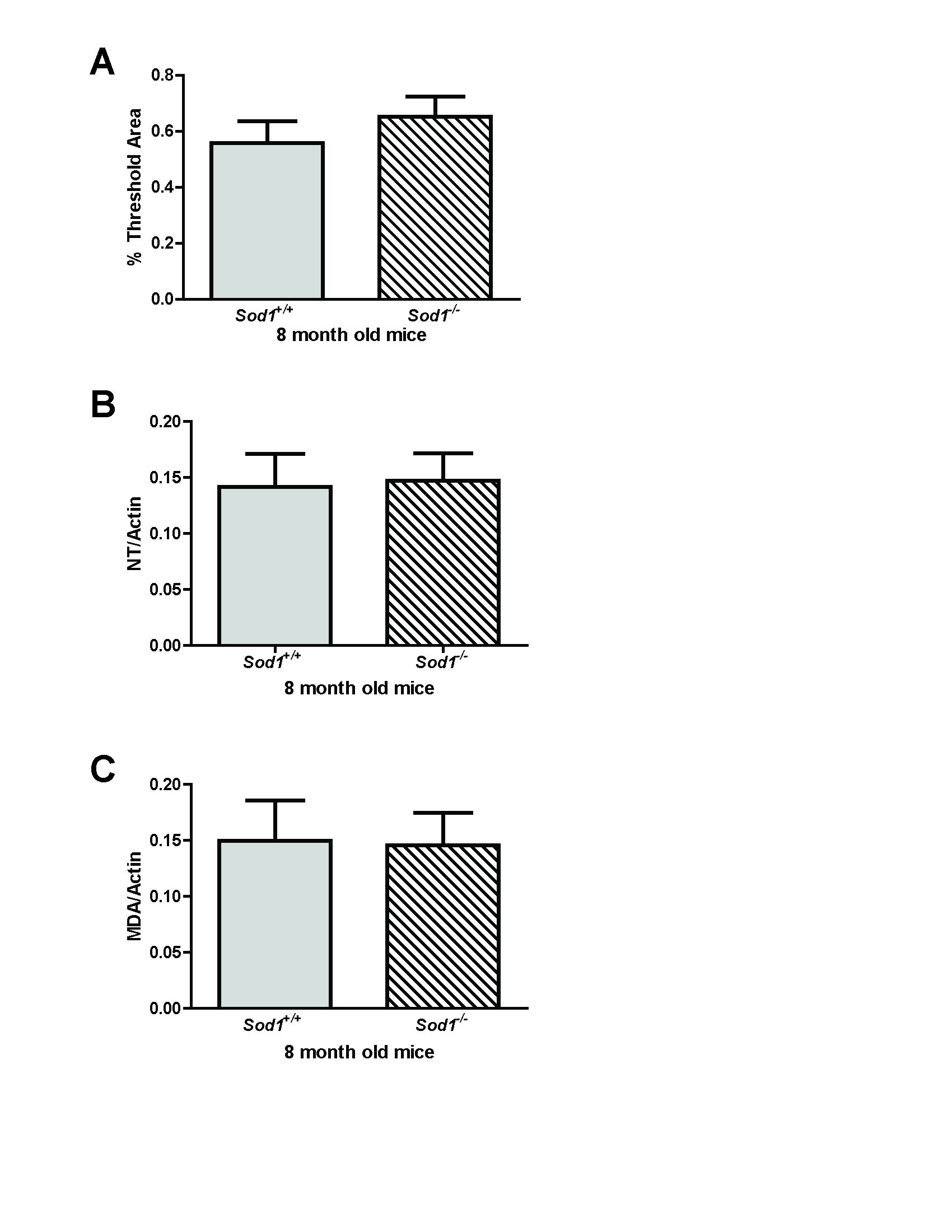

Supplement: Figure S2 — Assessment of oxidative damage in the motor neuron micro-environment in young Sod1 mice. Oxidative damage was assessed in the spinal cord of in 8 mo Sod1 mice by A) quantitatively assessing the autofluorescence of lipofuscin and western immunoblotting, and densitometry analysis of B) nitrated proteins (NT) and C) oxidative lipid degradation (MDA). Sod1+/+ and Sod1−/− mice are represented by light gray and black/white strip bars, respectively; n≥4. (TIFF) [file pone.0068011.s002.tiff]
