# Supplementary material for: The Role of Oxidative Stress in Nervous System Aging
Source: PLoS One. 2013 Jul 2;8(7):e68011. doi: 10.1371/journal.pone.0068011 (PMC3699525; doi:10.1371/journal.pone.0068011)
Supplement: Table S1 — List of DEGs. Fold-changes are given if the gene is a DEG. DEG/Set count indicates the number of DEG sets (out of 6 sets in total) in which the corresponding gene has been identified as a DEG. The total number of DEGs in each set is denoted in red. The two columns highlighted in yellow are the primary DEG sets used in the present study. Any DEGs that were also identified in the cholesterol- and aging-related literature are noted in the last three columns. #Paper: the number of papers in which the corresponding gene was identified by SciMiner; Enrichment: the fold-enrichment of the frequency (the number of papers with the corresponding gene/total number of cholesterol- and aging-related literature) compared to the frequency of the gene in the whole PubMed abstracts; BH P-value: Benjamini-Hochberg corrected p-value of the Fisher’s exact test to test the significance of the gene in the cholesterol- and aging-related literature. The genes are sorted by ‘DEG count’, and the fold-changes in the ‘2mKO_20mKO’ and ‘20mWT_30mWT’ sets. (PDF) [file pone.0068011.s003.pdf]

Gene ID	Symbol	Description	DEG/Set count	DEG Fold-Change: 2mWT_2mKO	20mWT_20mKO	2mKO_20mKO	2mWT_20mWT	2mWT_30mWT	20mWT_30mWT	Literature-Mining (Aging-Cholesterol literature): #Paper	Enrichment	BH P-value
				9	230	406	48	1904	1157	#Paper	Enrichment	BH P-value
14125	Fcer1a	Fc receptor, IgE, high affinity I, alpha polypeptide	4		3.44	2.43		4.54	3.20			
73690	Glipr1	GLI pathogenesis-related 1 (glioma)	4		2.04	2.17		7.33	6.33			
217304	Cd300lb	CD300 antigen like family member B	4		1.91	2.09		4.08	4.06			
14528	Gch1	GTP cyclohydrolase 1	4			1.98	1.70	2.66	1.57			
15442	Hpse	heparanase	4		1.40	1.98		5.24	4.71			
56193	Plek	pleckstrin	4		1.68	1.90		7.26	5.33			
27384	Akr1c13	aldo-keto reductase family 1, member C13	4		1.63	1.84		2.12	1.69			
23890	Gpr34	G protein-coupled receptor 34	4		1.77	1.83		3.38	2.81			
217303	Cd300a	CD300A antigen	4		1.79	1.78		2.94	3.02			
13058	Cybb	cytochrome b-245, beta polypeptide	4		1.64	1.76		5.67	4.65	2	4.8	0.13
20321	Frrs1	ferric-chelate reductase 1	4		1.70	1.76		2.48	3.16			
18173	Slc11a1	solute carrier family 11 (proton-coupled divalent metal ion transporters), member 1	4		1.54	1.71		3.28	2.88			
107321	Lpxn	leupaxin	4		1.67	1.70		7.15	5.81			
11520	Plin2	perilipin 2	4		1.51	1.62		1.56	1.49			
19734	Rgs16	regulator of G-protein signaling 16	4		1.50	1.59		1.94	1.66			
15900	Irf8	interferon regulatory factor 8	4		1.52	1.58		3.90	3.69			
75345	Slamf7	SLAM family member 7	4		1.65	1.54		3.55	4.21			
13057	Cyba	cytochrome b-245, alpha polypeptide	4		1.31	1.54		2.26	1.94	4	15.1	1.15E-03
52855	Lair1	leukocyte-associated Ig-like receptor 1	4		1.66	1.53		4.14	3.32			
13025	Ctla2b	cytotoxic T lymphocyte-associated protein 2 beta	4		1.73	1.49		2.09	2.23			
100504230	LOC100504230	uncharacterized LOC100504230	4		1.31	1.42		2.81	2.71			
13051	Cx3cr1	chemokine (C-X3-C) receptor 1	4		1.38	1.41		2.00	1.80	1	8.2	0.19
227620	Uap1l1	UDP-N-acteylglucosamine pyrophosphorylase 1-like 1	4		1.34	1.28		1.40	1.25			
208748	Prrg3	proline rich Gla (G-carboxyglutamic acid) 3 (transmembrane)	4			-1.29	-1.30	-1.73	-1.33			
235293	Sc5d	sterol-C5-desaturase (fungal ERG3, delta-5-desaturase) homolog (S. cerevisae)	4			-1.38	-1.53	-2.31	-1.51			
105785	Kdelr3	KDEL (Lys-Asp-Glu-Leu) endoplasmic reticulum protein retention receptor 3	4			-1.38	-1.49	-2.00	-1.34			
16835	Ldlr	low density lipoprotein receptor	4			-1.42	-1.26	-1.75	-1.38	97	48.9	1.30E-122
16948	Lox	lysyl oxidase	4			-1.43	-1.42	-3.07	-2.17			
107993	Bfsp2	beaded filament structural protein 2, phakinin	4		-1.69	-1.54		-2.69	-2.15			
74754	Dhcr24	24-dehydrocholesterol reductase	4			-1.79	-1.76	-2.77	-1.58	5	246.1	5.52E-10
12970	Crygs	crystallin, gamma S	4	-2.40	-3.10	-2.03			-2.41			
16840	Lect1	leukocyte cell derived chemotaxin 1	4			-2.49	-2.76	-5.42	-1.97	2	72.3	2.34E-03
21844	Tiam1	T-cell lymphoma invasion and metastasis 1	4	-1.40	-1.33			-1.49	-1.42			
16071	Igkc	immunoglobulin kappa constant	3			8.17	7.91	15.92				
12722	Clca1	chloride channel calcium activated 1	3			4.10		4.81	2.16			
109225	Ms4a7	membrane-spanning 4-domains, subfamily A, member 7	3		2.14	3.32	2.08					
12483	Cd22	CD22 antigen	3			3.23		15.95	9.71			
12523	Cd84	CD84 antigen	3		2.75	2.99		8.43				
21956	Tnnt2	troponin T2, cardiac	3			2.83		-2.40	-2.65	1	1.1	0.66
100034251	Gm11428	predicted gene 11428	3			2.73		4.62	2.65			
11813	Apoc2	apolipoprotein C-II	3		3.11	2.69		6.78		5	62.9	3.12E-07
57266	Cxcl14	chemokine (C-X-C motif) ligand 14	3		2.23	2.66		7.02				
64929	Scel	sciellin	3			2.60	2.77		-1.84			
12514	Cd68	CD68 antigen	3		1.97	2.59		5.18		2	1.7	0.40
74096	Hvcn1	hydrogen voltage-gated channel 1	3			2.38		3.46	2.96			
11303	Abca1	ATP-binding cassette, sub-family A (ABC1), member 1	3		1.38	2.22	1.82			54	157.4	5.86E-95
11865	Arntl	aryl hydrocarbon receptor nuclear translocator-like	3			2.13		-1.55	-2.04			
12931	Crlf1	cytokine receptor-like factor 1	3			2.07		4.44	2.80			
21923	Tnc	tenascin C	3			2.06		5.20	3.06			
16369	Irs3	insulin receptor substrate 3	3			2.01		-1.81	-1.70			
12223	Btc	betacellulin, epidermal growth factor family member	3		1.88	1.94		5.04				
13040	Ctss	cathepsin S	3		1.54	1.84			2.02	1	10.1	0.17
16854	Lgals3	lectin, galactose binding, soluble 3	3		1.61	1.82		2.54				
15559	Htr2b	5-hydroxytryptamine (serotonin) receptor 2B	3			1.75		5.18	3.21	4	0.6	0.41
74202	Fblim1	filamin binding LIM protein 1	3			1.72		2.59	1.99			
231842	Amz1	archaelysin family metallopeptidase 1	3			1.69		3.49	3.06			
20787	Srebf1	sterol regulatory element binding transcription factor 1	3			1.68		1.41	1.49	7	32.7	4.57E-08
16068	Il18bp	interleukin 18 binding protein	3			1.68		3.83	3.69			
54635	Pdgfc	platelet-derived growth factor, C polypeptide	3			1.64		2.08	1.69			
170743	Tlr7	toll-like receptor 7	3			1.62		4.11	3.51			
68279	Mcoln2	mucolipin 2	3			1.60		4.26	4.65			
234878	BC021891	cDNA sequence BC021891	3			1.57	1.68		-1.57			
12506	Cd48	CD48 antigen	3			1.55		4.15	3.75			
14131	Fcgr3	Fc receptor, IgG, low affinity III	3			1.54		2.94	2.66			
14130	Fcgr2b	Fc receptor, IgG, low affinity IIb	3			1.52		3.36	2.45			
20423	Shh	sonic hedgehog	3			1.52	1.54	1.63		2	3	0.22
12262	C1qc	complement component 1, q subcomponent, C chain	3		1.49	1.51		3.52				
18573	Pde1a	phosphodiesterase 1A, calmodulin-dependent	3			1.50		1.79	1.63			
223433	Fam105a	family with sequence similarity 105, member A	3			1.49		2.94	2.16			
67844	Rab32	RAB32, member RAS oncogene family	3			1.48		2.28	1.77			
246746	Cd300lf	CD300 antigen like family member F	3			1.45		2.01	1.94			
66102	Cxcl16	chemokine (C-X-C motif) ligand 16	3			1.44		3.05	2.57			

Gene ID	Symbol	Description	DEG/Set count	2mWT_2mKO	20mWT_20mKO	2mKO_20mKO	2mWT_20mWT	2mWT_30mWT	20mWT_30mWT	Literature-Mining (Aging-Cholesterol literature)		
				9	230	406	48	1904	1157	#Paper	Enrichment	BH P-value
22177	Tyrobp	TYRO protein tyrosine kinase binding protein	3		1.48	1.44		2.27				
74211	1700017B05Rik	RIKEN cDNA 1700017B05 gene	3			1.42		1.95	2.03			
12362	Casp1	caspase 1	3			1.42		2.43	2.02	1	1	0.67
11810	Apobec1	apolipoprotein B mRNA editing enzyme, catalytic polypeptide 1	3			1.41		2.56	2.10	1	15.7	0.13
18414	Osmr	oncostatin M receptor	3			1.38		1.82	1.44			
16658	Mafb	v-maf musculoaponeurotic fibrosarcoma oncogene family, protein B (avian)	3			1.38		2.72	2.54			
18991	Pou3f1	POU domain, class 3, transcription factor 1	3			1.35		1.56	1.35			
70356	St13	suppression of tumorigenicity 13	3		1.27	1.33		1.24				
14860	Gsta4	glutathione S-transferase, alpha 4	3			1.32		-1.34	-1.50			
21937	Tnfrsf1a	tumor necrosis factor receptor superfamily, member 1a	3			1.31		1.53	1.47	2	1.9	0.35
12661	Chl1	cell adhesion molecule with homology to L1CAM	3			1.29		1.57	1.23			
11855	Arhgap5	Rho GTPase activating protein 5	3			1.22		-1.20	-1.31			
270097	Vat1l	vesicle amine transport protein 1 homolog-like (T. californica)	3			-1.13		-1.30	-1.14			
13199	Ddn	dendrin	3			-1.14		-1.45	-1.27			
243548	Prickle2	prickle homolog 2 (Drosophila)	3			-1.17		-1.46	-1.28			
14175	Fgf4	fibroblast growth factor 4	3			-1.20		-1.77	-1.60			
269116	Nfasc	neurofascin	3			-1.20		-1.49	-1.31			
20741	Spnb1	spectrin beta 1	3			-1.21		-1.58	-1.36			
12843	Col1a2	collagen, type I, alpha 2	3			-1.27	-1.25	-1.21		3	5.8	0.05
66985	Rassf7	Ras association (RalGDS/AF-6) domain family (N-terminal) member 7	3			-1.28		-1.84	-1.35			
237930	Ttll6	tubulin tyrosine ligase-like family, member 6	3			-1.30		-1.58	-1.48			
76574	Mfsd2a	major facilitator superfamily domain containing 2A	3			-1.32		-1.91	-1.51			
72978	Cnih3	cornichon homolog 3 (Drosophila)	3			-1.35	-1.63	-1.68				
230899	Nppa	natriuretic peptide type A	3			-1.36		-1.68	-1.42	3	1.3	0.56
231858	Radil	Ras association and DIL domains	3			-1.36		-2.68	-1.93			
74438	Clvs1	clavesin 1	3		-1.46	-1.38			-1.32			
100689	Spon2	spondin 2, extracellular matrix protein	3			-1.41		-2.59	-1.81			
22402	Wisp1	WNT1 inducible signaling pathway protein 1	3			-1.41	-1.49	-1.61				
22431	Wt1	Wilms tumor 1 homolog	3			-1.43		-3.97	-4.00			
225742	St8sia5	ST8 alpha-N-acetyl-neuraminide alpha-2,8-sialyltransferase 5	3			-1.45		-2.20	-1.48			
12960	Crybb1	crystallin, beta B1	3			-1.45		-2.03	-1.51			
21390	Tbxa2r	thromboxane A2 receptor	3			-1.47		-2.73	-1.59			
50782	Rgs11	regulator of G-protein signaling 11	3			-1.49		-2.19	-1.58			
72315	Ccdc74a	coiled-coil domain containing 74A	3		-1.39	-1.49		-1.71				
15160	Serpind1	serine (or cysteine) peptidase inhibitor, clade D, member 1	3			-1.52		-2.53	-1.82	1	5	0.26
320736	E130203B14Rik	RIKEN cDNA E130203B14 gene	3			-1.54	-1.51		-1.55			
319767	Atp10b	ATPase, class V, type 10B	3			-1.57	-2.52	-3.31				
14137	Fdft1	farnesyl diphosphate farnesyl transferase 1	3			-1.58	-1.39	-1.86		9	94	3.78E-14
407243	Tmem189	transmembrane protein 189	3			-1.59	-1.43	-1.70				
192156	Mvd	mevalonate (diphospho) decarboxylase	3			-1.66	-1.91	-2.33		1	4.4	0.28
66234	Sc4mol	sterol-C4-methyl oxidase-like	3			-1.75		-2.07	-1.53			
15490	Hsd17b7	hydroxysteroid (17-beta) dehydrogenase 7	3			-1.77		-2.18	-1.44	1	5.6	0.24
13121	Cyp51	cytochrome P450, family 51	3			-1.79	-1.67		-1.47			
237558	Gm239	predicted gene 239	3			-1.80	-1.87		-1.96			
20775	Sqle	squalene epoxidase	3			-1.84	-1.49	-2.09		1	16.5	0.12
230459	Cyp2j13	cytochrome P450, family 2, subfamily j, polypeptide 13	3			-2.12	-2.48	-4.27				
12587	Mia1	melanoma inhibitory activity 1	3			-2.15	-2.30	-2.87				
140709	Emid2	EMI domain containing 2	3			-2.24	-2.57	-3.80				
215772	9130014G24Rik	RIKEN cDNA 9130014G24 gene	3			-2.41	-1.63	-1.39				
15163	Hcls1	hematopoietic cell specific Lyn substrate 1	3		1.57			3.62	3.90			
16414	Itgb2	integrin beta 2	3		1.65			4.01	3.87			
105855	Nckap1l	NCK associated protein 1 like	3		1.62			3.68	3.84			
80719	Igsf6	immunoglobulin superfamily, member 6	3		1.65			3.81	3.61			
73656	Ms4a6c	membrane-spanning 4-domains, subfamily A, member 6C	3		1.51			3.84	3.48			
216445	Arhgap9	Rho GTPase activating protein 9	3		1.57			2.69	3.19			
23880	Fyb	FYN binding protein	3		1.71			4.08	3.15			
259277	Klk8	kallikrein related-peptidase 8	3	1.89				3.92	3.06			
226652	Arhgap30	Rho GTPase activating protein 30	3		1.51			2.80	2.94			
74191	P2ry13	purinergic receptor P2Y, G-protein coupled 13	3		1.67			3.14	2.93			
192187	Stab1	stabilin 1	3		1.41			2.43	2.48			
63913	Fam129a	family with sequence similarity 129, member A	3		1.38			2.09	2.10			
17133	Maff	v-maf musculoaponeurotic fibrosarcoma oncogene family, protein F (avian)	3		1.70			1.99	2.06	1	107.5	0.03
170706	Tmem37	transmembrane protein 37	3		1.42			1.70	1.96			
17159	Man2b1	mannosidase 2, alpha B1	3		1.46			1.54	1.79			
59058	Bhlhe22	basic helix-loop-helix family, member e22	3		1.37			1.58	1.58			
16494	Kcna6	potassium voltage-gated channel, shaker-related, subfamily, member 6	3		1.49			1.33	1.31			
20198	S100a4	S100 calcium binding protein A4	3		1.20			1.19	1.28	1	5.6	0.24
12804	Cntfr	ciliary neurotrophic factor receptor	3		-1.31			-1.42	-1.51			
100503650	Gm11721	predicted gene 11721	3	-2.13				-1.59	-1.65			
12424	Cck	cholecystokinin	3		-1.51			-1.52	-1.93	2	0.8	1.03
76747	Dapl1	death associated protein-like 1	3		-1.55			-2.18	-2.08			
12655	Chi3l3	chitinase 3-like 3	2			16.55		14.45				

Gene ID	Symbol	Description	DEG/Set count	2mWT_2mKO	20mWT_20mKO	2mKO_20mKO	2mWT_20mWT	2mWT_30mWT	20mWT_30mWT	Literature-Mining (Aging-Cholesterol literature)		
				9	230	406	48	1904	1157	#Paper	Enrichment	BH P-value
17474	Clec4d	C-type lectin domain family 4, member d	2		3.88	8.63						
17381	Mmp12	matrix metallopeptidase 12	2		3.16	4.62						
83433	Trem2	triggering receptor expressed on myeloid cells 2	2		2.07	3.11						
16411	Itgax	integrin alpha X	2		2.08	2.77				1	2.1	0.45
171285	Havcr2	hepatitis A virus cellular receptor 2	2		2.03	2.72						
67477	Abhd15	abhydrolase domain containing 15	2		2.43	2.42						
15483	Hsd11b1	hydroxysteroid 11-beta dehydrogenase 1	2		3.22	2.41				2	8.6	0.06
93695	Gpnmb	glycoprotein (transmembrane) nmb	2		2.00	2.39						
16197	Il7r	interleukin 7 receptor	2		1.73	2.31						
56644	Clec7a	C-type lectin domain family 7, member a	2		1.97	2.30						
68774	Ms4a6d	membrane-spanning 4-domains, subfamily A, member 6D	2		2.08	2.28						
279572	Tlr13	toll-like receptor 13	2		2.39	2.22						
21928	Tnfaip2	tumor necrosis factor, alpha-induced protein 2	2			2.22		4.65				
71760	Agxt2l1	alanine-glyoxylate aminotransferase 2-like 1	2			2.20			-1.89			
12984	Csf2rb2	colony stimulating factor 2 receptor, beta 2, low-affinity (granulocyte-macrophage)	2			2.16		2.00				
383787	Gm1337	predicted gene 1337	2		2.02	2.09						
15199	Hebp1	heme binding protein 1	2			1.99		2.20				
76408	Abcc3	ATP-binding cassette, sub-family C (CFTR/MRP), member 3	2		1.87	1.91						
232431	Gprc5a	G protein-coupled receptor, family C, group 5, member A	2			1.89			-2.01			
70358	Steap1	six transmembrane epithelial antigen of the prostate 1	2			1.81			-1.92			
69387	Dnajb13	DnaJ (Hsp40) related, subfamily B, member 13	2			1.77		2.64				
12267	C3ar1	complement component 3a receptor 1	2		1.85	1.75						
214547	She	src homology 2 domain-containing transforming protein E	2		1.38	1.74						
20249	Scd1	stearoyl-Coenzyme A desaturase 1	2		1.37	1.69				8	22.8	5.55E-08
12983	Csf2rb	colony stimulating factor 2 receptor, beta, low-affinity (granulocyte-macrophage)	2			1.66		2.63				
20660	Sorl1	sortilin-related receptor, LDLR class A repeats-containing	2			1.66		2.31		1	41.7	0.07
56069	Il17b	interleukin 17B	2			1.64		1.52				
81879	Tfcp2l1	transcription factor CP2-like 1	2			1.62		2.12				
17105	Lyz2	lysozyme 2	2			1.62	1.42					
140497	AF251705	cDNA sequence AF251705	2		1.72	1.62						
12522	Cd83	CD83 antigen	2			1.59		2.11				
22414	Wnt2b	wingless related MMTV integration site 2b	2			1.54			-1.91			
18143	Npas2	neuronal PAS domain protein 2	2			1.49			-1.43			
239691	AU021092	expressed sequence AU021092	2			1.47			-1.77			
11816	Apoe	apolipoprotein E	2			1.44			1.61	268	63.1	0
74365	Lonrf3	LON peptidase N-terminal domain and ring finger 3	2			1.43		1.69				
68591	Mocos	molybdenum cofactor sulfurase	2		1.37	1.43						
239133	Dleu7	deleted in lymphocytic leukemia, 7	2			1.42		-2.76				
50528	Tmprss2	transmembrane protease, serine 2	2			1.42		-1.86				
30794	Pdlim4	PDZ and LIM domain 4	2			1.41		1.36				
381413	Gpr176	G protein-coupled receptor 176	2			1.39		1.61				
27965	Spg21	spastic paraplegia 21 homolog (human)	2			1.34		1.42				
227937	Pkp4	plakophilin 4	2			1.32			-1.34			
21351	Taldo1	transaldolase 1	2		1.33	1.28						
20183	Rxrg	retinoid X receptor gamma	2		1.35	1.28						
20661	Sort1	sortilin 1	2		1.30	1.27				1	102.1	0.04
56456	Actl6a	actin-like 6A	2		1.25	1.27						
433375	Creg1	cellular repressor of E1A-stimulated genes 1	2		1.23	1.26						
17536	Meis2	Meis homeobox 2	2			1.24			-1.39			
55983	Pdzrn3	PDZ domain containing RING finger 3	2			1.23		-1.47				
15212	Hexb	hexosaminidase B	2			1.22			1.49			
13030	Ctsb	cathepsin B	2			1.20			1.45	3	3.5	0.12
59095	Fxyd6	FXYD domain-containing ion transport regulator 6	2			1.13			-1.16			
76893	Lass2	LAG1 homolog, ceramide synthase 2	2			-1.15		-1.45				
494448	Cbx6	chromobox homolog 6	2			-1.17		-1.30				
17528	Mpz	myelin protein zero	2			-1.18		-1.25		1	9.9	0.17
16525	Kcnk1	potassium channel, subfamily K, member 1	2			-1.21		-1.54				
216858	Kctd11	potassium channel tetramerisation domain containing 11	2			-1.22		-1.30				
72927	Hepacam	hepatocyte cell adhesion molecule	2			-1.23		-1.56				
74559	Elovl7	ELOVL family member 7, elongation of long chain fatty acids (yeast)	2			-1.24		-1.43				
195209	Gm22	predicted gene 22	2			-1.24			1.54			
12832	Col5a2	collagen, type V, alpha 2	2			-1.28			1.58			
218121	Mboat1	membrane bound O-acyltransferase domain containing 1	2			-1.29		-1.62				
13360	Dhcr7	7-dehydrocholesterol reductase	2			-1.30		-1.41		10	92.2	1.32E-15
67260	Lass4	LAG1 homolog, ceramide synthase 4	2			-1.32		-1.37				
22239	Ugt8a	UDP galactosyltransferase 8A	2			-1.37		-1.69				
432530	Adcy1	adenylate cyclase 1	2			-1.39			-1.74			
108116	Slco3a1	solute carrier organic anion transporter family, member 3a1	2			-1.40		-1.58				
101497	Plekhg2	pleckstrin homology domain containing, family G (with RhoGef domain) member 2	2			-1.40		-1.69				

Gene ID	Symbol	Description	DEG/Set count	2mWT_2mKO	20mWT_20mKO	2mKO_20mKO	2mWT_20mWT	2mWT_30mWT	20mWT_30mWT	Literature-Mining (Aging-Cholesterol literature)		
				9	230	406	48	1904	1157	#Paper	Enrichment	BH P-value
13116	Cyp46a1	cytochrome P450, family 46, subfamily a, polypeptide 1	2			-1.40		-1.88		15	806.4	1.50E-36
12825	Col3a1	collagen, type III, alpha 1	2			-1.41		-1.85		1	12.1	0.15
215814	Ccdc28a	coiled-coil domain containing 28A	2		-1.41	-1.44						
20356	Sema5a	sema domain, seven thrombospondin repeats (type 1 and type 1-like), transmembrane domain (TM) and short cytoplasmic domain, (semaphorin) 5A	2			-1.45			-1.85			
12818	Col14a1	collagen, type XIV, alpha 1	2			-1.46		-1.75				
207911	Mchr1	melanin-concentrating hormone receptor 1	2			-1.46		-1.72				
13603	Opn3	opsin 3	2			-1.47		-1.75				
52463	Tet1	tet oncogene 1	2			-1.49		-1.43				
22029	Traf1	TNF receptor-associated factor 1	2			-1.54	-1.63					
100503043	LOC100503043	armadillo repeat containing, X-linked 4	2		-1.52	-1.55						
54371	Chst2	carbohydrate sulfotransferase 2	2			-1.55	-1.64					
56066	Cxcl11	chemokine (C-X-C motif) ligand 11	2			-1.55		-1.64				
26456	Sema4g	sema domain, immunoglobulin domain (Ig), transmembrane domain (TM) and short cytoplasmic domain, (semaphorin) 4G	2			-1.57		-1.67				
269344	Ell3	elongation factor RNA polymerase II-like 3	2		-1.83	-1.63						
56741	Igdcc4	immunoglobulin superfamily, DCC subclass, member 4	2		-1.74	-1.66						
66355	Gmpr	guanosine monophosphate reductase	2		-1.80	-1.66						
170459	Stard4	StAR-related lipid transfer (START) domain containing 4	2			-1.69		-2.32		1	240.3	0.02
14401	Gabrb2	gamma-aminobutyric acid (GABA) A receptor, subunit beta 2	2		-2.15	-1.69						
231293	Cwh43	cell wall biogenesis 43 C-terminal homolog (S. cerevisiae)	2			-1.76		1.64				
13170	Dbp	D site albumin promoter binding protein	2			-1.88			2.99	1	3.4	0.32
101883	Tmem149	transmembrane protein 149	2			-2.01		-2.27				
74770	Hhatl	hedgehog acyltransferase-like	2		-2.47	-2.08						
238988	Erc2	ELKS/RAB6-interacting/CAST family member 2	2		-2.12	-2.08						
227394	Slco4c1	solute carrier organic anion transporter family, member 4C1	2			-2.10	-2.92					
73166	Tm7sf2	transmembrane 7 superfamily member 2	2			-2.10		-1.70				
269784	Cntn4	contactin 4	2		-2.33	-2.19						
230235	6430704M03Rik	RIKEN cDNA 6430704M03 gene	2		-2.21	-2.42						
229599	Gm129	predicted gene 129	2			-2.48			2.40			
329217	Gm5101	predicted gene 5101	2			-2.50	-3.04					
114644	Slc13a3	solute carrier family 13 (sodium-dependent dicarboxylate transporter), member 3	2		-2.02	-2.53				1	157.1	0.03
100503019	Gm16551	predicted gene 16551	2		-3.17	-3.50						
19215	Ptgds	prostaglandin D2 synthase (brain)	2		-3.99	-5.42						
11656	Alas2	aminolevulinic acid synthase 2, erythroid	2					29.00	24.18			
328563	Apol11b	apolipoprotein L 11b	2					16.87	21.75			
56620	Clec4n	C-type lectin domain family 4, member n	2					13.93	9.41			
57349	Ppbp	pro-platelet basic protein	2					8.77	9.08	5	7.8	3.18E-03
433637	Gm5547	predicted gene 5547	2					10.43	8.76			
20210	Saa3	serum amyloid A 3	2					12.19	8.13	1	15.3	0.13
229499	Fcrl1	Fc receptor-like 1	2					7.79	7.71			
16175	Il1a	interleukin 1 alpha	2					7.62	7.36	6	1.1	0.70
12502	Cd3g	CD3 antigen, gamma polypeptide	2					9.16	7.01			
12493	Cd37	CD37 antigen	2					7.34	6.69			
622976	Gm6377	predicted gene 6377	2					6.94	6.61			
17972	Ncf4	neutrophil cytosolic factor 4	2					6.50	6.28			
50778	Rgs1	regulator of G-protein signaling 1	2					8.07	6.25			
17079	Cd180	CD180 antigen	2					6.18	5.84			
16019	Ighm	immunoglobulin heavy constant mu	2					15.85	5.54			
94176	Dock2	dedicator of cyto-kinesis 2	2					5.92	5.53			
18140	Uhrf1	ubiquitin-like, containing PHD and RING finger domains, 1	2					5.44	5.52			
433470	AA467197	expressed sequence AA467197	2					5.69	5.46			
56760	Clec1b	C-type lectin domain family 1, member b	2					6.87	5.25			
20209	Saa2	serum amyloid A 2	2					4.14	5.18			
13041	Ctsw	cathepsin W	2					4.91	4.93			
17060	Blnk	B-cell linker	2					5.68	4.87			
21897	Tlr1	toll-like receptor 1	2					4.70	4.61			
20307	Ccl8	chemokine (C-C motif) ligand 8	2					11.64	4.59			
100043636	AI662270	expressed sequence AI662270	2					4.85	4.54			
242705	E2f2	E2F transcription factor 2	2					5.56	4.49			
12766	Cxcr3	chemokine (C-X-C motif) receptor 3	2					4.63	4.41			
320148	B430306N03Rik	RIKEN cDNA B430306N03 gene	2					5.24	4.36			
12487	Cd28	CD28 antigen	2					3.98	4.28	3	2.3	0.22
72054	Cyp4f18	cytochrome P450, family 4, subfamily f, polypeptide 18	2					3.78	4.22			
74131	Sash3	SAM and SH3 domain containing 3	2					3.81	4.15			
21973	Top2a	topoisomerase (DNA) II alpha	2					3.81	4.03			
14744	Gpr65	G-protein coupled receptor 65	2					5.07	4.01			
15170	Ptpn6	protein tyrosine phosphatase, non-receptor type 6	2					4.49	4.01			
12183	Bpgm	2,3-bisphosphoglycerate mutase	2					4.32	3.98			
15945	Cxcl10	chemokine (C-X-C motif) ligand 10	2					5.14	3.98	2	2.4	0.28
20302	Ccl3	chemokine (C-C motif) ligand 3	2					4.08	3.98	1	1.3	0.59
72925	March1	membrane-associated ring finger (C3HC4) 1	2					3.85	3.98			

Gene ID	Symbol	Description	DEG/Set count	2mWT_2mKO	20mWT_20mKO	2mKO_20mKO	2mWT_20mWT	2mWT_30mWT	20mWT_30mWT	Literature-Mining (Aging-Cholesterol literature)		
				9	230	406	48	1904	1157	#Paper	Enrichment	BH P-value
246256	Fcgr4	Fc receptor, IgG, low affinity IV	2					3.42	3.97	2	1.8	0.38
23845	Clec5a	C-type lectin domain family 5, member a	2					4.27	3.93			
14017	Evi2a	ecotropic viral integration site 2a	2					3.89	3.92			
268697	Ccnb1	cyclin B1	2					3.03	3.89			
20491	Sla	src-like adaptor	2					3.91	3.89			
16643	Klrd1	killer cell lectin-like receptor, subfamily D, member 1	2					3.97	3.87			
278180	Vsig4	V-set and immunoglobulin domain containing 4	2					5.01	3.84			
209837	Slc38a5	solute carrier family 38, member 5	2					3.58	3.82	1	9.8	0.17
12501	Cd3e	CD3 antigen, epsilon polypeptide	2					3.80	3.81			
23900	Hcst	hematopoietic cell signal transducer	2					3.94	3.77			
56743	Lat2	linker for activation of T cells family, member 2	2					4.53	3.63			
22324	Vav1	vav 1 oncogene	2					3.57	3.62			
20345	Selplg	selectin, platelet (p-selectin) ligand	2					3.27	3.54			
212032	Hk3	hexokinase 3	2					3.07	3.53			
17476	Mpeg1	macrophage expressed gene 1	2		1.91				3.51			
18733	Lilrb3	leukocyte immunoglobulin-like receptor, subfamily B (with TM and ITIM domains), member 3	2					3.79	3.47			
108101	Fermt3	fermitin family homolog 3 (Drosophila)	2					3.10	3.46			
67712	Slc25a37	solute carrier family 25, member 37	2					3.83	3.44			
19264	Ptprc	protein tyrosine phosphatase, receptor type, C	2					4.33	3.42	5	3.1	0.07
76459	Car12	carbonic anyhydrase 12	2					3.30	3.40			
17951	Naip5	NLR family, apoptosis inhibitory protein 5	2					3.52	3.35			
16182	Il18r1	interleukin 18 receptor 1	2					3.03	3.30			
20135	Rrm2	ribonucleotide reductase M2	2					3.19	3.30			
12508	Cd53	CD53 antigen	2					3.60	3.29			
20288	Msr1	macrophage scavenger receptor 1	2					3.21	3.27	2	15.4	0.03
17969	Ncf1	neutrophil cytosolic factor 1	2					3.28	3.24			
17948	Naip2	NLR family, apoptosis inhibitory protein 2	2					4.29	3.23			
52033	Pbk	PDZ binding kinase	2					2.85	3.22			
66610	Abi3	ABI gene family, member 3	2					2.83	3.22			
14799	Gria1	glutamate receptor, ionotropic, AMPA1 (alpha 1)	2					3.51	3.18	1	0.4	0.78
57781	Cd200r1	CD200 receptor 1	2					2.81	3.17			
18557	Cdk18	cyclin-dependent kinase 18	2					3.55	3.16			
192188	Stab2	stabilin 2	2					2.81	3.13			
380732	Gm885	predicted gene 885	2					2.53	3.13			
80891	Fcrls	Fc receptor-like S, scavenger receptor	2					3.16	3.06			
11629	Aif1	allograft inflammatory factor 1	2					2.78	3.01	1	9.5	0.17
69769	Tnfaip8l2	tumor necrosis factor, alpha-induced protein 8-like 2	2					3.27	2.97			
68662	Scgb3a1	secretoglobin, family 3A, member 1	2					3.80	2.96			
12802	Cnr2	cannabinoid receptor 2 (macrophage)	2					2.78	2.95			
17394	Mmp8	matrix metallopeptidase 8	2					2.93	2.93			
20556	Slfn2	schlafen 2	2					3.47	2.91			
80901	Cxcr6	chemokine (C-X-C motif) receptor 6	2					3.44	2.88			
21990	Tph1	tryptophan hydroxylase 1	2		2.10				2.85			
15430	Hoxd10	homeobox D10	2					3.28	2.84			
232201	Arhgap25	Rho GTPase activating protein 25	2					3.32	2.84			
320207	Pik3r5	phosphoinositide-3-kinase, regulatory subunit 5, p101	2					2.95	2.76			
14281	Fos	FBJ osteosarcoma oncogene	2					3.87	2.71	11	1.7	0.15
15902	Id2	inhibitor of DNA binding 2	2					4.63	2.71			
21391	Tbxas1	thromboxane A synthase 1, platelet	2					2.51	2.69	23	4.6	4.61E-08
27052	Aoah	acyloxyacyl hydrolase	2					3.03	2.68			
11846	Arg1	arginase, liver	2					3.02	2.68	1	30.3	0.08
14544	Gda	guanine deaminase	2					3.39	2.61			
11799	Birc5	baculoviral IAP repeat-containing 5	2					3.30	2.61	1	1.1	0.63
223838	Adamts20	a disintegrin-like and metallopeptidase (reprolysin type) with thrombospondin type 1 motif, 20	2					3.18	2.60			
233406	Prc1	protein regulator of cytokinesis 1	2					2.77	2.60			
17085	Ly9	lymphocyte antigen 9	2					2.18	2.58			
24055	Sh3bp2	SH3-domain binding protein 2	2					2.51	2.57			
54445	Unc93b1	unc-93 homolog B1 (C. elegans)	2					2.29	2.56			
20375	Sfpi1	SFFV proviral integration 1	2					2.57	2.55			
23833	Cd52	CD52 antigen	2					2.43	2.54			
217203	Tmem106a	transmembrane protein 106A	2					2.67	2.54			
64214	Rgs18	regulator of G-protein signaling 18	2					2.51	2.53			
20296	Ccl2	chemokine (C-C motif) ligand 2	2					2.37	2.52	14	4.6	3.61E-05
16154	Il10ra	interleukin 10 receptor, alpha	2					2.42	2.52			
15364	Hmga2	high mobility group AT-hook 2	2		1.74				2.51			
13733	Emr1	EGF-like module containing, mucin-like, hormone receptor-like sequence 1	2					2.87	2.51			
67749	4930583H14Rik	RIKEN cDNA 4930583H14 gene	2					3.02	2.49			
19141	Lgmn	legumain	2					3.38	2.45			
18793	Plaur	plasminogen activator, urokinase receptor	2					2.42	2.44			
21418	Tfap2a	transcription factor AP-2, alpha	2					2.98	2.44	2	4	0.16
21826	Thbs2	thrombospondin 2	2					2.02	2.44			
12767	Cxcr4	chemokine (C-X-C motif) receptor 4	2					2.39	2.42	4	3.5	0.07
29873	Cspg5	chondroitin sulfate proteoglycan 5	2					2.09	2.42			
233571	P2ry6	pyrimidinergic receptor P2Y, G-protein coupled, 6	2					1.90	2.37			
13036	Ctsh	cathepsin H	2					2.07	2.37			

Gene ID	Symbol	Description	DEG/Set count	2mWT_2mKO	20mWT_20mKO	2mKO_20mKO	2mWT_20mWT	2mWT_30mWT	20mWT_30mWT	Literature-Mining (Aging-Cholesterol literature)		
				9	230	406	48	1904	1157	#Paper	Enrichment	BH P-value
72119	Tpx2	TPX2, microtubule-associated protein homolog (Xenopus laevis)	2					2.01	2.37			
24088	Tlr2	toll-like receptor 2	2					2.76	2.33	1	1	1.04
20306	Ccl7	chemokine (C-C motif) ligand 7	2					2.39	2.32			
239743	Klhl6	kelch-like 6 (Drosophila)	2					2.42	2.32			
20305	Ccl6	chemokine (C-C motif) ligand 6	2					2.25	2.31			
20308	Ccl9	chemokine (C-C motif) ligand 9	2					2.11	2.31			
14129	Fcgr1	Fc receptor, IgG, high affinity I	2					2.38	2.31			
12051	Bcl3	B-cell leukemia/lymphoma 3	2					2.31	2.29			
54486	Hpgds	hematopoietic prostaglandin D synthase	2					2.82	2.29			
71398	5430427O19Rik	RIKEN cDNA 5430427O19 gene	2					3.08	2.27			
21803	Tgfb1	transforming growth factor, beta 1	2					2.22	2.24	12	1.4	0.31
226101	Myof	myoferlin	2					2.18	2.23			
18751	Prkcb	protein kinase C, beta	2					3.09	2.23			
23948	Mmp17	matrix metallopeptidase 17	2					1.94	2.20			
76438	Rftn1	raftlin lipid raft linker 1	2					1.99	2.19			
53945	Slc40a1	solute carrier family 40 (iron-regulated transporter), member 1	2					2.03	2.19			
11513	Adcy7	adenylate cyclase 7	2					2.18	2.18	3	1.5	0.51
27056	Irf5	interferon regulatory factor 5	2					2.10	2.17			
228482	Arhgap11a	Rho GTPase activating protein 11A	2					2.29	2.17			
16331	Inpp5d	inositol polyphosphate-5-phosphatase D	2					2.21	2.16			
19217	Ptger2	prostaglandin E receptor 2 (subtype EP2)	2					2.19	2.15			
12986	Csf3r	colony stimulating factor 3 receptor (granulocyte)	2					2.11	2.15			
21816	Tgm1	transglutaminase 1, K polypeptide	2					2.31	2.14	1	5.4	0.24
404710	Iqgap3	IQ motif containing GTPase activating protein 3	2					2.63	2.12			
72318	Cyth4	cytohesin 4	2					2.33	2.12			
18768	Pkib	protein kinase inhibitor beta, cAMP dependent, testis specific	2					2.62	2.10			
64136	Sdf2l1	stromal cell-derived factor 2-like 1	2					2.82	2.09			
74490	Mamstr	MEF2 activating motif and SAP domain containing transcriptional regulator	2					1.71	2.09			
106869	Tnfaip8	tumor necrosis factor, alpha-induced protein 8	2					2.43	2.08			
26360	Angptl2	angiopoietin-like 2	2					1.86	2.08			
16822	Lcp2	lymphocyte cytosolic protein 2	2					2.02	2.08			
67220	Plekho1	pleckstrin homology domain containing, family O member 1	2					1.95	2.06			
329679	Fnip2	folliculin interacting protein 2	2					2.16	2.06			
13024	Ctla2a	cytotoxic T lymphocyte-associated protein 2 alpha	2					2.01	2.04			
20698	Sphk1	sphingosine kinase 1	2					1.88	2.04			
50917	Galns	galactosamine (N-acetyl)-6-sulfate sulfatase	2					1.71	2.04	1	2.1	0.45
19296	Pvt1	plasmacytoma variant translocation 1	2					2.30	2.03			
77209	8030453O22Rik	RIKEN cDNA 8030453O22 gene	2					2.48	2.03			
213068	Tmem71	transmembrane protein 71	2					2.30	2.02			
72042	Cotl1	coactosin-like 1 (Dictyostelium)	2					1.72	2.01			
11910	Atf3	activating transcription factor 3	2					1.46	1.98			
211550	Tifa	TRAF-interacting protein with forkhead-associated domain	2					2.17	1.97			
213696	Duoxa1	dual oxidase maturation factor 1	2					2.01	1.96			
574428	Zmynd15	zinc finger, MYND-type containing 15	2					2.14	1.96			
20674	Sox2	SRY-box containing gene 2	2					2.72	1.94			
216991	Adap2	ArfGAP with dual PH domains 2	2					1.59	1.94			
73112	3110003A17Rik	RIKEN cDNA 3110003A17 gene	2					2.05	1.94			
108911	Rcc2	regulator of chromosome condensation 2	2					2.38	1.94			
12971	Crym	crystallin, mu	2					2.08	1.93			
17304	Mfge8	milk fat globule-EGF factor 8 protein	2					1.76	1.93			
20652	Soat1	sterol O-acyltransferase 1	2					2.21	1.92	15	154	1.92E-26
16913	Psmb8	proteasome (prosome, macropain) subunit, beta type 8 (large multifunctional peptidase 7)	2					1.97	1.91			
94224	Srd5a2	steroid 5 alpha-reductase 2	2					2.24	1.91			
277360	Prex1	phosphatidylinositol-3,4,5-trisphosphate-dependent Rac exchange factor 1	2					2.18	1.90			
14056	Ezh2	enhancer of zeste homolog 2 (Drosophila)	2		1.47				1.89			
226841	Vash2	vasohibin 2	2					1.81	1.88			
80982	9930013L23Rik	RIKEN cDNA 9930013L23 gene	2					1.91	1.87			
56470	Rgs19	regulator of G-protein signaling 19	2					2.04	1.87			
16978	Lrrfip1	leucine rich repeat (in FLII) interacting protein 1	2					1.99	1.87			
73230	Bmper	BMP-binding endothelial regulator	2					2.25	1.85			
12363	Casp4	caspase 4, apoptosis-related cysteine peptidase	2					2.12	1.84			
74732	Stx11	syntaxin 11	2					2.08	1.84			
57778	Fmnl1	formin-like 1	2					2.11	1.83			
14537	Gcnt1	glucosaminyl (N-acetyl) transferase 1, core 2	2					1.62	1.83			
270198	Pfkfb4	6-phosphofructo-2-kinase/fructose-2,6-biphosphatase 4	2					1.87	1.83			
57914	Crlf2	cytokine receptor-like factor 2	2					2.38	1.82			
229694	AI504432	expressed sequence AI504432	2					1.94	1.82			
57441	Gmnn	geminin	2					1.86	1.81			
110006	Gusb	glucuronidase, beta	2					1.85	1.80			
71706	Slc46a3	solute carrier family 46, member 3	2					1.74	1.80			
20292	Ccl11	chemokine (C-C motif) ligand 11	2					1.62	1.79			
12642	Ch25h	cholesterol 25-hydroxylase	2					1.70	1.79			
20677	Sox4	SRY-box containing gene 4	2					1.90	1.78			

Gene ID	Symbol	Description	DEG/Set count	2mWT_2mKO	20mWT_20mKO	2mKO_20mKO	2mWT_20mWT	2mWT_30mWT	20mWT_30mWT	Literature-Mining (Aging-Cholesterol literature)		
				9	230	406	48	1904	1157	#Paper	Enrichment	BH P-value
14469	Gbp2	guanylate binding protein 2	2					1.85	1.77			
20878	Aurka	aurora kinase A	2					1.63	1.77			
209760	Tmc7	transmembrane channel-like gene family 7	2					2.12	1.77			
16565	Kif21b	kinesin family member 21B	2					1.69	1.76			
14151	Fech	ferrochelatase	2					1.81	1.76	1	5.3	0.24
16426	Itih3	inter-alpha trypsin inhibitor, heavy chain 3	2					2.48	1.76			
320587	Tmem88b	transmembrane protein 88B	2					1.65	1.76			
74568	Mlkl	mixed lineage kinase domain-like	2					1.86	1.75			
12702	Socs3	suppressor of cytokine signaling 3	2					1.80	1.75	3	8.8	0.02
20911	Stxbp2	syntaxin binding protein 2	2		1.50				1.75			
14187	Akr1b8	aldo-keto reductase family 1, member B8	2					1.81	1.74			
12505	Cd44	CD44 antigen	2					2.54	1.73	3	1.4	0.54
212398	Frat2	frequently rearranged in advanced T-cell lymphomas 2	2					1.55	1.73			
74637	Shpk	sedoheptulokinase	2					1.67	1.72			
13803	Enc1	ectodermal-neural cortex 1	2					1.73	1.72			
217826	Kcnk13	potassium channel, subfamily K, member 13	2					1.42	1.71			
22350	Ezr	ezrin	2					1.96	1.69			
233979	Tpcn2	two pore segment channel 2	2					1.57	1.69			
27060	Tcirg1	T-cell, immune regulator 1, ATPase, H+ transporting, lysosomal V0 protein A3	2					1.67	1.68			
71436	Flrt3	fibronectin leucine rich transmembrane protein 3	2					2.36	1.68			
73649	Cybrd1	cytochrome b reductase 1	2					1.59	1.68			
109901	Cela1	chymotrypsin-like elastase family, member 1	2					2.56	1.67			
64540	Tspan4	tetraspanin 4	2					1.55	1.67			
215748	Cnksr3	Cnksr family member 3	2					1.57	1.67			
108150	Galnt7	UDP-N-acetyl-alpha-D-galactosamine: polypeptide N-acetylgalactosaminyltransferase 7	2					1.90	1.67			
18081	Ninj1	ninjurin 1	2					1.82	1.67			
93692	Glrx	glutaredoxin	2					2.56	1.67	1	5.7	0.23
210094	Iglon5	IgLON family member 5	2					2.10	1.67			
11830	Aqp5	aquaporin 5	2					1.76	1.67			
107581	Col16a1	collagen, type XVI, alpha 1	2					1.56	1.66			
226421	5430435G22Rik	RIKEN cDNA 5430435G22 gene	2					1.38	1.66			
69069	1810011H11Rik	RIKEN cDNA 1810011H11 gene	2					1.58	1.66			
27279	Tnfrsf12a	tumor necrosis factor receptor superfamily, member 12a	2					2.25	1.66			
12018	Bak1	BCL2-antagonist/killer 1	2					2.05	1.66			
13618	Ednrb	endothelin receptor type B	2					1.60	1.66			
71797	Chst13	carbohydrate (chondroitin 4) sulfotransferase 13	2					2.01	1.65			
80859	Nfkbiz	nuclear factor of kappa light polypeptide gene enhancer in B-cells inhibitor, zeta	2					2.02	1.65			
69309	Slc16a13	solute carrier family 16 (monocarboxylic acid transporters), member 13	2				-1.52		1.64			
19092	Prkg2	protein kinase, cGMP-dependent, type II	2					2.31	1.63			
384198	Fam47e	family with sequence similarity 47, member E	2	-1.87					1.62			
16513	Kcnj10	potassium inwardly-rectifying channel, subfamily J, member 10	2					2.37	1.61			
12273	C5ar1	complement component 5a receptor 1	2					1.67	1.61			
320840	Negr1	neuronal growth regulator 1	2					1.54	1.61			
23871	Ets1	E26 avian leukemia oncogene 1, 5' domain	2					1.98	1.60			
12125	Bcl2l11	BCL2-like 11 (apoptosis facilitator)	2					1.55	1.60			
104681	Slc16a6	solute carrier family 16 (monocarboxylic acid transporters), member 6	2					1.97	1.60			
12091	Glb1	galactosidase, beta 1	2		1.24				1.60			
71862	Gpr160	G protein-coupled receptor 160	2					1.42	1.60			
235283	Gramd1b	GRAM domain containing 1B	2					1.87	1.59			
16164	Il13ra1	interleukin 13 receptor, alpha 1	2					1.70	1.59			
12260	C1qb	complement component 1, q subcomponent, beta polypeptide	2					1.54	1.58			
69976	Galk2	galactokinase 2	2					1.40	1.57			
12443	Ccnd1	cyclin D1	2					1.76	1.57	1	0.4	0.77
20893	Bhlhe40	basic helix-loop-helix family, member e40	2					1.38	1.57			
68713	Ifitm1	interferon induced transmembrane protein 1	2					1.59	1.57			
16889	Lipa	lysosomal acid lipase A	2					1.49	1.57	8	49.2	1.95E-10
17768	Mthfd2	methylenetetrahydrofolate dehydrogenase (NAD+ dependent), methenyltetrahydrofolate cyclohydrolase	2					1.57	1.56			
54353	Skap2	src family associated phosphoprotein 2	2					1.66	1.55			
27756	Lsm2	LSM2 homolog, U6 small nuclear RNA associated (S. cerevisiae)	2					1.66	1.55			
17750	Mt2	metallothionein 2	2					1.72	1.55			
13175	Dclk1	doublecortin-like kinase 1	2					1.78	1.55			
69550	Bst2	bone marrow stromal cell antigen 2	2					1.99	1.54			
330812	Rnf150	ring finger protein 150	2					1.88	1.54			
23965	Odz3	odd Oz/ten-m homolog 3 (Drosophila)	2					1.52	1.54			
192654	Pla2g15	phospholipase A2, group XV	2					1.60	1.54			
67876	Coq10b	coenzyme Q10 homolog B (S. cerevisiae)	2					1.67	1.53			
20397	Sgpl1	sphingosine phosphate lyase 1	2					1.59	1.53			
100043272	5430417L22Rik	RIKEN cDNA 5430417L22 gene	2					1.90	1.52			
18301	Fxyd5	FXYD domain-containing ion transport regulator 5	2					1.67	1.51			
11568	Aebp1	AE binding protein 1	2					1.57	1.51			
244864	Layn	layilin	2					1.49	1.51			

Gene ID	Symbol	Description	DEG/Set count	2mWT_2mKO	20mWT_20mKO	2mKO_20mKO	2mWT_20mWT	2mWT_30mWT	20mWT_30mWT	Literature-Mining (Aging-Cholesterol literature)		
				9	230	406	48	1904	1157	#Paper	Enrichment	BH P-value
14824	Grn	granulin	2					1.33	1.50			
574403	Fam196b	family with sequence similarity 196, member B	2					1.87	1.50			
239027	Arhgap22	Rho GTPase activating protein 22	2					1.46	1.49			
22287	Scgb1a1	secretoglobin, family 1A, member 1 (uteroglobin)	2					1.53	1.49			
67702	Rnf149	ring finger protein 149	2					1.89	1.49			
68089	Arpc4	actin related protein 2/3 complex, subunit 4	2					2.03	1.48			
20716	Serpina3n	serine (or cysteine) peptidase inhibitor, clade A, member 3N	2					1.84	1.48	2	4.3	0.15
433256	Acsl5	acyl-CoA synthetase long-chain family member 5	2					1.54	1.48			
100504422		NA	2					1.53	1.48			
67168	Lpar6	lysophosphatidic acid receptor 6	2					1.45	1.47			
16477	Junb	Jun-B oncogene	2					1.87	1.47			
212555	Pqlc2	PQ loop repeat containing 2	2					1.47	1.47			
12509	Cd59a	CD59a antigen	2					1.33	1.46			
101476	Plekha1	pleckstrin homology domain containing, family A (phosphoinositide binding specific) member 1	2					1.64	1.46			
219140	Spata13	spermatogenesis associated 13	2					1.53	1.45			
57784	Bin3	bridging integrator 3	2					1.45	1.45			
74182	Gpcpd1	glycerophosphocholine phosphodiesterase GDE1 homolog (S. cerevisiae)	2					1.60	1.45			
373864	Col27a1	collagen, type XXVII, alpha 1	2					1.48	1.45			
20908	Stx3	syntaxin 3	2					1.58	1.45			
76448	2310014H01Rik	protein phosphatase 1, regulatory subunit 18	2		1.30				1.45			
20349	Sema3e	sema domain, immunoglobulin domain (Ig), short basic domain, secreted, (semaphorin) 3E	2					1.61	1.45			
18438	P2rx4	purinergic receptor P2X, ligand-gated ion channel 4	2					1.59	1.44			
69399	1700025G04Rik	RIKEN cDNA 1700025G04 gene	2					1.42	1.44			
72293	Nkd2	naked cuticle 2 homolog (Drosophila)	2					1.44	1.43			
20017	Polr1b	polymerase (RNA) I polypeptide B	2		1.33				1.43			
218203	Mylip	myosin regulatory light chain interacting protein	2					1.92	1.43			
14766	Gpr56	G protein-coupled receptor 56	2					1.92	1.42			
231871	Daglb	diacylglycerol lipase, beta	2					1.40	1.42			
80752	Fam20c	family with sequence similarity 20, member C	2					1.29	1.42	1	14.1	0.14
67223	Rrp15	ribosomal RNA processing 15 homolog (S. cerevisiae)	2					1.77	1.42			
16188	Il3ra	interleukin 3 receptor, alpha chain	2		1.43				1.41			
23790	Coro1c	coronin, actin binding protein 1C	2					1.75	1.41			
66950	Tmem206	transmembrane protein 206	2					1.62	1.40			
16952	Anxa1	annexin A1	2					1.64	1.40			
21346	Tagln2	transgelin 2	2				1.28		1.40			
52552	Parp8	poly (ADP-ribose) polymerase family, member 8	2					1.55	1.40			
19171	Psmb10	proteasome (prosome, macropain) subunit, beta type 10	2					1.51	1.40			
14284	Fosl2	fos-like antigen 2	2					1.37	1.40			
216742	Fnip1	folliculin interacting protein 1	2					1.66	1.40			
64138	Ctsz	cathepsin Z	2					1.48	1.40			
72640	Mex3a	mex3 homolog A (C. elegans)	2					1.46	1.40			
58194	Sh3kbp1	SH3-domain kinase binding protein 1	2					1.62	1.40			
13008	Csrp2	cysteine and glycine-rich protein 2	2					1.53	1.39			
67603	Dusp6	dual specificity phosphatase 6	2					1.82	1.38			
16558	Kif16b	kinesin family member 16B	2					1.62	1.38			
66042	Sostdc1	sclerostin domain containing 1	2					1.43	1.37			
11307	Abcg1	ATP-binding cassette, sub-family G (WHITE), member 1	2					1.68	1.37	14	161.6	5.50E-25
11605	Gla	galactosidase, alpha	2					1.46	1.36	3	5.7	0.05
66249	Pno1	partner of NOB1 homolog (S. cerevisiae)	2					1.57	1.36			
330836	Slc7a6	solute carrier family 7 (cationic amino acid transporter, y+ system), member 6	2					1.41	1.35			
15211	Hexa	hexosaminidase A	2					1.37	1.35			
109778	Blvra	biliverdin reductase A	2					1.34	1.34	1	45.9	0.06
547253	Parp14	poly (ADP-ribose) polymerase family, member 14	2					1.35	1.34			
107702	Rnh1	ribonuclease/angiogenin inhibitor 1	2					1.36	1.33			
218454	Lhfpl2	lipoma HMGIC fusion partner-like 2	2					1.45	1.33			
13732	Emp3	epithelial membrane protein 3	2					1.39	1.33			
83768	Dpp7	dipeptidylpeptidase 7	2					1.47	1.32			
54135	Lsr	lipolysis stimulated lipoprotein receptor	2					1.52	1.32	1	77.1	0.04
21887	Tle3	transducin-like enhancer of split 3, homolog of Drosophila E(spl)	2					1.38	1.32			
216869	Arrb2	arrestin, beta 2	2					1.31	1.32			
66912	Bzw2	basic leucine zipper and W2 domains 2	2					1.32	1.32			
103711	Pnpo	pyridoxine 5'-phosphate oxidase	2					1.37	1.31			
14457	Gas7	growth arrest specific 7	2					1.28	1.31			
239857	Cadm2	cell adhesion molecule 2	2					1.54	1.31			
192192	Shkbp1	Sh3kbp1 binding protein 1	2					1.35	1.30			
19156	Psap	prosaposin	2					1.55	1.30			
66988	Lap3	leucine aminopeptidase 3	2					1.47	1.30	2	5.7	0.11
270685	Mthfd1l	methylenetetrahydrofolate dehydrogenase (NADP+ dependent) 1-like	2					1.47	1.30			
52530	Nhp2	NHP2 ribonucleoprotein homolog (yeast)	2					1.42	1.30			
54725	Cadm1	cell adhesion molecule 1	2					1.48	1.29	1	2.7	0.38
71839	Osgin1	oxidative stress induced growth inhibitor 1	2					1.40	1.29			
226351	Tmem185b	transmembrane protein 185B	2					1.35	1.29			

Gene ID	Symbol	Description	DEG/Set count	2mWT_2mKO	20mWT_20mKO	2mKO_20mKO	2mWT_20mWT	2mWT_30mWT	20mWT_30mWT	Literature-Mining (Aging-Cholesterol literature)		
				9	230	406	48	1904	1157	#Paper	Enrichment	BH P-value
67239	Rpf2	ribosome production factor 2 homolog (S. cerevisiae)	2					1.37	1.29			
331623	Bend3	BEN domain containing 3	2					1.34	1.28			
70396	Asnsd1	asparagine synthetase domain containing 1	2					1.31	1.28			
15369	Hmox2	heme oxygenase (decycling) 2	2					1.35	1.27	1	4	0.29
11306	Abcb7	ATP-binding cassette, sub-family B (MDR/TAP), member 7	2					1.38	1.27			
66440	Cdc26	cell division cycle 26	2					1.24	1.26			
13014	Cstb	cystatin B	2					1.26	1.26			
16476	Jun	Jun oncogene	2					1.66	1.26	11	2.2	0.06
20720	Serpine2	serine (or cysteine) peptidase inhibitor, clade E, member 2	2					1.35	1.26	2	4.6	0.14
13653	Egr1	early growth response 1	2					1.33	1.26	1	1.2	0.61
102162	Taf5l	TAF5-like RNA polymerase II, p300/CBP-associated factor (PCAF)-associated factor	2					1.45	1.25			
52705	Krr1	KRR1, small subunit (SSU) processome component, homolog (yeast)	2					1.28	1.25			
243914	Lgi4	leucine-rich repeat LGI family, member 4	2					1.39	1.25			
11977	Atp7a	ATPase, Cu++ transporting, alpha polypeptide	2					1.38	1.24			
27361	Sepx1	selenoprotein X 1	2					1.34	1.24	1	17.6	0.12
74315	Rnf145	ring finger protein 145	2					1.33	1.24			
13200	Ddost	dolichyl-di-phosphooligosaccharide-protein glycotransferase	2					1.28	1.23			
77889	Lbh	limb-bud and heart	2					1.30	1.23			
12751	Tpp1	tripeptidyl peptidase I	2					1.29	1.22	1	2.5	0.40
108682	Gpt2	glutamic pyruvate transaminase (alanine aminotransferase) 2	2					1.37	1.22			
211914	Asap2	ArfGAP with SH3 domain, ankyrin repeat and PH domain 2	2					1.32	1.22			
74919	4930471M23Rik	RIKEN cDNA 4930471M23 gene	2					1.21	1.22			
75612	Gns	glucosamine (N-acetyl)-6-sulfatase	2					1.24	1.22			
20971	Sdc4	syndecan 4	2					1.44	1.21			
12345	Capzb	capping protein (actin filament) muscle Z-line, beta	2					1.34	1.21			
66390	Slmo2	slowmo homolog 2 (Drosophila)	2					1.33	1.20			
27984	Efhd2	EF hand domain containing 2	2					1.38	1.20			
19352	Rabggtb	RAB geranylgeranyl transferase, b subunit	2					1.25	1.19			
66125	Sf3b5	splicing factor 3b, subunit 5	2					1.27	1.16			
102462	Imp3	IMP3, U3 small nucleolar ribonucleoprotein, homolog (yeast)	2					1.16	1.16			
68479	Phf5a	PHD finger protein 5A	2					1.15	1.15			
67808	Tprgl	transformation related protein 63 regulated like	2					-1.16	-1.09			
22630	Ywhaq	tyrosine 3-monooxygenase/tryptophan 5-monooxygenase activation protein, theta polypeptide	2					-1.12	-1.10	3	9.7	0.02
20692	Sparc	secreted acidic cysteine rich glycoprotein	2					-1.12	-1.11			
18858	Pmp22	peripheral myelin protein 22	2					-1.22	-1.13	2	8.9	0.06
13179	Dcn	decorin	2					-1.14	-1.14	1	2	0.46
17196	Mbp	myelin basic protein	2					-1.17	-1.14	6	3.4	0.03
66915	Myeov2	myeloma overexpressed 2	2					-1.12	-1.14			
20655	Sod1	superoxide dismutase 1, soluble	2					-1.17	-1.14	46	5.2	2.24E-17
83493	Sacm1l	SAC1 (suppressor of actin mutations 1, homolog)-like (S. cerevisiae)	2					-1.16	-1.14			
56350	Arl3	ADP-ribosylation factor-like 3	2					-1.20	-1.15			
21807	Tsc22d1	TSC22 domain family, member 1	2					-1.15	-1.15			
11737	Anp32a	acidic (leucine-rich) nuclear phosphoprotein 32 family, member A	2					-1.18	-1.16			
69642	2310046A06Rik	RIKEN cDNA 2310046A06 gene	2					-1.24	-1.18			
12575	Cdkn1a	cyclin-dependent kinase inhibitor 1A (P21)	2					-1.23	-1.18	6	1.7	0.25
14677	Gnai1	guanine nucleotide binding protein (G protein), alpha inhibiting 1	2					-1.15	-1.18			
68721	1110032A03Rik	RIKEN cDNA 1110032A03 gene	2					-1.18	-1.18			
100532	Rell1	RELT-like 1	2					-1.25	-1.18			
106581	Itfg3	integrin alpha FG-GAP repeat containing 3	2					-1.45	-1.19			
229877	Rap1gds1	RAP1, GTP-GDP dissociation stimulator 1	2					-1.23	-1.19			
24100	Tpra1	transmembrane protein, adipocyte asscociated 1	2					-1.24	-1.19			
76267	Fads1	fatty acid desaturase 1	2					-1.37	-1.19	3	42.9	4.13E-04
109232	Sccpdh	saccharopine dehydrogenase (putative)	2					-1.17	-1.19			
57342	Parva	parvin, alpha	2					-1.25	-1.19			
18187	Nrp2	neuropilin 2	2					-1.21	-1.20			
68070	Pdzd2	PDZ domain containing 2	2					-1.22	-1.20			
319939	Tns3	tensin 3	2					-1.20	-1.20			
18611	Pea15a	phosphoprotein enriched in astrocytes 15A	2					-1.27	-1.20			
75580	Zbtb4	zinc finger and BTB domain containing 4	2					-1.15	-1.20			
68972	Tatdn3	TatD DNase domain containing 3	2					-1.48	-1.21			
58521	Eid1	EP300 interacting inhibitor of differentiation 1	2					-1.26	-1.21			
246049	Slc36a2	solute carrier family 36 (proton/amino acid symporter), member 2	2					-1.28	-1.21			
224273	Crybg3	beta-gamma crystallin domain containing 3	2					-1.25	-1.21			
252864	Dusp15	dual specificity phosphatase-like 15	2					-1.31	-1.22			
54366	Ctnnal1	catenin (cadherin associated protein), alpha-like 1	2					-1.40	-1.22			

Gene ID	Symbol	Description	DEG/Set count	2mWT_2mKO	20mWT_20mKO	2mKO_20mKO	2mWT_20mWT	2mWT_30mWT	20mWT_30mWT	Literature-Mining (Aging-Cholesterol literature)		
				9	230	406	48	1904	1157	#Paper	Enrichment	BH P-value
23794	Adamts5	a disintegrin-like and metallopeptidase (reprolysin type) with thrombospondin type 1 motif, 5 (aggrecanase-2)	2					-1.31	-1.22			
20525	Slc2a1	solute carrier family 2 (facilitated glucose transporter), member 1	2					-1.23	-1.22	5	5.5	0.01
74392	Specc1l	sperm antigen with calponin homology and coiled-coil domains 1-like	2					-1.25	-1.24			
20203	S100b	S100 protein, beta polypeptide, neural	2					-1.19	-1.24	1	1.3	0.59
68545	Ecscr	endothelial cell-specific chemotaxis regulator	2					-1.41	-1.24			
68652	Tab2	TGF-beta activated kinase 1/MAP3K7 binding protein 2	2					-1.20	-1.24			
245038	Dclk3	doublecortin-like kinase 3	2					-1.27	-1.25			
100503627	Gm17238	predicted gene, 17238	2					-1.56	-1.25			
78287	Zfyve20	zinc finger, FYVE domain containing 20	2					-1.18	-1.25			
237339	L3mbtl3	l(3)mbt-like 3 (Drosophila)	2					-1.51	-1.25			
67812	Ubxn4	UBX domain protein 4	2					-1.28	-1.25			
226525	Rasal2	RAS protein activator like 2	2					-1.28	-1.26			
217692	Sipa1l1	signal-induced proliferation-associated 1 like 1	2					-1.35	-1.26			
21873	Tjp2	tight junction protein 2	2					-1.33	-1.26	1	16.8	0.12
230587	Glis1	GLIS family zinc finger 1	2					-1.34	-1.26			
76960	Bcas1	breast carcinoma amplified sequence 1	2					-1.31	-1.26			
65962	Slc9a3r2	solute carrier family 9 (sodium/hydrogen exchanger), member 3 regulator 2	2					-1.41	-1.26			
78689	Naa35	N(alpha)-acetyltransferase 35, NatC auxiliary subunit	2					-1.29	-1.27			
77569	Limch1	LIM and calponin homology domains 1	2					-1.25	-1.27			
103511	Fam26e	family with sequence similarity 26, member E	2					-1.52	-1.27			
140721	Caskin2	CASK-interacting protein 2	2					-1.52	-1.27			
24066	Spry4	sprouty homolog 4 (Drosophila)	2					-1.35	-1.27			
22288	Utrn	utrophin	2					-1.17	-1.27			
17828	Muted	muted	2					-1.41	-1.27			
72834	2810468N07Rik	RIKEN cDNA 2810468N07 gene	2					-1.37	-1.28			
72634	Tdrkh	tudor and KH domain containing protein	2					-1.26	-1.28			
260305	Nphp4	nephronophthisis 4 (juvenile) homolog (human)	2					-1.38	-1.28			
17475	Mpdz	multiple PDZ domain protein	2					-1.30	-1.28			
83453	Chrdl1	chordin-like 1	2					-1.39	-1.28			
23879	Fxr2	fragile X mental retardation, autosomal homolog 2	2					-1.26	-1.28			
72287	Plekhf1	pleckstrin homology domain containing, family F (with FYVE domain) member 1	2					-1.56	-1.29			
23876	Fbln5	fibulin 5	2					-1.33	-1.29			
71529	Kazn	kazrin, periplakin interacting protein	2					-1.43	-1.29			
50915	Grb14	growth factor receptor bound protein 14	2					-1.39	-1.29			
106369	Ypel1	yippee-like 1 (Drosophila)	2					-1.46	-1.29			
14164	Fgf1	fibroblast growth factor 1	2					-1.36	-1.29			
75744	Svip	small VCP/p97-interacting protein	2					-1.25	-1.29			
17532	Mras	muscle and microspikes RAS	2					-1.47	-1.30			
223649	Nrbp2	nuclear receptor binding protein 2	2					-1.34	-1.30			
13641	Efnb1	ephrin B1	2					-1.26	-1.30			
15468	Prmt2	protein arginine N-methyltransferase 2	2					-1.38	-1.30			
59049	Slc22a17	solute carrier family 22 (organic cation transporter), member 17	2					-1.48	-1.31			
69993	Chn2	chimerin (chimaerin) 2	2					-1.55	-1.31			
26936	Mprip	myosin phosphatase Rho interacting protein	2					-1.48	-1.31			
73182	Pear1	platelet endothelial aggregation receptor 1	2					-1.38	-1.31			
58178	Sorcs1	VPS10 domain receptor protein SORCS 1	2					-1.38	-1.32			
18797	Plcb3	phospholipase C, beta 3	2					-1.25	-1.32			
229658	Vangl1	vang-like 1 (van gogh, Drosophila)	2					-1.52	-1.32			
50530	Mfap5	microfibrillar associated protein 5	2					-1.52	-1.32			
22658	Pcgf2	polycomb group ring finger 2	2					-1.42	-1.33			
64291	Osbpl1a	oxysterol binding protein-like 1A	2					-1.33	-1.33			
94346	Tmem40	transmembrane protein 40	2					-1.62	-1.33			
70354	Secisbp2l	SECIS binding protein 2-like	2					-1.39	-1.33			
70461	Crtc3	CREB regulated transcription coactivator 3	2					-1.87	-1.33			
12389	Cav1	caveolin 1, caveolae protein	2					-1.34	-1.33	14	20.6	5.14E-13
13497	Drp2	dystrophin related protein 2	2					-1.37	-1.33			
224090	Tmem44	transmembrane protein 44	2					-1.40	-1.34			
55992	Trim3	tripartite motif-containing 3	2					-1.52	-1.34			
21802	Tgfa	transforming growth factor alpha	2					-1.51	-1.34	11	2.6	0.02
22051	Trip6	thyroid hormone receptor interactor 6	2					-1.56	-1.34			
79196	Osbpl5	oxysterol binding protein-like 5	2					-1.49	-1.34			
260409	Cdc42ep3	CDC42 effector protein (Rho GTPase binding) 3	2					-1.37	-1.35			
56175	Bace2	beta-site APP-cleaving enzyme 2	2					-1.64	-1.35			
77940	A930004D18Rik	RIKEN cDNA A930004D18 gene	2					-1.48	-1.35			
100380944	Gm11602	predicted gene 11602	2					-1.81	-1.35			
71184	1700112M01Rik	RIKEN cDNA 1700112M01 gene	2					-1.45	-1.35			
110596	Rgnef	Rho-guanine nucleotide exchange factor	2					-1.51	-1.35			
15248	Hic1	hypermethylated in cancer 1	2					-1.70	-1.35			
76898	B3gat1	beta-1,3-glucuronyltransferase 1 (glucuronosyltransferase P)	2					-1.52	-1.35			
50791	Magi2	membrane associated guanylate kinase, WW and PDZ domain containing 2	2					-1.40	-1.36			
226751	Cdc42bpa	CDC42 binding protein kinase alpha	2					-1.37	-1.36			
338367	Myo1d	myosin ID	2					-1.58	-1.36			

Gene ID	Symbol	Description	DEG/Set count	2mWT_2mKO	20mWT_20mKO	2mKO_20mKO	2mWT_20mWT	2mWT_30mWT	20mWT_30mWT	Literature-Mining (Aging-Cholesterol literature)		
				9	230	406	48	1904	1157	#Paper	Enrichment	BH P-value
21981	Ppp1r13b	protein phosphatase 1, regulatory (inhibitor) subunit 13B	2					-1.42	-1.36			
73852	D3Ertd751e	DNA segment, Chr 3, ERATO Doi 751, expressed	2					-1.37	-1.36			
668253	Dleu2	deleted in lymphocytic leukemia, 2	2					-1.43	-1.36			
242653	Cldn19	claudin 19	2					-1.57	-1.37			
24050	Sept3	septin 3	2					-1.42	-1.37			
18821	Pln	phospholamban	2					-1.64	-1.37			
16529	Kcnk5	potassium channel, subfamily K, member 5	2					-1.66	-1.37			
233040	Fbxo27	F-box protein 27	2					-1.67	-1.37			
97761	Sgsm2	small G protein signaling modulator 2	2					-1.37	-1.37			
71213	Cage1	cancer antigen 1	2					-1.53	-1.38			
26427	Creb3l1	cAMP responsive element binding protein 3-like 1	2					-1.49	-1.38			
271849	Shc4	SHC (Src homology 2 domain containing) family, member 4	2					-1.44	-1.38			
21833	Thra	thyroid hormone receptor alpha	2					-1.65	-1.38			
217310	C630004H02Rik	RIKEN cDNA C630004H02 gene	2					-1.49	-1.38			
110606	Fntb	farnesyltransferase, CAAX box, beta	2					-1.55	-1.38			
108800	Ston2	stonin 2	2					-1.60	-1.38			
26968	Islr	immunoglobulin superfamily containing leucine-rich repeat	2					-1.38	-1.39			
18795	Plcb1	phospholipase C, beta 1	2					-1.35	-1.39	5	1.3	0.65
70827	Trak2	trafficking protein, kinesin binding 2	2					-1.30	-1.39			
19272	Ptprk	protein tyrosine phosphatase, receptor type, K	2					-1.43	-1.39			
64297	Gprc5b	G protein-coupled receptor, family C, group 5, member B	2					-1.46	-1.39			
19088	Prkar2b	protein kinase, cAMP dependent regulatory, type II beta	2					-1.36	-1.40			
94332	Cadm3	cell adhesion molecule 3	2					-1.57	-1.40			
241589	D430041D05Rik	RIKEN cDNA D430041D05 gene	2					-1.46	-1.40			
319899	Dock6	dedicator of cytokinesis 6	2					-1.44	-1.40			
11487	Adam10	a disintegrin and metallopeptidase domain 10	2					-1.39	-1.40	5	44.8	1.51E-06
19126	Prom1	prominin 1	2					-1.39	-1.40	1	3.1	0.35
237759	Col23a1	collagen, type XXIII, alpha 1	2					-1.68	-1.41			
234582	Ccdc102a	coiled-coil domain containing 102A	2					-1.78	-1.41			
58802	Kcnmb4	potassium large conductance calcium-activated channel, subfamily M, beta member 4	2					-1.99	-1.41			
329628	Fat4	FAT tumor suppressor homolog 4 (Drosophila)	2					-1.39	-1.41			
23873	Faim	Fas apoptotic inhibitory molecule	2					-1.70	-1.41			
57810	Cdon	cell adhesion molecule-related/down-regulated by oncogenes	2					-1.60	-1.41			
75116	4930520O04Rik	RIKEN cDNA 4930520O04 gene	2					-1.66	-1.41			
18605	Enpp1	ectonucleotide pyrophosphatase/phosphodiesterase 1	2					-1.63	-1.42			
269701	Wdr66	WD repeat domain 66	2					-1.53	-1.42			
14365	Fzd3	frizzled homolog 3 (Drosophila)	2					-1.50	-1.42			
12549	Arhgap31	Rho GTPase activating protein 31	2					-1.36	-1.42			
242466	Zfp462	zinc finger protein 462	2					-1.26	-1.42			
211429	Pla2g4b	phospholipase A2, group IVB (cytosolic)	2					-1.51	-1.42			
13665	Eif2s1	eukaryotic translation initiation factor 2, subunit 1 alpha	2					-1.64	-1.42			
436230	BC065397	cDNA sequence BC065397	2					-1.62	-1.43			
72469	Plcd3	phospholipase C, delta 3	2					-1.69	-1.43			
56788	Scube2	signal peptide, CUB domain, EGF-like 2	2					-2.07	-1.43			
13824	Epb4.1l4a	erythrocyte protein band 4.1-like 4a	2					-1.57	-1.43			
57277	Slurp1	secreted Ly6/Plaur domain containing 1	2					-1.64	-1.43	1	11.5	0.15
381404	Pabpc1l	poly(A) binding protein, cytoplasmic 1-like	2					-2.02	-1.43			
76455	2310067E19Rik	RIKEN cDNA 2310067E19 gene	2					-1.71	-1.43			
17309	Mgat3	mannoside acetylglucosaminyltransferase 3	2					-1.33	-1.43			
194309	Vps37d	vacuolar protein sorting 37D (yeast)	2					-1.56	-1.43			
14199	Fhl1	four and a half LIM domains 1	2					-1.62	-1.43			
105445	Dock9	dedicator of cytokinesis 9	2					-1.43	-1.44			
67374	Jam2	junction adhesion molecule 2	2					-1.48	-1.44			
100503351	Gm19651	predicted gene, 19651	2					-1.69	-1.44			
66338	Cdrt4	CMT1A duplicated region transcript 4	2					-1.64	-1.44			
19273	Ptpru	protein tyrosine phosphatase, receptor type, U	2					-1.61	-1.45	1	2.1	0.44
22420	Wnt6	wingless-related MMTV integration site 6	2					-1.50	-1.45			
78004	Prr15	proline rich 15	2					-1.86	-1.45			
103012	6720401G13Rik	RIKEN cDNA 6720401G13 gene	2					-1.62	-1.45			
217410	Trib2	tribbles homolog 2 (Drosophila)	2					-1.34	-1.45			
226143	Cyp2c44	cytochrome P450, family 2, subfamily c, polypeptide 44	2				-1.95		-1.45			
20378	Frzb	frizzled-related protein	2					-1.36	-1.45			
109323	C1qtnf7	C1q and tumor necrosis factor related protein 7	2					-1.56	-1.45			
333088	Kcp	kielin/chordin-like protein	2					-2.54	-1.46			
269704	Zfp664	zinc finger protein 664	2					-1.55	-1.46			
15426	Hoxc8	homeobox C8	2					-1.67	-1.46			
68525	Evc2	Ellis van Creveld syndrome 2 homolog (human)	2					-1.34	-1.46			
20377	Sfrp1	secreted frizzled-related protein 1	2					-1.50	-1.46			
20475	Six5	sine oculis-related homeobox 5 homolog (Drosophila)	2					-1.78	-1.46			
83396	Glis2	GLIS family zinc finger 2	2					-1.55	-1.47			
16012	Igfbp6	insulin-like growth factor binding protein 6	2					-1.56	-1.47			
12663	Chml	choroideremia-like	2					-1.51	-1.47			

Gene ID	Symbol	Description	DEG/Set count	2mWT_2mKO	20mWT_20mKO	2mKO_20mKO	2mWT_20mWT	2mWT_30mWT	20mWT_30mWT	Literature-Mining (Aging-Cholesterol literature)		
				9	230	406	48	1904	1157	#Paper	Enrichment	BH P-value
223254	Farp1	FERM, RhoGEF (Arhgef) and pleckstrin domain protein 1 (chondrocyte-derived)	2					-1.49	-1.47			
19249	Ptpn13	protein tyrosine phosphatase, non-receptor type 13	2					-1.29	-1.47			
18671	Abcb1a	ATP-binding cassette, sub-family B (MDR/TAP), member 1A	2					-1.36	-1.48	1	0.5	1.02
18004	Nek1	NIMA (never in mitosis gene a)-related expressed kinase 1	2					-1.52	-1.48			
17936	Nab1	Ngfi-A binding protein 1	2					-1.37	-1.48			
214742	Rcor3	REST corepressor 3	2					-1.52	-1.48			
66259	Camk2n1	calcium/calmodulin-dependent protein kinase II inhibitor 1	2					-1.42	-1.48	1	7.2	0.20
68481	Mpzl1	myelin protein zero-like 1	2					-1.53	-1.48			
320635	Cyb5r2	cytochrome b5 reductase 2	2					-1.61	-1.49			
72289	Malat1	metastasis associated lung adenocarcinoma transcript 1 (non-coding RNA)	2					-1.44	-1.49			
72350	Fam164c	family with sequence similarity 164, member C	2					-1.69	-1.49			
68178	Cgnl1	cingulin-like 1	2					-1.29	-1.49			
320965	4831440E17Rik	RIKEN cDNA 4831440E17 gene	2					-2.06	-1.49			
13838	Epha4	Eph receptor A4	2					-1.94	-1.49			
668303	Kif26a	kinesin family member 26A	2					-1.53	-1.50			
232237	Fgd5	FYVE, RhoGEF and PH domain containing 5	2					-1.68	-1.50			
20501	Slc16a1	solute carrier family 16 (monocarboxylic acid transporters), member 1	2					-1.67	-1.50			
14609	Gja1	gap junction protein, alpha 1	2					-1.46	-1.50	1	1	1.04
192897	Itgb4	integrin beta 4	2					-1.46	-1.51			
16886	Limk2	LIM motif-containing protein kinase 2	2					-1.67	-1.51			
109594	Lmo1	LIM domain only 1	2					-1.51	-1.51			
243382	Ppm1k	protein phosphatase 1K (PP2C domain containing)	2					-1.54	-1.51			
77794	Adamtsl2	ADAMTS-like 2	2					-2.03	-1.51			
654818	C030030A07Rik	RIKEN cDNA C030030A07 gene	2					-1.62	-1.51			
18552	Pcsk5	proprotein convertase subtilisin/kexin type 5	2					-1.91	-1.51			
216795	Wnt9a	wingless-type MMTV integration site 9A	2					-1.73	-1.52			
78892	Crispld2	cysteine-rich secretory protein LCCL domain containing 2	2					-1.95	-1.52			
74011	Slc25a27	solute carrier family 25, member 27	2					-1.46	-1.52			
20732	Spint1	serine protease inhibitor, Kunitz type 1	2					-1.93	-1.52			
239114	Il17d	interleukin 17D	2					-1.96	-1.52			
381110	Fam82a1	family with sequence similarity 82, member A1	2					-1.31	-1.53			
192167	Nlgn1	neuroligin 1	2					-1.97	-1.54			
70737	Cgn	cingulin	2					-1.84	-1.54			
230316	Megf9	multiple EGF-like-domains 9	2					-1.41	-1.54			
12724	Clcn2	chloride channel 2	2					-1.83	-1.54			
68939	Rasl11b	RAS-like, family 11, member B	2					-1.82	-1.54			
50905	Il17rb	interleukin 17 receptor B	2					-1.81	-1.54			
214137	Arhgap29	Rho GTPase activating protein 29	2					-1.41	-1.54			
280668	Adam1a	a disintegrin and metallopeptidase domain 1a	2					-1.45	-1.55			
21809	Tgfb3	transforming growth factor, beta 3	2					-1.46	-1.55			
12395	Runx1t1	runt-related transcription factor 1; translocated to, 1 (cyclin D-related)	2					-1.46	-1.55			
327958	Pitpnm3	PITPNM family member 3	2					-1.44	-1.55			
73490	Mipol1	mirror-image polydactyly gene 1 homolog (human)	2					-1.39	-1.55			
207259	Zbtb7c	zinc finger and BTB domain containing 7C	2					-1.59	-1.56			
56089	Ramp3	receptor (calcitonin) activity modifying protein 3	2					-2.09	-1.56			
15465	Hrh1	histamine receptor H1	2					-1.53	-1.56			
69219	Ddah1	dimethylarginine dimethylaminohydrolase 1	2					-1.84	-1.56	1	15.4	0.13
23955	Nek4	NIMA (never in mitosis gene a)-related expressed kinase 4	2					-1.37	-1.57			
268759	9930012K11Rik	RIKEN cDNA 9930012K11 gene	2					-1.87	-1.57			
54612	Sfrp5	secreted frizzled-related sequence protein 5	2					-1.44	-1.57			
319832	Tmem229a	transmembrane protein 229A	2					-1.60	-1.57			
97114	Hist2h3c2-ps	histone cluster 2, H3c2, pseudogene	2					-1.77	-1.57			
67434	Ankrd33b	ankyrin repeat domain 33B	2					-1.59	-1.57			
54446	Nfat5	nuclear factor of activated T-cells 5	2					-1.49	-1.58			
11551	Adra2a	adrenergic receptor, alpha 2a	2					-2.08	-1.58			
53881	Slc5a3	solute carrier family 5 (inositol transporters), member 3	2					-1.37	-1.59			
117606	Boc	biregional cell adhesion molecule-related/down-regulated by oncogenes (Cdon) binding proteir	2					-1.57	-1.60			
76640	1700113H08Rik	RIKEN cDNA 1700113H08 gene	2					-1.97	-1.60			
18511	Pax9	paired box gene 9	2					-1.82	-1.60			
70784	Rasl12	RAS-like, family 12	2					-1.85	-1.60			
100042480	Nhsl2	NHS-like 2	2					-1.34	-1.60			
14178	Fgf7	fibroblast growth factor 7	2					-1.34	-1.60			
17301	Foxd2	forkhead box D2	2					-1.54	-1.61			
100504377	Gm20199	predicted gene, 20199	2					-1.59	-1.61			
75906	Fam184a	family with sequence similarity 184, member A	2					-1.46	-1.61			
16475	Jub	ajuba	2					-1.95	-1.61			
12654	Chi3l1	chitinase 3-like 1	2					-1.62	-1.61			
58237	Nkain4	Na+/K+ transporting ATPase interacting 4	2					-1.54	-1.62			
16764	Aff3	AF4/FMR2 family, member 3	2					-1.37	-1.62			
223864	Rapgef3	Rap guanine nucleotide exchange factor (GEF) 3	2					-1.51	-1.62			

Gene ID	Symbol	Description	DEG/Set count	2mWT_2mKO	20mWT_20mKO	2mKO_20mKO	2mWT_20mWT	2mWT_30mWT	20mWT_30mWT	Literature-Mining (Aging-Cholesterol literature)		
				9	230	406	48	1904	1157	#Paper	Enrichment	BH P-value
100039684	5031434O11Rik	RIKEN cDNA 5031434O11 gene	2					-1.87	-1.62			
108760	Galntl1	UDP-N-acetyl-alpha-D-galactosamine:polypeptide N-acetylgalactosaminyltransferase-like 1	2					-1.81	-1.63			
212391	Lcor	ligand dependent nuclear receptor corepressor	2					-1.63	-1.63			
78102	8430426J06Rik	RIKEN cDNA 8430426J06 gene	2					-1.95	-1.63			
74782	Glt8d2	glycosyltransferase 8 domain containing 2	2					-1.94	-1.63			
100039795	Ildr2	immunoglobulin-like domain containing receptor 2	2					-1.48	-1.63			
209645	Bend7	BEN domain containing 7	2					-2.19	-1.64			
233274	Siglech	sialic acid binding Ig-like lectin H	2					-1.93	-1.64			
12824	Col2a1	collagen, type II, alpha 1	2					-1.79	-1.64			
114142	Foxp2	forkhead box P2	2					-1.55	-1.64			
68235	2410066E13Rik	RIKEN cDNA 2410066E13 gene	2					-1.46	-1.64			
114249	Npnt	nephronectin	2					-1.82	-1.65			
12160	Bmp5	bone morphogenetic protein 5	2					-1.74	-1.66			
17965	Nbl1	neuroblastoma, suppression of tumorigenicity 1	2					-1.56	-1.66			
22351	Vill	villin-like	2					-1.71	-1.66			
63830	Kcnq1ot1	KCNQ1 overlapping transcript 1	2					-1.87	-1.67			
16456	F11r	F11 receptor	2					-1.59	-1.67	3	26.9	1.43E-03
106522	Pkdcc	protein kinase domain containing, cytoplasmic	2					-1.77	-1.67			
100504686	Sfpq	NA	2					-1.62	-1.67			
16526	Kcnk2	potassium channel, subfamily K, member 2	2					-1.58	-1.67			
241638	Prosapip1	ProSAPiP1 protein	2					-1.85	-1.68			
17057	Klrb1a	killer cell lectin-like receptor subfamily B member 1A	2					-1.91	-1.68			
268860	Abat	4-aminobutyrate aminotransferase	2					-1.53	-1.68			
237761	Ankrd43	ankyrin repeat domain 43	2					-1.63	-1.69			
68144	5031426D15Rik	RIKEN cDNA 5031426D15 gene	2					-1.66	-1.69			
320311	Rnf152	ring finger protein 152	2					-1.74	-1.70			
14200	Fhl2	four and a half LIM domains 2	2					-1.64	-1.71			
14632	Gli1	GLI-Kruppel family member GLI1	2					-1.68	-1.71			
12829	Col4a4	collagen, type IV, alpha 4	2					-2.01	-1.71			
66790	Grtp1	GH regulated TBC protein 1	2					-1.97	-1.72			
107587	Osr2	odd-skipped related 2 (Drosophila)	2					-1.84	-1.73			
93842	Igsf9	immunoglobulin superfamily, member 9	2					-2.35	-1.73			
242509	Bnc2	basonuclin 2	2					-1.65	-1.73			
60596	Gucy1a3	guanylate cyclase 1, soluble, alpha 3	2					-1.90	-1.73			
109978	Art4	ADP-ribosyltransferase 4	2					-2.28	-1.73			
240332	Slc6a7	solute carrier family 6 (neurotransmitter transporter, L-proline), member 7	2					-1.94	-1.73			
73296	Rhobtb3	Rho-related BTB domain containing 3	2					-1.78	-1.74			
269959	Adamtsl3	ADAMTS-like 3	2					-1.65	-1.74			
74134	Cyp2s1	cytochrome P450, family 2, subfamily s, polypeptide 1	2					-2.18	-1.74			
13052	Cxadr	coxsackie virus and adenovirus receptor	2					-1.48	-1.74	1	5.1	0.25
100502711		NA	2					-1.59	-1.74			
13386	Dlk1	delta-like 1 homolog (Drosophila)	2					-2.77	-1.74			
12737	Cldn1	claudin 1	2					-1.65	-1.75			
106042	Prickle1	prickle homolog 1 (Drosophila)	2					-1.70	-1.75			
70598	Filip1	filamin A interacting protein 1	2					-1.70	-1.75			
20518	Slc22a2	solute carrier family 22 (organic cation transporter), member 2	2					-2.23	-1.75			
333182	Cox6b2	cytochrome c oxidase subunit VIb polypeptide 2	2					-2.18	-1.76			
320916	Wscd2	WSC domain containing 2	2					-1.84	-1.77			
17286	Meox2	mesenchyme homeobox 2	2					-1.91	-1.78			
21828	Thbs4	thrombospondin 4	2					-1.72	-1.78			
76365	Tbx18	T-box18	2					-1.81	-1.78			
14412	Slc6a13	solute carrier family 6 (neurotransmitter transporter, GABA), member 13	2					-1.57	-1.79			
320415	Gchfr	GTP cyclohydrolase I feedback regulator	2					-2.83	-1.79			
23964	Odz2	odd Oz/ten-m homolog 2 (Drosophila)	2					-1.92	-1.80			
60345	Nrip2	nuclear receptor interacting protein 2	2					-1.85	-1.80			
77132	2810433D01Rik	RIKEN cDNA 2810433D01 gene	2					-2.03	-1.81			
108096	Slco1a5	solute carrier organic anion transporter family, member 1a5	2					-2.53	-1.81			
12224	Klf5	Kruppel-like factor 5	2					-1.72	-1.83			
57265	Fzd2	frizzled homolog 2 (Drosophila)	2					-1.71	-1.83			
69239	2610034M16Rik	RIKEN cDNA 2610034M16 gene	2					-1.67	-1.84			
100043364	Gm4392	predicted gene 4392	2					-1.62	-1.84			
78303	Hist3h2ba	histone cluster 3, H2ba	2					-2.05	-1.84			
67198	Spats2l	spermatogenesis associated, serine-rich 2-like	2					-2.01	-1.85			
71951	Gpc2	glypican 2 (cerebroglycan)	2					-1.57	-1.86			
108105	B3gnt5	UDP-GlcNAc:betaGal beta-1,3-N-acetylglucosaminyltransferase 5	2				1.65		-1.86			
319819	4932435O22Rik	RIKEN cDNA 4932435O22 gene	2					-1.51	-1.87			
228413	Prrg4	proline rich Gla (G-carboxyglutamic acid) 4 (transmembrane)	2					-1.86	-1.87			
100503921	Gm19966	predicted gene, 19966	2					-2.36	-1.87			
23888	Gpc6	glypican 6	2					-1.97	-1.88			
14012	Mpzl2	myelin protein zero-like 2	2					-1.79	-1.89			
330096	Shisa3	shisa homolog 3 (Xenopus laevis)	2					-1.65	-1.90			
216033	Ctnna3	catenin (cadherin associated protein), alpha 3	2					-1.76	-1.90			
22284	Usp9x	ubiquitin specific peptidase 9, X chromosome	2				1.39		-1.90			

Gene ID	Symbol	Description	DEG/Set count	2mWT_2mKO	20mWT_20mKO	2mKO_20mKO	2mWT_20mWT	2mWT_30mWT	20mWT_30mWT	Literature-Mining (Aging-Cholesterol literature)		
				9	230	406	48	1904	1157	#Paper	Enrichment	BH P-value
64058	Perp	PERP, TP53 apoptosis effector	2					-1.95	-1.91			
57738	Slc15a2	solute carrier family 15 (H+/peptide transporter), member 2	2					-2.37	-1.92			
330166	Miat	myocardial infarction associated transcript (non-protein coding)	2					-2.00	-1.93			
24059	Slco2a1	solute carrier organic anion transporter family, member 2a1	2					-1.81	-1.95			
78267	Klhdc8b	kelch domain containing 8B	2					-2.26	-1.95			
68625	Wdr65	WD repeat domain 65	2					-1.83	-1.96			
209195	Clic6	chloride intracellular channel 6	2					-1.69	-1.96			
15229	Foxd1	forkhead box D1	2					-1.73	-1.96			
13884	Ces1c	carboxylesterase 1C	2					-2.33	-1.97			
69772	Bdh2	3-hydroxybutyrate dehydrogenase, type 2	2					-2.40	-1.98			
64074	Smoc2	SPARC related modular calcium binding 2	2					-2.06	-1.98			
74186	Ccdc3	coiled-coil domain containing 3	2					-1.95	-1.99			
16612	Klk1	kallikrein 1	2					-2.02	-2.00			
223272	Itgbl1	integrin, beta-like 1	2					-2.18	-2.00			
55987	Cpxm2	carboxypeptidase X 2 (M14 family)	2					-1.81	-2.00			
66859	Slc16a9	solute carrier family 16 (monocarboxylic acid transporters), member 9	2					-1.86	-2.03			
320856	Ppp1r12b	NA	2					-2.52	-2.05			
16763	Lad1	ladinin	2					-2.52	-2.07			
224833	AI661453	expressed sequence AI661453	2					-2.10	-2.09			
100040792	Gm11627	predicted gene 11627	2					-2.06	-2.09			
654812	Angptl7	angiopoietin-like 7	2					-2.24	-2.09			
20519	Slc22a3	solute carrier family 22 (organic cation transporter), member 3	2					-2.22	-2.09			
56047	Msln	mesothelin	2					-1.95	-2.10			
14275	Folr1	folate receptor 1 (adult)	2					-1.64	-2.11			
74103	Nebl	nebulette	2				1.88		-2.17			
100504147	LOC100504147	NA	2					-2.10	-2.17			
16782	Lamc2	laminin, gamma 2	2					-2.40	-2.18	2	3	0.22
12373	Casq2	calsequestrin 2	2					-2.29	-2.21			
14264	Fmod	fibromodulin	2					-2.82	-2.29			
319626	9530059O14Rik	RIKEN cDNA 9530059O14 gene	2					-1.82	-2.31			
192199	Rspo1	R-spondin homolog (Xenopus laevis)	2					-2.57	-2.35			
108151	Sema3d	sema domain, immunoglobulin domain (Ig), short basic domain, secreted, (semaphorin) 3D	2					-2.22	-2.36			
16780	Lamb3	laminin, beta 3	2					-2.38	-2.36			
18784	Pla2g5	phospholipase A2, group V	2					-2.16	-2.36			
14411	Slc6a12	solute carrier family 6 (neurotransmitter transporter, betaine/GABA), member 12	2					-2.52	-2.37			
399558	Flrt2	fibronectin leucine rich transmembrane protein 2	2					-2.02	-2.38			
75677	Cldn22	claudin 22	2					-2.91	-2.40			
338403	Cndp1	carnosine dipeptidase 1 (metallopeptidase M20 family)	2					-2.89	-2.41			
70008	Ace2	angiotensin I converting enzyme (peptidyl-dipeptidase A) 2	2					-3.80	-2.49			
16425	Itih2	inter-alpha trypsin inhibitor, heavy chain 2	2					-2.88	-2.52			
99681	Tchh	trichohyalin	2					-2.46	-2.55			
71664	Mettl7b	methyltransferase like 7B	2					-2.85	-2.60			
68311	Lypd2	Ly6/Plaur domain containing 2	2					-3.77	-2.91			
16669	Krt19	keratin 19	2					-3.20	-3.07			
54156	Egfl6	EGF-like-domain, multiple 6	2					-4.95	-3.22			
74071	Ifltd1	intermediate filament tail domain containing 1	2					-3.38	-3.24			
13643	Efnb3	ephrin B3	2					-4.71	-3.69			
665033	Col6a5	collagen, type VI, alpha 5	2					-5.42	-3.79			
20186	Nr1h4	nuclear receptor subfamily 1, group H, member 4	2		1.50			2.16		5	23.3	3.01E-05
58807	Slco1c1	solute carrier organic anion transporter family, member 1c1	2		-1.54			1.73				
56857	Slc37a2	solute carrier family 37 (glycerol-3-phosphate transporter), member 2	2		1.41			2.49				
65221	Slc15a3	solute carrier family 15, member 3	2		1.72			4.82				
93880	Pcdhb9	protocadherin beta 9	2				1.97	2.93				
17916	Myo1f	myosin IF	2		1.94			3.35				
214944	Mob3b	MOB kinase activator 3B	2		1.35			-1.56				
319387	Lphn3	latrophilin 3	2	1.88				1.73				
16792	Laptm5	lysosomal-associated protein transmembrane 5	2		1.48			3.39				
319765	Igf2bp2	insulin-like growth factor 2 mRNA binding protein 2	2				-1.47	-1.52				
100503148	Gm19575	NA	2				-1.57	-2.03				
338521	Fa2h	fatty acid 2-hydroxylase	2				-1.32	-1.41				
56405	Dusp14	dual specificity phosphatase 14	2		-1.58			-1.52				
12903	Crabp1	cellular retinoic acid binding protein I	2	-2.23	-2.60							
12834	Col6a2	collagen, type VI, alpha 2	2				-1.69	-2.14				
19378	Aldh1a2	aldehyde dehydrogenase family 1, subfamily A2	2		-2.26			2.50				
109934	Abr	active BCR-related gene	2				1.53	1.65				
100503029		NA	2		1.40			-1.77				
20201	S100a8	S100 calcium binding protein A8 (calgranulin A)	1			9.40						
624219	Gm6484	predicted gene 6484	1			8.97						
20202	S100a9	S100 calcium binding protein A9 (calgranulin B)	1			7.38						
83379	Klb	klotho beta	1			6.67						
17200	Mc2r	melanocortin 2 receptor	1			4.38						
18406	Orm2	orosomucoid 2	1			3.90				3	5.1	0.06

Gene ID	Symbol	Description	DEG/Set count	2mWT_2mKO	20mWT_20mKO	2mKO_20mKO	2mWT_20mWT	2mWT_30mWT	20mWT_30mWT	Literature-Mining (Aging-Cholesterol literature)		
				9	230	406	48	1904	1157	#Paper	Enrichment	BH P-value
22095	Tshr	thyroid stimulating hormone receptor	1			3.85						
226049	Dmrt2	doublesex and mab-3 related transcription factor 2	1			3.74						
116939	Pnpla3	patatin-like phospholipase domain containing 3	1			3.66				1	46.4	0.06
21835	Thrsp	thyroid hormone responsive SPOT14 homolog (Rattus)	1			3.49				1	12.8	0.15
69189	1810033B17Rik	RIKEN cDNA 1810033B17 gene	1			3.34						
69861	2010003K11Rik	RIKEN cDNA 2010003K11 gene	1			3.25						
244416	Ppp1r3b	protein phosphatase 1, regulatory (inhibitor) subunit 3B	1			3.04						
15203	Heph	hephaestin	1			2.98				1	25.9	0.09
319743	9630013D21Rik	RIKEN cDNA 9630013D21 gene	1			2.95						
79456	Recql4	RecQ protein-like 4	1			2.87						
56078	Car5b	carbonic anhydrase 5b, mitochondrial	1			2.66						
17001	Ltc4s	leukotriene C4 synthase	1			2.62						
13809	Enpep	glutamyl aminopeptidase	1			2.44						
319942	A530016L24Rik	RIKEN cDNA A530016L24 gene	1			2.44						
56485	Slc2a5	solute carrier family 2 (facilitated glucose transporter), member 5	1			2.41				1	19.5	0.11
14562	Gdf3	growth differentiation factor 3	1			2.40						
18639	Pfkfb1	6-phosphofructo-2-kinase/fructose-2,6-biphosphatase 1	1			2.38						
18407	Orm3	orosomucoid 3	1			2.38						
67800	Dgat2	diacylglycerol O-acyltransferase 2	1			2.37				2	71.1	2.41E-03
18631	Pex11a	peroxisomal biogenesis factor 11 alpha	1			2.31						
18030	Nfil3	nuclear factor, interleukin 3, regulated	1			2.22						
72121	Dennd2d	DENN/MADD domain containing 2D	1			2.21						
67468	Mmd	monocyte to macrophage differentiation-associated	1			2.16						
26358	Aldh1a7	aldehyde dehydrogenase family 1, subfamily A7	1			2.09						
14874	Gstz1	glutathione transferase zeta 1 (maleylacetoacetate isomerase)	1			2.00						
73699	Ppp2r1b	protein phosphatase 2 (formerly 2A), regulatory subunit A (PR 65), beta isoform	1			1.99						
245945	Rbm47	RNA binding motif protein 47	1			1.99						
20733	Spint2	serine protease inhibitor, Kunitz type 2	1			1.94						
330189	Tmem120b	transmembrane protein 120B	1			1.93						
11556	Adrb3	adrenergic receptor, beta 3	1			1.93				3	31.9	9.29E-04
20962	Sycp3	synaptonemal complex protein 3	1			1.90						
112407	Egln3	EGL nine homolog 3 (C. elegans)	1			1.88						
192200	Wfdc12	WAP four-disulfide core domain 12	1			1.85						
21857	Timp1	tissue inhibitor of metalloproteinase 1	1			1.84				2	1.1	0.74
494504	Apcdd1	adenomatosis polyposis coli down-regulated 1	1			1.83						
14257	Flt4	FMS-like tyrosine kinase 4	1			1.81						
118449	Synpo2	synaptopodin 2	1			1.80						
18563	Pcx	pyruvate carboxylase	1			1.80						
67182	Pdzk1ip1	PDZK1 interacting protein 1	1			1.79						
76041	Ccdc125	coiled-coil domain containing 125	1			1.79						
76282	Gpt	glutamic pyruvic transaminase, soluble	1			1.77				32	9.4	3.70E-19
212862	Chpt1	choline phosphotransferase 1	1			1.77				3	19.5	3.22E-03
14104	Fasn	fatty acid synthase	1			1.73				10	15.1	3.36E-08
60525	Acss2	acyl-CoA synthetase short-chain family member 2	1			1.72						
54120	Gipc2	GIPC PDZ domain containing family, member 2	1			1.72						
14555	Gpd1	glycerol-3-phosphate dehydrogenase 1 (soluble)	1			1.71						
241694	Ralgapa2	Ral GTPase activating protein, alpha subunit 2 (catalytic)	1			1.69						
13609	S1pr1	sphingosine-1-phosphate receptor 1	1			1.68						
11637	Ak2	adenylate kinase 2	1			1.67						
277414	Trp53i11	transformation related protein 53 inducible protein 11	1			1.67						
13120	Cyp4b1	cytochrome P450, family 4, subfamily b, polypeptide 1	1			1.66						
72472	Slc16a10	solute carrier family 16 (monocarboxylic acid transporters), member 10	1			1.64						
19885	Rorc	RAR-related orphan receptor gamma	1			1.63						
235135	Tmem45b	transmembrane protein 45b	1			1.63						
14792	Lpcat3	lysophosphatidylcholine acyltransferase 3	1			1.61						
243374	Gimap8	GTPase, IMAP family member 8	1			1.60						
71693	Colec11	collectin sub-family member 11	1			1.59						
11861	Arl4a	ADP-ribosylation factor-like 4A	1			1.56						
240168	Rasgrp3	RAS, guanyl releasing protein 3	1			1.55						
19011	Endou	endonuclease, polyU-specific	1			1.54						
317758	Gimap9	GTPase, IMAP family member 9	1			1.54						
14396	Gabra3	gamma-aminobutyric acid (GABA) A receptor, subunit alpha 3	1			1.53						
22341	Vegfc	vascular endothelial growth factor C	1			1.50						
107476	Acaca	acetyl-Coenzyme A carboxylase alpha	1			1.49				3	9.1	0.02
56738	Mocs1	molybdenum cofactor synthesis 1	1			1.49						
15064	Mr1	major histocompatibility complex, class I-related	1			1.48						
66307	Isoc1	isochorismatase domain containing 1	1			1.48						
110208	Pgd	phosphogluconate dehydrogenase	1			1.46				2	4	0.16
228026	Pdk1	pyruvate dehydrogenase kinase, isoenzyme 1	1			1.46						
12580	Cdkn2c	cyclin-dependent kinase inhibitor 2C (p18, inhibits CDK4)	1			1.45						

Gene ID	Symbol	Description	DEG/Set count	2mWT_2mKO	20mWT_20mKO	2mKO_20mKO	2mWT_20mWT	2mWT_30mWT	20mWT_30mWT	Literature-Mining (Aging-Cholesterol literature)		
				9	230	406	48	1904	1157	#Paper	Enrichment	BH P-value
503610	Zdhhc18	zinc finger, DHHC domain containing 18	1			1.44						
78088	Ankrd56	ankyrin repeat domain 56	1			1.42						
233789	Smg1	SMG1 homolog, phosphatidylinositol 3-kinase-related kinase (C. elegans)	1			1.42						
67384	Bag4	BCL2-associated athanogene 4	1			1.41						
14594	Ggta1	glycoprotein galactosyltransferase alpha 1, 3	1			1.41						
238799	Tnpo1	transportin 1	1			1.40				1	5.5	0.24
100087	Kti12	KTI12 homolog, chromatin associated (S. cerevisiae)	1			1.40						
67824	Nmral1	NmrA-like family domain containing 1	1			1.40						
11540	Adora2a	adenosine A2a receptor	1			1.39						
320302	Glt28d2	glycosyltransferase 28 domain containing 2	1			1.38						
76306	1110021L09Rik	RIKEN cDNA 1110021L09 gene	1			1.38						
66949	Trim59	tripartite motif-containing 59	1			1.38						
317757	Gimap5	GTPase, IMAP family member 5	1			1.38						
240505	Cdc42bpg	CDC42 binding protein kinase gamma (DMPK-like)	1			1.38						
20969	Sdc1	syndecan 1	1			1.37						
193740	Hspa1a	heat shock protein 1A	1			1.37				13	3.2	1.93E-03
19205	Ptbp1	polypyrimidine tract binding protein 1	1			1.36						
15904	Id4	inhibitor of DNA binding 4	1			1.35						
18018	Nfatc1	nuclear factor of activated T-cells, cytoplasmic, calcineurin-dependent 1	1			1.34						
21938	Tnfrsf1b	tumor necrosis factor receptor superfamily, member 1b	1			1.34				2	4.4	0.15
268291	Rnf217	ring finger protein 217	1			1.34						
214987	Chtf8	CTF8, chromosome transmission fidelity factor 8 homolog (S. cerevisiae)	1			1.34						
71683	Gypc	glycophorin C	1			1.33						
100040608	Fancf	Fanconi anemia, complementation group F	1			1.33						
17436	Me1	malic enzyme 1, NADP(+)-dependent, cytosolic	1			1.33				3	2.5	0.19
17064	Cd93	CD93 antigen	1			1.32						
69674	Mif4gd	MIF4G domain containing	1			1.32						
432572	Specc1	sperm antigen with calponin homology and coiled-coil domains 1	1			1.32						
140483	Hnmt	histamine N-methyltransferase	1			1.31						
20723	Serpinb9	serine (or cysteine) peptidase inhibitor, clade B, member 9	1			1.31						
17215	Mcm3	minichromosome maintenance deficient 3 (S. cerevisiae)	1			1.30						
11745	Anxa3	annexin A3	1			1.29				1	47	0.06
228889	Ddx27	DEAD (Asp-Glu-Ala-Asp) box polypeptide 27	1			1.28						
104112	Acly	ATP citrate lyase	1			1.28				1	4.7	0.27
18104	Nqo1	NAD(P)H dehydrogenase, quinone 1	1			1.27				2	2.3	0.30
66841	Etfdh	electron transferring flavoprotein, dehydrogenase	1			1.26						
230484	Usp1	ubiquitin specific peptidase 1	1			1.24						
17250	Abcc1	ATP-binding cassette, sub-family C (CFTR/MRP), member 1	1			1.23				2	2.5	0.27
66230	Mrps28	mitochondrial ribosomal protein S28	1			1.23						
68292	Stt3b	STT3, subunit of the oligosaccharyltransferase complex, homolog B (S. cerevisiae)	1			1.22						
73078	Pmpcb	peptidase (mitochondrial processing) beta	1			1.21						
17975	Ncl	nucleolin	1			1.20						
70508	Bbx	bobby sox homolog (Drosophila)	1			1.20						
13664	Eif1a	eukaryotic translation initiation factor 1A	1			1.19						
18813	Pa2g4	proliferation-associated 2G4	1			1.19						
66375	Dhrs7	dehydrogenase/reductase (SDR family) member 7	1			1.19						
13033	Ctsd	cathepsin D	1			1.17				2	2	0.33
12974	Cs	citrate synthase	1			1.17				1	1.2	0.60
68263	Pdhb	pyruvate dehydrogenase (lipoamide) beta	1			1.16				3	3	0.15
70564	5730469M10Rik	RIKEN cDNA 5730469M10 gene	1			1.16						
22333	Vdac1	voltage-dependent anion channel 1	1			-1.10				2	6.3	0.10
71765	Klhdc3	kelch domain containing 3	1			-1.19						
79566	Sh3bp5l	SH3 binding domain protein 5 like	1			-1.21						
209378	Itih5	inter-alpha (globulin) inhibitor H5	1			-1.24						
215008	Vezt	vezatin, adherens junctions transmembrane protein	1			-1.25						
18822	Plod1	procollagen-lysine, 2-oxoglutarate 5-dioxygenase 1	1			-1.25						
14073	Faah	fatty acid amide hydrolase	1			-1.25						
67046	Tbc1d7	TBC1 domain family, member 7	1			-1.28						
667742	Fam38b	family with sequence similarity 38, member B	1			-1.28						
17207	Mcf2l	mcf.2 transforming sequence-like	1			-1.29						
192734	AI646023	expressed sequence AI646023	1			-1.29						
212919	Kctd7	potassium channel tetramerisation domain containing 7	1			-1.31						
319504	Nrcam	neuron-glia-CAM-related cell adhesion molecule	1			-1.32						
64339	Fndc4	fibronectin type III domain containing 4	1			-1.33						
16773	Lama2	laminin, alpha 2	1			-1.34						
18761	Prkcq	protein kinase C, theta	1			-1.36						
93734	Mpv17l	Mpv17 transgene, kidney disease mutant-like	1			-1.36						
12323	Camk2b	calcium/calmodulin-dependent protein kinase II, beta	1			-1.37						

Gene ID	Symbol	Description	DEG/Set count	2mWT_2mKO	20mWT_20mKO	2mKO_20mKO	2mWT_20mWT	2mWT_30mWT	20mWT_30mWT	Literature-Mining (Aging-Cholesterol literature)		
				9	230	406	48	1904	1157	#Paper	Enrichment	BH P-value
77552	Shisa4	shisa homolog 4 (Xenopus laevis)	1			-1.37						
109264	Me3	malic enzyme 3, NADP(+)-dependent, mitochondrial	1			-1.38						
233724	Tmem41b	transmembrane protein 41B	1			-1.41						
73720	Cst6	cystatin E/M	1			-1.41						
268970	Arhgap28	Rho GTPase activating protein 28	1			-1.43						
215690	Nav1	neuron navigator 1	1			-1.44						
12842	Col1a1	collagen, type I, alpha 1	1			-1.46				47	1.9	6.44E-04
622675	Zfp827	zinc finger protein 827	1			-1.46						
14026	Evl	Ena-vasodilator stimulated phosphoprotein	1			-1.47						
100504154	Gm15050	predicted gene 15050	1			-1.50						
227541	Camk1d	calcium/calmodulin-dependent protein kinase ID	1			-1.50						
140492	Kcnn2	potassium intermediate/small conductance calcium-activated channel, subfamily N, member 2	1			-1.51						
100504135	Gm20081	predicted gene, 20081	1			-1.52						
99031	Osbpl6	oxysterol binding protein-like 6	1			-1.52						
14190	Fgl2	fibrinogen-like protein 2	1			-1.54						
65115	Bean1	brain expressed, associated with Nedd4, 1	1			-1.61						
17968	Ncam2	neural cell adhesion molecule 2	1			-1.61						
74166	Tmem38a	transmembrane protein 38A	1			-1.62						
235281	Scn3b	sodium channel, voltage-gated, type III, beta	1			-1.66						
78925	Srd5a1	steroid 5 alpha-reductase 1	1			-1.66				3	6.3	0.04
16816	Lcat	lecithin cholesterol acyltransferase	1			-1.70				31	84.4	2.21E-46
12830	Col4a5	collagen, type IV, alpha 5	1			-1.71						
381633	Gm1673	predicted gene 1673	1			-1.74						
73287	1700040L02Rik	RIKEN cDNA 1700040L02 gene	1			-1.74						
100504205	Kcnh8	NA	1			-1.74						
16322	Inha	inhibin alpha	1			-1.76						
15413	Hoxb5	homeobox B5	1			-1.79						
23937	Mab21l2	mab-21-like 2 (C. elegans)	1			-1.79						
384061	Fndc5	fibronectin type III domain containing 5	1			-1.82						
320460	Vwc2l	von Willebrand factor C domain-containing protein 2-like	1			-1.90						
17263	Meg3	maternally expressed 3	1			-1.92						
78977	Popdc3	popeye domain containing 3	1			-1.96						
18802	Plcd4	phospholipase C, delta 4	1			-2.03						
27281	Hrasls	HRAS-like suppressor	1			-2.09						
218624	Il31ra	interleukin 31 receptor A	1			-2.11						
330119	Adamts3	a disintegrin-like and metallopeptidase (reprolysin type) with thrombospondin type 1 motif, 3	1			-2.12						
22771	Zic1	zinc finger protein of the cerebellum 1	1			-2.33						
545527	Fam194a	family with sequence similarity 194, member A	1			-2.41						
15357	Hmgcr	3-hydroxy-3-methylglutaryl-Coenzyme A reductase	1			-2.54				48	110.7	2.64E-77
67473	Slc47a1	solute carrier family 47, member 1	1			-2.60						
108907	Nusap1	nucleolar and spindle associated protein 1	1						5.71			
14191	Fgr	Gardner-Rasheed feline sarcoma viral (Fgr) oncogene homolog	1						4.35			
105418	E330034G19Rik	RIKEN cDNA E330034G19 gene	1						3.74			
64011	Nrgn	neurogranin	1						3.61			
12229	Btk	Bruton agammaglobulinemia tyrosine kinase	1						3.55			
19253	Ptpn18	protein tyrosine phosphatase, non-receptor type 18	1						3.23			
22376	Was	Wiskott-Aldrich syndrome homolog (human)	1						3.08			
216984	Evi2b	ecotropic viral integration site 2b	1						2.98			
78317	Ccdc88b	coiled-coil domain containing 88B	1						2.91			
16985	Lsp1	lymphocyte specific 1	1						2.90			
17096	Lyn	Yamaguchi sarcoma viral (v-yes-1) oncogene homolog	1						2.77	1	4.6	0.27
224860	Plcl2	phospholipase C-like 2	1						2.74			
12534	Cdk1	cyclin-dependent kinase 1	1						2.65			
66977	Nuf2	NUF2, NDC80 kinetochore complex component, homolog (S. cerevisiae)	1						2.60			
27405	Abcg3	ATP-binding cassette, sub-family G (WHITE), member 3	1						2.56			
30955	Pik3cg	phosphoinositide-3-kinase, catalytic, gamma polypeptide	1						2.52	1	1.2	0.62
69987	1700026L06Rik	RIKEN cDNA 1700026L06 gene	1						2.52			
67177	Cdt1	chromatin licensing and DNA replication factor 1	1						2.45			
15366	Hmmr	hyaluronan mediated motility receptor (RHAMM)	1						2.42			
106878	2010002N04Rik	RIKEN cDNA 2010002N04 gene	1						2.37			
66329	Susd3	sushi domain containing 3	1						2.32			
50931	Il27ra	interleukin 27 receptor, alpha	1						2.31			
14526	Gcg	glucagon	1						2.28	1	1	0.67
66929	Asf1b	ASF1 anti-silencing function 1 homolog B (S. cerevisiae)	1						2.26			
321019	Gpr183	G protein-coupled receptor 183	1						2.23			
22256	Ung	uracil DNA glycosylase	1						2.21			
217166	Nr1d1	nuclear receptor subfamily 1, group D, member 1	1						2.20			
56489	Ikbke	inhibitor of kappaB kinase epsilon	1						2.20			
74039	Nfam1	Nfat activating molecule with ITAM motif 1	1						2.13			
70719	Hmha1	histocompatibility (minor) HA-1	1						2.11			

Gene ID	Symbol	Description	DEG/Set count	2mWT_2mKO	20mWT_20mKO	2mKO_20mKO	2mWT_20mWT	2mWT_30mWT	20mWT_30mWT	Literature-Mining (Aging-Cholesterol literature)		
				9	230	406	48	1904	1157	#Paper	Enrichment	BH P-value
320982	Arl4c	ADP-ribosylation factor-like 4C	1						2.07			
17095	Lyl1	lymphoblastomic leukemia 1	1						2.07			
13197	Gadd45a	growth arrest and DNA-damage-inducible 45 alpha	1						2.06			
18627	Per2	period homolog 2 (Drosophila)	1						2.01			
74481	Batf2	basic leucine zipper transcription factor, ATF-like 2	1						1.97			
12904	Crabp2	cellular retinoic acid binding protein II	1						1.97			
100169874	Gm11110	predicted gene 11110	1						1.96			
12578	Cdkn2a	cyclin-dependent kinase inhibitor 2A	1						1.94	4	1.5	0.41
102626	Mapkapk3	mitogen-activated protein kinase-activated protein kinase 3	1						1.94			
14159	Fes	feline sarcoma oncogene	1						1.94			
12982	Csf2ra	colony stimulating factor 2 receptor, alpha, low-affinity (granulocyte-macrophage)	1						1.92			
237887	Slfn10-ps	schlafen 10, pseudogene	1						1.91			
67238	2810453I06Rik	RIKEN cDNA 2810453I06 gene	1						1.90			
85031	Pla1a	phospholipase A1 member A	1						1.90			
215387	Ncaph	non-SMC condensin I complex, subunit H	1						1.88			
107995	Cdc20	cell division cycle 20 homolog (S. cerevisiae)	1						1.87			
224109	Lrrc33	leucine rich repeat containing 33	1						1.86			
16534	Kcnn4	potassium intermediate/small conductance calcium-activated channel, subfamily N, member 4	1						1.86			
18491	Pappa	pregnancy-associated plasma protein A	1						1.84			
72514	Fgfbp3	fibroblast growth factor binding protein 3	1						1.83			
212937	Tifab	TRAF-interacting protein with forkhead-associated domain, family member B	1						1.83			
54199	Ccrl2	chemokine (C-C motif) receptor-like 2	1						1.80			
74190	1200009I06Rik	RIKEN cDNA 1200009I06 gene	1						1.79			
19277	Ptpro	protein tyrosine phosphatase, receptor type, O	1						1.77			
80986	Ckap2	cytoskeleton associated protein 2	1						1.76			
74048	4632428N05Rik	RIKEN cDNA 4632428N05 gene	1						1.74			
319772	C130050O18Rik	RIKEN cDNA C130050O18 gene	1						1.67			
24014	Rnasel	ribonuclease L (2', 5'-oligoisoadenylate synthetase-dependent)	1						1.66			
106795	Tcf19	transcription factor 19	1						1.64			
211770	Trib1	tribbles homolog 1 (Drosophila)	1						1.63			
72461	Prcp	prolylcarboxypeptidase (angiotensinase C)	1						1.63			
66889	Rnf128	ring finger protein 128	1						1.63			
50706	Postn	periostin, osteoblast specific factor	1						1.63	1	10.2	0.17
217835	Rin3	Ras and Rab interactor 3	1						1.62			
270084	Lpcat2	lysophosphatidylcholine acyltransferase 2	1						1.60			
229541	Dennd4b	DENN/MADD domain containing 4B	1						1.59			
19246	Ptpn1	protein tyrosine phosphatase, non-receptor type 1	1						1.59	2	7.7	0.07
12978	Csf1r	colony stimulating factor 1 receptor	1						1.58			
21906	Otop1	otopetrin 1	1						1.58			
20111	Rps6ka1	ribosomal protein S6 kinase polypeptide 1	1						1.58			
20448	St6galnac4	ST6 (alpha-N-acetyl-neuraminyl-2,3-beta-galactosyl-1,3)-N-acetylgalactosaminide alpha-2,6-sialyltransferase 4	1						1.57			
67951	Tubb6	tubulin, beta 6 class V	1						1.56			
71801	Plekhf2	pleckstrin homology domain containing, family F (with FYVE domain) member 2	1						1.56			
12489	Cd33	CD33 antigen	1						1.56	1	1.9	0.46
56427	Tubd1	tubulin, delta 1	1						1.56			
216881	Wscd1	WSC domain containing 1	1						1.56			
12495	Entpd1	ectonucleoside triphosphate diphosphohydrolase 1	1						1.56			
12822	Col18a1	collagen, type XVIII, alpha 1	1						1.55			
74153	Uba7	ubiquitin-like modifier activating enzyme 7	1						1.54			
208104	Mlxip	MLX interacting protein	1						1.54			
69136	Tusc1	tumor suppressor candidate 1	1						1.53			
14296	Frat1	frequently rearranged in advanced T-cell lymphomas	1						1.53			
72535	Aldh1b1	aldehyde dehydrogenase 1 family, member B1	1						1.50			
17242	Mdk	midkine	1						1.50			
110173	Manba	mannosidase, beta A, lysosomal	1						1.47			
230279	6330416G13Rik	RIKEN cDNA 6330416G13 gene	1						1.47			
75430	3200002M19Rik	RIKEN cDNA 3200002M19 gene	1						1.47			
18074	Nid2	nidogen 2	1						1.47			
19735	Rgs2	regulator of G-protein signaling 2	1						1.46			
74008	Arsg	arylsulfatase G	1						1.46			
14739	S1pr2	sphingosine-1-phosphate receptor 2	1						1.46			
217069	Trim25	tripartite motif-containing 25	1						1.45			
56473	Fads2	fatty acid desaturase 2	1						1.45	7	60.6	8.39E-10
58223	Mmp19	matrix metallopeptidase 19	1						1.44	1	39.7	0.07
27419	Naglu	alpha-N-acetylglucosaminidase (Sanfilippo disease IIIB)	1						1.44	1	5.7	0.23
69537	Dnase1l1	deoxyribonuclease 1-like 1	1						1.44			
17285	Meox1	mesenchyme homeobox 1	1						1.43			
72238	Tbc1d5	TBC1 domain family, member 5	1						1.43			
192656	Ripk2	receptor (TNFRSF)-interacting serine-threonine kinase 2	1						1.42			

Gene ID	Symbol	Description	DEG/Set count	2mWT_2mKO	20mWT_20mKO	2mKO_20mKO	2mWT_20mWT	2mWT_30mWT	20mWT_30mWT	Literature-Mining (Aging-Cholesterol literature)		
				9	230	406	48	1904	1157	#Paper	Enrichment	BH P-value
66164	Nip7	nuclear import 7 homolog (S. cerevisiae)	1						1.42			
19025	Ctsa	cathepsin A	1						1.41			
14806	Grik2	glutamate receptor, ionotropic, kainate 2 (beta 2)	1						1.41			
12266	C3	complement component 3	1						1.41			
56212	Rhog	ras homolog gene family, member G	1						1.41			
13435	Dnmt3a	DNA methyltransferase 3A	1						1.40			
11482	Acvrl1	activin A receptor, type II-like 1	1						1.39	1	5	0.26
15976	Ifnar2	interferon (alpha and beta) receptor 2	1						1.39			
21745	Tep1	telomerase associated protein 1	1						1.38			
102657	Cd276	CD276 antigen	1						1.38			
381903	Alg8	asparagine-linked glycosylation 8 homolog (yeast, alpha-1,3-glucosyltransferase)	1						1.37			
214855	Arid5a	AT rich interactive domain 5A (MRF1-like)	1						1.37			
98711	Rdh10	retinol dehydrogenase 10 (all-trans)	1						1.35			
70021	Nt5dc2	5'-nucleotidase domain containing 2	1						1.34			
225348	Wdr36	WD repeat domain 36	1						1.33			
75219	Dusp18	dual specificity phosphatase 18	1						1.31			
382639	Zbtb42	zinc finger and BTB domain containing 42	1						1.29			
78339	Ttyh3	tweety homolog 3 (Drosophila)	1						1.29			
23789	Coro1b	coronin, actin binding protein 1B	1						1.29			
72400	Pinx1	PIN2/TERF1 interacting, telomerase inhibitor 1	1						1.29			
14791	Emg1	EMG1 nucleolar protein homolog (S. cerevisiae)	1						1.28			
22042	Tfrc	transferrin receptor	1						1.28	2	1.5	0.46
20529	Slc31a1	solute carrier family 31, member 1	1						1.28			
15374	Hn1	hematological and neurological expressed sequence 1	1						1.27			
12496	Entpd2	ectonucleoside triphosphate diphosphohydrolase 2	1						1.27			
59126	Nek6	NIMA (never in mitosis gene a)-related expressed kinase 6	1						1.26			
18830	Pltp	phospholipid transfer protein	1						1.26	9	78.9	1.71E-13
54357	Epb4.1l4b	erythrocyte protein band 4.1-like 4b	1						1.26			
57344	As3mt	arsenic (+3 oxidation state) methyltransferase	1						1.25			
68079	Pdcd2l	programmed cell death 2-like	1						1.25			
21853	Timeless	timeless homolog (Drosophila)	1						1.24			
57912	Cdc42se1	CDC42 small effector 1	1						1.24			
66054	Cndp2	CNDP dipeptidase 2 (metallopeptidase M20 family)	1						1.23			
56187	Rabggta	Rab geranylgeranyl transferase, a subunit	1						1.22			
26987	Eif4e2	eukaryotic translation initiation factor 4E member 2	1						1.22			
21848	Trim24	tripartite motif-containing 24	1						1.21			
107045	Lars	leucyl-tRNA synthetase	1						1.19			
14466	Gba	glucosidase, beta, acid	1						1.18			
67443	Map1lc3b	microtubule-associated protein 1 light chain 3 beta	1						-1.12			
68024	Hist1h2bc	histone cluster 1, H2bc	1						-1.14			
68943	Pink1	PTEN induced putative kinase 1	1						-1.14			
12955	Cryab	crystallin, alpha B	1						-1.14			
13602	Sparcl1	SPARC-like 1	1						-1.15			
56044	Rala	v-ral simian leukemia viral oncogene homolog A (ras related)	1						-1.16			
11518	Add1	adducin 1 (alpha)	1						-1.16			
102866	Pls3	plastin 3 (T-isoform)	1						-1.16			
53328	Pgrmc1	progesterone receptor membrane component 1	1						-1.16	1	10.7	0.16
20742	Spnb2	spectrin beta 2	1						-1.17			
19247	Ptpn11	protein tyrosine phosphatase, non-receptor type 11	1						-1.18			
215114	Hip1	huntingtin interacting protein 1	1						-1.19			
432467	Hnrnph3	heterogeneous nuclear ribonucleoprotein H3	1						-1.19			
12741	Cldn5	claudin 5	1						-1.20			
18027	Nfia	nuclear factor I/A	1						-1.20			
17988	Ndrg1	N-myc downstream regulated gene 1	1						-1.21			
15511	Hspa1b	heat shock protein 1B	1						-1.22			
16573	Kif5b	kinesin family member 5B	1						-1.23	1	8.2	0.19
54189	Rabep1	rabaptin, RAB GTPase binding effector protein 1	1						-1.23			
226352	Epb4.1l5	erythrocyte protein band 4.1-like 5	1						-1.23			
56248	Ak3	adenylate kinase 3	1						-1.23			
19285	Ptrf	polymerase I and transcript release factor	1						-1.23			
63985	Gmfb	glia maturation factor, beta	1						-1.23			
26400	Map2k7	mitogen-activated protein kinase kinase 7	1						-1.23			
22026	Nr2c2	nuclear receptor subfamily 2, group C, member 2	1						-1.23			
20362	Sept8	septin 8	1						-1.23			
207375	Fam120c	family with sequence similarity 120, member C	1						-1.24			
73569	Vgll3	vestigial like 3 (Drosophila)	1						-1.24			
15529	Sdc2	syndecan 2	1						-1.25			
319845	Bbs9	Bardet-Biedl syndrome 9 (human)	1						-1.25	1	7.8	0.19
67075	Magt1	magnesium transporter 1	1						-1.25			
77976	Nuak1	NUAK family, SNF1-like kinase, 1	1						-1.25			
74159	Acbd5	acyl-Coenzyme A binding domain containing 5	1						-1.25			
22253	Unc5c	unc-5 homolog C (C. elegans)	1						-1.26			
16974	Lrp6	low density lipoprotein receptor-related protein 6	1						-1.26			
24017	Rnf13	ring finger protein 13	1						-1.26			

Gene ID	Symbol	Description	DEG/Set count	2mWT_2mKO	20mWT_20mKO	2mKO_20mKO	2mWT_20mWT	2mWT_30mWT	20mWT_30mWT	Literature-Mining (Aging-Cholesterol literature)		
				9	230	406	48	1904	1157	#Paper	Enrichment	BH P-value
11490	Adam15	a disintegrin and metallopeptidase domain 15 (metargidin)	1						-1.26			
68121	Cep70	centrosomal protein 70	1						-1.27			
22720	Zfp62	zinc finger protein 62	1						-1.27			
13591	Ebf1	early B-cell factor 1	1						-1.27			
72145	Wdfy3	WD repeat and FYVE domain containing 3	1						-1.27			
50781	Dkk3	dickkopf homolog 3 (Xenopus laevis)	1						-1.27	1	29.2	0.08
21872	Tjp1	tight junction protein 1	1						-1.27	1	1.8	0.48
71069	Stox2	storkhead box 2	1						-1.27			
53814	Oaz3	ornithine decarboxylase antizyme 3	1						-1.27			
12305	Ddr1	discoidin domain receptor family, member 1	1						-1.28			
56274	Stk3	serine/threonine kinase 3 (Ste20, yeast homolog)	1						-1.28			
227399	Ppip5k2	diphosphoinositol pentakisphosphate kinase 2	1						-1.28			
114713	Rasa2	RAS p21 protein activator 2	1						-1.28			
18139	Zfml	zinc finger, matrin-like	1						-1.28			
270035	Letm2	leucine zipper-EF-hand containing transmembrane protein 2	1						-1.28			
106931	Kctd1	potassium channel tetramerisation domain containing 1	1						-1.29			
229542	Gatad2b	GATA zinc finger domain containing 2B	1						-1.29			
17311	Kitl	kit ligand	1						-1.29	1	0.6	1.01
227737	Fam129b	family with sequence similarity 129, member B	1						-1.29			
78619	Zfp449	zinc finger protein 449	1						-1.29			
270627	Taf1	TAF1 RNA polymerase II, TATA box binding protein (TBP)-associated factor	1						-1.30			
319922	Vwc2	von Willebrand factor C domain containing 2	1						-1.30			
71389	Chd6	chromodomain helicase DNA binding protein 6	1						-1.30			
75415	Arhgap12	Rho GTPase activating protein 12	1						-1.30			
19317	Qk	quaking	1						-1.31			
109624	Cald1	caldesmon 1	1						-1.31			
215789	Phactr2	phosphatase and actin regulator 2	1						-1.31			
99929	Tiparp	TCDD-inducible poly(ADP-ribose) polymerase	1						-1.31	1	2.6	0.39
232813	Shisa7	shisa homolog 7 (Xenopus laevis)	1						-1.32			
14265	Fmr1	fragile X mental retardation syndrome 1 homolog	1						-1.33			
13844	Ephb2	Eph receptor B2	1						-1.33			
76594	Dnajc18	DnaJ (Hsp40) homolog, subfamily C, member 18	1						-1.34			
320234	Ccdc66	coiled-coil domain containing 66	1						-1.34			
54678	Zfp108	zinc finger protein 108	1						-1.34			
218214	Kdm1b	lysine (K)-specific demethylase 1B	1						-1.35			
23797	Akt3	thymoma viral proto-oncogene 3	1						-1.35			
97440	B3gnt9-ps	UDP-GlcNAc:betaGal beta-1,3-N-acetylglucosaminyltransferase 9, pseudogene	1						-1.36			
67420	Far1	fatty acyl CoA reductase 1	1						-1.36			
19659	Rbp1	retinol binding protein 1, cellular	1						-1.36			
57276	Vsig2	V-set and immunoglobulin domain containing 2	1						-1.36			
76453	Prss23	protease, serine, 23	1						-1.37			
72599	Pdia5	protein disulfide isomerase associated 5	1						-1.37			
69601	Dab2ip	disabled homolog 2 (Drosophila) interacting protein	1						-1.37			
16779	Lamb2	laminin, beta 2	1						-1.37			
329152	Hecw2	HECT, C2 and WW domain containing E3 ubiquitin protein ligase 2	1						-1.37			
233863	Gtf3c1	general transcription factor III C 1	1						-1.37			
22141	Tub	tubby candidate gene	1						-1.38			
83486	Rbm5	RNA binding motif protein 5	1						-1.38			
11819	Nr2f2	nuclear receptor subfamily 2, group F, member 2	1						-1.38			
16011	Igfbp5	insulin-like growth factor binding protein 5	1						-1.38			
18008	Nes	nestin	1						-1.38	1	1.5	0.55
100503827	2010012P19Rik	RIKEN cDNA 2010012P19 gene	1						-1.39			
230959	Ajap1	adherens junction associated protein 1	1						-1.39			
12628	Cfh	complement component factor h	1						-1.40	2	4	0.16
18798	Plcb4	phospholipase C, beta 4	1						-1.40			
108897	Aif1l	allograft inflammatory factor 1-like	1						-1.41			
15424	Hoxc5	homeobox C5	1						-1.42			
18654	Pgf	placental growth factor	1						-1.43	1	2.6	0.39
73086	Rps6ka5	ribosomal protein S6 kinase, polypeptide 5	1						-1.43			
72656	Ints8	integrator complex subunit 8	1						-1.43			
381157	Greb1l	growth regulation by estrogen in breast cancer-like	1						-1.44			
71163	Zfp626	zinc finger protein 626	1						-1.44			
380928	Lmo7	LIM domain only 7	1						-1.44			
26568	Slc27a3	solute carrier family 27 (fatty acid transporter), member 3	1						-1.44			
14239	Foxs1	forkhead box S1	1						-1.44			
24001	Tiam2	T-cell lymphoma invasion and metastasis 2	1						-1.47			
16597	Klf12	Kruppel-like factor 12	1						-1.47			
227545	5430407P10Rik	RIKEN cDNA 5430407P10 gene	1						-1.47			
71355	Col24a1	collagen, type XXIV, alpha 1	1						-1.48			
69551	2310022B05Rik	RIKEN cDNA 2310022B05 gene	1						-1.49			
67573	Loxl4	lysyl oxidase-like 4	1						-1.49			
107250	Kazald1	Kazal-type serine peptidase inhibitor domain 1	1						-1.50			

Gene ID	Symbol	Description	DEG/Set count	2mWT_2mKO	20mWT_20mKO	2mKO_20mKO	2mWT_20mWT	2mWT_30mWT	20mWT_30mWT	Literature-Mining (Aging-Cholesterol literature)		
				9	230	406	48	1904	1157	#Paper	Enrichment	BH P-value
170761	Pdzd3	PDZ domain containing 3	1						-1.50			
329739	Fam102b	family with sequence similarity 102, member B	1						-1.51			
100504017	LOC100504017	uncharacterized LOC100504017	1						-1.52			
207596	Thsd4	thrombospondin, type I, domain containing 4	1						-1.52			
66175	Mustn1	musculoskeletal, embryonic nuclear protein 1	1						-1.52			
80883	Ntng1	netrin G1	1						-1.52			
213556	Plekhh2	pleckstrin homology domain containing, family H (with MyTH4 domain) member 2	1						-1.55			
14234	Foxc2	forkhead box C2	1						-1.55			
233071	Arhgap33	Rho GTPase activating protein 33	1						-1.56			
19041	Ppl	periplakin	1						-1.56			
68097	Dynll2	dynein light chain LC8-type 2	1						-1.57			
13592	Ebf2	early B-cell factor 2	1						-1.58			
13616	Edn3	endothelin 3	1						-1.58	1	2.4	0.41
109135	Plekha5	pleckstrin homology domain containing, family A member 5	1						-1.59			
18049	Ngf	nerve growth factor	1						-1.60	5	1.5	0.46
234365	Yjefn3	YjeF N-terminal domain containing 3	1						-1.60			
216166	Plk5	polo-like kinase 5 (Drosophila)	1						-1.61			
217480	Dgkb	diacylglycerol kinase, beta	1						-1.63			
72168	Aifm3	apoptosis-inducing factor, mitochondrion-associated 3	1						-1.63			
218343	Ttc37	tetratricopeptide repeat domain 37	1						-1.64			
69479	1700029J07Rik	RIKEN cDNA 1700029J07 gene	1						-1.64			
279653	Pcdh19	protocadherin 19	1						-1.65			
75571	Spata9	spermatogenesis associated 9	1						-1.65			
15165	Hcn1	hyperpolarization-activated, cyclic nucleotide-gated K+ 1	1						-1.66			
243659	Styk1	serine/threonine/tyrosine kinase 1	1						-1.67			
12577	Cdkn1c	cyclin-dependent kinase inhibitor 1C (P57)	1						-1.68			
98845	Eps8l2	EPS8-like 2	1						-1.72			
105387	Akr1c14	aldo-keto reductase family 1, member C14	1						-1.72			
219149	Xkr6	X Kell blood group precursor related family member 6 homolog	1						-1.72			
67866	Wfdc1	WAP four-disulfide core domain 1	1						-1.73			
30785	Cttnbp2	cortactin binding protein 2	1						-1.74			
320878	Mical2	microtubule associated monoxygenase, calponin and LIM domain containing 2	1						-1.75			
71406	5430416O09Rik	RIKEN cDNA 5430416O09 gene	1						-1.76			
103841	Cuedc1	CUE domain containing 1	1						-1.76			
396184	Flrt1	fibronectin leucine rich transmembrane protein 1	1						-1.76			
213019	Pdlim2	PDZ and LIM domain 2	1						-1.80			
70546	Zdhhc2	zinc finger, DHHC domain containing 2	1						-1.82			
97848	Serpinb6c	serine (or cysteine) peptidase inhibitor, clade B, member 6c	1						-1.86			
232599	Gm4876	predicted gene 4876	1						-1.90			
24084	Tekt2	tektin 2	1						-1.91			
76886	Fam81a	family with sequence similarity 81, member A	1						-1.94			
14428	Galr2	galanin receptor 2	1						-1.95			
110310	Krt7	keratin 7	1						-1.95			
71897	Lypd6b	LY6/PLAUR domain containing 6B	1						-2.00			
21954	Tnni3	troponin I, cardiac 3	1						-2.03			
13168	Dbil5	diazepam binding inhibitor-like 5	1						-2.25			
17898	Myl7	myosin, light polypeptide 7, regulatory	1						-2.29			
11807	Apoa2	apolipoprotein A-II	1						-2.49	22	253.9	3.59E-43
18979	Pon1	paraoxonase 1	1					-3.79		29	57.3	9.62E-39
13070	Cyp11a1	cytochrome P450, family 11, subfamily a, polypeptide 1	1		1.54					23	66.7	2.62E-32
22329	Vcam1	vascular cell adhesion molecule 1	1					2.37		12	6.7	4.25E-06
11815	Apod	apolipoprotein D	1					1.17		4	54.1	1.21E-05
22041	Trf	transferrin	1					1.55		16	4	4.30E-05
17187	Max	Max protein	1					1.51		3	54.7	2.15E-04
231162	Cytl1	cytokine-like 1	1					2.00		3	51.5	2.51E-04
11461	Actb	actin, beta	1					1.46		8	6.8	2.58E-04
15925	Ide	insulin degrading enzyme	1					1.77		3	24.1	1.86E-03
110460	Acat2	acetyl-Coenzyme A acetyltransferase 2	1					-1.30		2	75	2.20E-03
64383	Sirt2	sirtuin 2 (silent mating type information regulation 2, homolog) 2 (S. cerevisiae)	1					-1.15		3	19.6	3.18E-03
72061	2010111I01Rik	RIKEN cDNA 2010111I01 gene	1					1.56		2	58	3.45E-03
14580	Gfap	glial fibrillary acidic protein	1					6.59		10	3.6	3.76E-03
16987	Lss	lanosterol synthase	1					-2.75		2	45.6	0.01
21898	Tlr4	toll-like receptor 4	1					1.53		7	4.4	0.01
22122	Tsta3	tissue specific transplantation antigen P35B	1					1.25		1	680.9	0.01
13010	Cst3	cystatin C	1					1.14		4	7.7	0.01
17855	Mvk	mevalonate kinase	1					-1.43		2	27	0.01
16905	Lmna	lamin A	1					1.15		3	9.9	0.02
56742	Psrc1	proline/serine-rich coiled-coil 1	1					3.59		1	194.6	0.02
14812	Grin2b	glutamate receptor, ionotropic, NMDA2B (epsilon 2)	1					-1.64		3	7.8	0.03
20753	Sprr1a	small proline-rich protein 1A	1					3.75		1	131.8	0.03
20619	Snap23	synaptosomal-associated protein 23	1					1.81		2	14.4	0.03
54326	Elovl2	elongation of very long chain fatty acids (FEN1/Elo2, SUR4/Elo3, yeast)-like 2	1		-2.35					1	107.5	0.03

Gene ID	Symbol	Description	DEG/Set count	2mWT_2mKO	20mWT_20mKO	2mKO_20mKO	2mWT_20mWT	2mWT_30mWT	20mWT_30mWT	Literature-Mining (Aging-Cholesterol literature)		
				9	230	406	48	1904	1157	#Paper	Enrichment	BH P-value
330064	Slc5a6	solute carrier family 5 (sodium-dependent vitamin transporter), member 6	1		1.42					1	104.8	0.03
19655	Rbmx	RNA binding motif protein, X chromosome	1					-1.28		1	80.1	0.04
20657	Sod3	superoxide dismutase 3, extracellular	1					-1.36		2	11	0.05
14751	Gpi1	glucose phosphate isomerase 1	1					1.25		4	4	0.06
15108	Hsd17b10	hydroxysteroid (17-beta) dehydrogenase 10	1					-1.18		1	48.1	0.06
26413	Mapk1	mitogen-activated protein kinase 1	1					1.42		19	1.7	0.06
12827	Col4a2	collagen, type IV, alpha 2	1					-1.32		1	44.4	0.06
18791	Plat	plasminogen activator, tissue	1					1.39		9	2.2	0.07
13139	Dgka	diacylglycerol kinase, alpha	1					-1.29		1	37.1	0.07
50798	Gne	glucosamine	1					-1.47		1	31	0.08
59056	Evc	Ellis van Creveld gene homolog (human)	1					-1.47		1	25.4	0.09
105787	Prkaa1	protein kinase, AMP-activated, alpha 1 catalytic subunit	1					2.03		3	4.1	0.09
17295	Met	met proto-oncogene	1	-1.40						6	2.4	0.09
18618	Pemt	phosphatidylethanolamine N-methyltransferase	1					-1.35		1	22.4	0.10
65969	Cubn	cubilin (intrinsic factor-cobalamin receptor)	1					-1.35		1	19.3	0.11
17873	Gadd45b	growth arrest and DNA-damage-inducible 45 beta	1					1.52		1	14.9	0.13
12476	Cd151	CD151 antigen	1					-1.14		1	14.5	0.14
232345	A2m	alpha-2-macroglobulin	1					1.47		3	3.1	0.15
19288	Ptx3	pentraxin related gene	1					1.82		1	12.5	0.15
15213	Hey1	hairy/enhancer-of-split related with YRPW motif 1	1					1.82		1	11.7	0.15
13875	Erf	Ets2 repressor factor	1					1.40		1	11	0.16
217944	Rapgef5	Rap guanine nucleotide exchange factor (GEF) 5	1		-1.61					1	10.7	0.16
12490	Cd34	CD34 antigen	1					-1.35		1	0.2	0.17
109672	Cyb5	cytochrome b-5	1					-1.16		2	3.7	0.18
15007	H2-Q10	histocompatibility 2, Q region locus 10	1		3.44					3	0.4	0.18
20544	Slc9a1	solute carrier family 9 (sodium/hydrogen exchanger), member 1	1					-1.36		1	8.3	0.19
12309	S100g	S100 calcium binding protein G	1					-1.74		1	7.7	0.19
16391	Irf9	interferon regulatory factor 9	1					1.58		1	7.4	0.20
27973	Vkorc1	vitamin K epoxide reductase complex, subunit 1	1					-1.22		1	7.1	0.20
13019	Ctf1	cardiotrophin 1	1					-1.37		1	5.9	0.23
20869	Stk11	serine/threonine kinase 11	1					1.32		1	5.6	0.24
22323	Vasp	vasodilator-stimulated phosphoprotein	1					1.82		1	5.6	0.24
12868	Cox8a	cytochrome c oxidase, subunit VIIIa	1					1.23		4	1.9	0.24
67126	Atp5e	ATP synthase, H+ transporting, mitochondrial F1 complex, epsilon subunit	1					1.10		1	5.3	0.25
16728	L1cam	L1 cell adhesion molecule	1					1.59		1	5	0.26
15901	Id1	inhibitor of DNA binding 1	1					1.33		1	4.9	0.26
15528	Hspe1	heat shock protein 1 (chaperonin 10)	1					1.61		1	4.6	0.27
12028	Bax	BCL2-associated X protein	1					1.37		1	0.3	0.35
21808	Tgfb2	transforming growth factor, beta 2	1					1.36		1	2.5	0.40
12995	Csnk2a1	casein kinase 2, alpha 1 polypeptide	1					1.30		1	2.3	0.42
13555	E2f1	E2F transcription factor 1	1					1.47		1	1.8	0.48
50873	Park2	Parkinson disease (autosomal recessive, juvenile) 2, parkin	1					-1.36		1	1.7	0.51
19229	Ptk2b	PTK2 protein tyrosine kinase 2 beta	1					1.46		1	1.5	0.55
14573	Gdnf	glial cell line derived neurotrophic factor	1					4.15		1	1.3	0.58
15452	Hprt	hypoxanthine guanine phosphoribosyl transferase	1					1.57		1	1.3	0.59
18591	Pdgfb	platelet derived growth factor, B polypeptide	1					1.45		4	1.2	0.63
15567	Slc6a4	solute carrier family 6 (neurotransmitter transporter, serotonin), member 4	1		2.77					1	1.1	0.65
12370	Casp8	caspase 8	1					2.63		1	0.4	0.78
109648	Npy	neuropeptide Y	1					3.60		3	1.2	0.78
13361	Dhfr	dihydrofolate reductase	1					-1.36		1	0.8	1.01
52696	Zwint	ZW10 interactor	1					1.53				
229937	Znhit6	zinc finger, HIT type 6	1					-1.18				
100177	Zmym6	zinc finger, MYM-type 6	1					-1.31				
67785	Zmym4	zinc finger, MYM-type 4	1					-1.26				
74149	Zfp946	zinc finger protein 946	1					1.84				
382019	Zfp882	zinc finger protein 882	1					-1.33				
408067	Zfp874b	zinc finger protein 874b	1					-1.40				
244216	Zfp771	zinc finger protein 771	1					-1.27				
233893	Zfp764	zinc finger protein 764	1					-1.48				
353310	Zfp703	zinc finger protein 703	1					1.25				
22704	Zfp46	zinc finger protein 46	1					-1.29				
22701	Zfp41	zinc finger protein 41	1					-1.41				
57247	Zfp276	zinc finger protein (C2H2 type) 276	1					-1.35				
232784	Zfp212	Zinc finger protein 212	1					-1.26				
22680	Zfp207	zinc finger protein 207	1					1.83				
230162	Zfp189	zinc finger protein 189	1					1.36				
72154	Zfp157	zinc finger protein 157	1					-1.31				
26465	Zfp146	zinc finger protein 146	1					1.47				
243906	Zfp14	zinc finger protein 14	1					-1.51				
27801	Zdhhc8	zinc finger, DHHC domain containing 8	1					-1.44				
72881	Zdhhc4	zinc finger, DHHC domain containing 4	1					-1.31				
69035	Zdhhc3	zinc finger, DHHC domain containing 3	1					-1.23				
20286	Zc3h7b	zinc finger CCCH type containing 7B	1					-1.30				
237256	Zc3h12d	zinc finger CCCH type containing 12D	1					7.43				

Gene ID	Symbol	Description	DEG/Set count	2mWT_2mKO	20mWT_20mKO	2mKO_20mKO	2mWT_20mWT	2mWT_30mWT	20mWT_30mWT	Literature-Mining (Aging-Cholesterol literature)		
				9	230	406	48	1904	1157	#Paper	Enrichment	BH P-value
22627	Ywhae	tyrosine 3-monooxygenase/tryptophan 5-monooxygenase activation protein, epsilon polypeptide	1					-1.12				
229096	Ythdf3	YTH domain family 3	1					1.28				
213541	Ythdf2	YTH domain family 2	1					1.31				
66090	Ypel3	yippee-like 3 (Drosophila)	1					-1.25				
77254	Yif1b	Yip1 interacting factor homolog B (S. cerevisiae)	1					-1.32				
170745	Xpnpep2	X-prolyl aminopeptidase (aminopeptidase P) 2, membrane-bound	1					-2.00				
211652	Wwc1	WW, C2 and coiled-coil domain containing 1	1					-1.89				
68151	Wls	wntless homolog (Drosophila)	1					1.38				
215280	Wipf1	WAS/WASL interacting protein family, member 1	1					1.58				
269633	Wdr86	WD repeat domain 86	1					-1.66				
54636	Wdr45	WD repeat domain 45	1					-1.31				
218460	Wdr41	WD repeat domain 41	1					1.48				
244484	Wdr17	WD repeat domain 17	1					-1.39				
22381	Wbp5	WW domain binding protein 5	1					1.68				
233813	Vwa3a	von Willebrand factor A domain containing 3A	1					-1.60				
26950	Vsnl1	visinin-like 1	1		-3.10							
28084	Vps25	vacuolar protein sorting 25 (yeast)	1					1.28				
22361	Vnn1	vanin 1	1					-2.10				
74199	Vit	vitrin	1					-2.25				
53620	Vamp5	vesicle-associated membrane protein 5	1					-1.47				
252870	Usp7	ubiquitin specific peptidase 7	1					1.23				
78787	Usp54	ubiquitin specific peptidase 54	1					-1.50				
74841	Usp38	ubiquitin specific peptidase 38	1					1.65				
170822	Usp33	ubiquitin specific peptidase 33	1					1.48				
30940	Usp25	ubiquitin specific peptidase 25	1					1.36				
14479	Usp15	ubiquitin specific peptidase 15	1					1.34				
234395	Ushbp1	Usher syndrome 1C binding protein 1	1					-1.81				
67023	Use1	unconventional SNARE in the ER 1 homolog (S. cerevisiae)	1					1.24				
22249	Unc13b	unc-13 homolog B (C. elegans)	1					-1.63				
382018	Unc13a	unc-13 homolog A (C. elegans)	1		-1.70							
106840	Unc119b	unc-119 homolog B (C. elegans)	1					-1.26				
75089	Uhrf1bp1l	UHRF1 (ICBP90) binding protein 1-like	1					1.37				
66155	Ufc1	ubiquitin-fold modifier conjugating enzyme 1	1					1.24				
212190	Ubxn10	UBX domain protein 10	1					-1.60				
68795	Ubr3	ubiquitin protein ligase E3 component n-recognin 3	1					1.31				
217342	Ube2o	ubiquitin-conjugating enzyme E2O	1					1.75				
93765	Ube2n	ubiquitin-conjugating enzyme E2N	1		-1.62							
56791	Ube2l6	ubiquitin-conjugating enzyme E2L 6	1					7.81				
56550	Ube2d2	ubiquitin-conjugating enzyme E2D 2	1		-1.16							
50493	Txnrd1	thioredoxin reductase 1	1					1.29				
52700	Txndc17	thioredoxin domain containing 17	1					1.13				
22160	Twist1	twist homolog 1 (Drosophila)	1					-1.53				
68842	Tulp4	tubby like protein 4	1					1.32				
74711	Ttll9	tubulin tyrosine ligase-like family, member 9	1					-1.54				
67515	Ttc33	tetratricopeptide repeat domain 33	1					1.43				
56496	Tspan6	tetraspanin 6	1					-1.45				
74257	Tspan17	tetraspanin 17	1					-1.37				
78829	Tsc22d4	TSC22 domain family, member 4	1					-1.29				
72033	Tsc22d2	TSC22 domain family, member 2	1					1.30				
22368	Trpv2	transient receptor potential cation channel, subfamily V, member 2	1					1.93				
28240	Trpm2	transient receptor potential cation channel, subfamily M, member 2	1					1.48				
22065	Trpc3	transient receptor potential cation channel, subfamily C, member 3	1		-1.94							
229644	Trim45	tripartite motif-containing 45	1					-1.57				
20128	Trim30a	tripartite motif-containing 30A	1					2.34				
100123473	Trdc	T-cell receptor delta, constant region	1					4.99				
245828	Trappc1	trafficking protein particle complex 1	1					1.28				
229801	Tram1l1	translocation associated membrane protein 1-like 1	1		-2.12							
22031	Traf3	TNF receptor-associated factor 3	1					1.27				
101214	Tra2a	transformer 2 alpha homolog (Drosophila)	1					1.40				
17229	Tpsb2	tryptase beta 2	1					2.49				
97031	Tprn	taperin	1					-1.31				
244579	Tox3	TOX high mobility group box family member 3	1		-2.32							
235559	Topbp1	topoisomerase (DNA) II binding protein 1	1					1.87				
28185	Tomm70a	translocase of outer mitochondrial membrane 70 homolog A (yeast)	1		1.32							
66119	Tomm6	translocase of outer mitochondrial membrane 6 homolog (yeast)	1					1.15				
231861	Tnrc18	trinucleotide repeat containing 18	1					-1.32				
665113	Tnik	TRAF2 and NCK interacting kinase	1					1.99				
21944	Tnfsf12	tumor necrosis factor (ligand) superfamily, member 12	1					-1.23				
66443	Tnfaip8l1	tumor necrosis factor, alpha-induced protein 8-like 1	1					-1.51				
387314	Tmtc1	transmembrane and tetratricopeptide repeat containing 1	1		-1.33							

Gene ID	Symbol	Description	DEG/Set count	2mWT_2mKO	20mWT_20mKO	2mKO_20mKO	2mWT_20mWT	2mWT_30mWT	20mWT_30mWT	Literature-Mining (Aging-Cholesterol literature)		
				9	230	406	48	1904	1157	#Paper	Enrichment	BH P-value
20776	Tmie	transmembrane inner ear	1					-1.40				
433485	Tmem90b	transmembrane protein 90B	1		-1.46							
66241	Tmem9	transmembrane protein 9	1					1.38				
70397	Tmem70	transmembrane protein 70	1					1.26				
96957	Tmem62	transmembrane protein 62	1					-1.43				
214359	Tmem51	transmembrane protein 51	1					1.67				
66079	Tmem42	transmembrane protein 42	1					-1.52				
68796	Tmem214	transmembrane protein 214	1					-1.36				
66320	Tmem208	transmembrane protein 208	1					1.19				
73893	Tmem202	transmembrane protein 202	1					1.58				
230917	Tmem201	transmembrane protein 201	1					-1.23				
240660	Tmem20	transmembrane protein 20	1					1.62				
83921	Tmem2	transmembrane protein 2	1					-1.25				
69094	Tmem160	transmembrane protein 160	1					-1.51				
66087	Tmem111	transmembrane protein 111	1					-1.12				
216821	Tmem11	transmembrane protein 11	1					1.40				
68212	Tmbim4	transmembrane BAX inhibitor motif containing 4	1					1.20				
69660	Tmbim1	transmembrane BAX inhibitor motif containing 1	1					-1.25				
68634	Tm2d3	TM2 domain containing 3	1					-1.23				
142980	Tlr3	toll-like receptor 3	1					1.52				
21886	Tle2	transducin-like enhancer of split 2, homolog of Drosophila E(spl)	1					-1.50				
57813	Tk2	thymidine kinase 2, mitochondrial	1					1.98				
21877	Tk1	thymidine kinase 1	1					2.24				
21855	Timm17b	translocase of inner mitochondrial membrane 17b	1					-1.44				
21854	Timm17a	translocase of inner mitochondrial membrane 17a	1					1.25				
14911	Thumpd3	THUMP domain containing 3	1					1.25				
233802	Thumpd1	THUMP domain containing 1	1					1.52				
331046	Tgm4	transglutaminase 4 (prostate)	1					-1.46				
224481	Tfb1m	transcription factor B1, mitochondrial	1					-1.27				
104271	Tex15	testis expressed gene 15	1		1.85							
21679	Tead4	TEA domain family member 4	1					-1.43				
104884	Tdp1	tyrosyl-DNA phosphodiesterase 1	1					-1.51				
102791	Tcta	T-cell leukemia translocation altered gene	1					-1.31				
21407	Tcf15	transcription factor 15	1					-2.47				
56070	Tcerg1	transcription elongation regulator 1 (CA150)	1					1.31				
21385	Tbx2	T-box 2	1					1.66				
56480	Tbk1	TANK-binding kinase 1	1					1.37				
70573	Tbccd1	TBCC domain containing 1	1					1.25				
57915	Tbc1d1	TBC1 domain family, member 1	1					1.54				
75812	Tasp1	taspase, threonine aspartase 1	1					1.74				
21353	Tank	TRAF family member-associated Nf-kappa B activator	1					1.80				
407786	Taf9b	TAF9B RNA polymerase II, TATA box binding protein (TBP)-associated factor	1					-1.23				
68776	Taf11	TAF11 RNA polymerase II, TATA box binding protein (TBP)-associated factor	1					1.25				
217031	Tada2a	transcriptional adaptor 2A	1					-1.23				
230676	Szt2	seizure threshold 2	1					-1.48				
71709	Syde1	synapse defective 1, Rho GTPase, homolog 1 (C. elegans)	1					-1.45				
72931	Swi5	SWI5 recombination repair homolog (yeast)	1					1.25				
225888	Suv420h1	suppressor of variegation 4-20 homolog 1 (Drosophila)	1					-1.27				
20937	Suv39h1	suppressor of variegation 3-9 homolog 1 (Drosophila)	1					-1.23				
20926	Supt6h	suppressor of Ty 6 homolog (S. cerevisiae)	1					1.17				
77053	Sun1	Sad1 and UNC84 domain containing 1	1					-1.20				
57429	Sult5a1	sulfotransferase family 5A, member 1	1					-1.95				
53331	Stx7	syntaxin 7	1					1.37				
58244	Stx6	syntaxin 6	1					-1.35				
20867	Stip1	stress-induced phosphoprotein 1	1					1.69				
52331	Stbd1	starch binding domain 1	1		-1.50							
99138	Stard7	START domain containing 7	1					-1.19				
243362	Stard13	StAR-related lipid transfer (START) domain containing 13	1					1.33				
217154	Stac2	SH3 and cysteine rich domain 2	1					2.67				
229681	St7l	suppression of tumorigenicity 7-like	1		1.29							
68991	Ssu72	Ssu72 RNA polymerase II CTD phosphatase homolog (yeast)	1					1.38				
14105	Srsf10	serine/arginine-rich splicing factor 10	1					1.42				
18412	Sqstm1	sequestosome 1	1					1.24				
20773	Sptlc2	serine palmitoyltransferase, long chain base subunit 2	1					-1.45				
24063	Spry1	sprouty homolog 1 (Drosophila)	1					-1.57				
20751	Spr	sepiapterin reductase	1					1.33				
66624	Spcs2	signal peptidase complex subunit 2 homolog (S. cerevisiae)	1					1.31				
71242	Spata24	spermatogenesis associated 24	1					-1.56				
216873	Spag7	sperm associated antigen 7	1					1.19				
75622	Spaca3	sperm acrosome associated 3	1					-1.46				
20668	Sox13	SRY-box containing gene 13	1		1.27							
20322	Sord	sorbitol dehydrogenase	1					-1.24				

Gene ID	Symbol	Description	DEG/Set count	2mWT_2mKO	20mWT_20mKO	2mKO_20mKO	2mWT_20mWT	2mWT_30mWT	20mWT_30mWT	Literature-Mining (Aging-Cholesterol literature)		
				9	230	406	48	1904	1157	#Paper	Enrichment	BH P-value
20410	Sorbs3	sorbin and SH3 domain containing 3	1					-1.34				
266781	Snx17	sorting nexin 17	1		1.12							
74718	Snx16	sorting nexin 16	1					1.48				
71982	Snx10	sorting nexin 10	1					1.79				
67332	Snrpd3	small nuclear ribonucleoprotein D3	1					1.34				
68981	Snrpa1	small nuclear ribonucleoprotein polypeptide A'	1					-1.19				
66618	Snrnp27	small nuclear ribonucleoprotein 27 (U4/U6.U5)	1					1.37				
66835	Snord123	small nucleolar RNA, C/D box 123	1					-1.43				
69895	Snhg8	small nucleolar RNA host gene 8	1					-1.32				
72655	Snhg5	small nucleolar RNA host gene 5	1		1.28							
56463	Snd1	staphylococcal nuclease and tudor domain containing 1	1					1.25				
20603	Sms	spermine synthase	1		-2.05							
319757	Smo	smoothened homolog (Drosophila)	1					-1.77				
20595	Smn1	survival motor neuron 1	1					1.27				
225655	Slmo1	slowmo homolog 1 (Drosophila)	1					-1.27				
20874	Slk	STE20-like kinase (yeast)	1					1.28				
240726	Slco5a1	solute carrier organic anion transporter family, member 5A1	1		-2.37							
20540	Slc7a7	solute carrier family 7 (cationic amino acid transporter, y+ system), member 7	1					1.93				
106947	Slc39a3	solute carrier family 39 (zinc transporter), member 3	1					-1.41				
68427	Slc39a13	solute carrier family 39 (metal ion transporter), member 13	1					-1.74				
72144	Slc37a3	solute carrier family 37 (glycerol-3-phosphate transporter), member 3	1					-1.32				
270066	Slc35e1	solute carrier family 35, member E1	1					-1.16				
22232	Slc35a2	solute carrier family 35 (UDP-galactose transporter), member A2	1					-1.26				
69048	Slc30a5	solute carrier family 30 (zinc transporter), member 5	1					1.40				
56017	Slc2a8	solute carrier family 2, (facilitated glucose transporter), member 8	1					-1.43				
170441	Slc2a10	solute carrier family 2 (facilitated glucose transporter), member 10	1					-1.60				
104910	Slc25a47	solute carrier family 25, member 47	1					-1.65				
73132	Slc25a16	solute carrier family 25 (mitochondrial carrier, Graves disease autoantigen), member 16	1					1.73				
238384	Slc24a4	solute carrier family 24 (sodium/potassium/calcium exchanger), member 4	1					-1.42				
73102	Slc22a23	solute carrier family 22, member 23	1					1.39				
210463	Slc22a22	solute carrier family 22 (organic cation transporter), member 22	1					-3.78				
56517	Slc22a21	solute carrier family 22 (organic cation transporter), member 21	1					-1.52				
20516	Slc20a2	solute carrier family 20, member 2	1					-1.45				
214084	Slc18a2	solute carrier family 18 (vesicular monoamine), member 2	1					1.93				
20482	Skil	SKI-like	1					1.38				
20474	Six4	sine oculis-related homeobox 4 homolog (Drosophila)	1		-1.68							
30954	Siva1	SIVA1, apoptosis-inducing factor	1					-1.25				
19261	Sirpa	signal-regulatory protein alpha	1					1.36				
20467	Sin3b	transcriptional regulator, SIN3B (yeast)	1		1.30							
268281	Shprh	SNF2 histone linker PHD RING helicase	1					-1.36				
230126	Shb	src homology 2 domain-containing transforming protein B	1		1.50							
58234	Shank3	SH3/ankyrin domain gene 3	1					-1.38				
268396	Sh3pxd2b	SH3 and PX domains 2B	1					1.41				
20408	Sh3gl3	SH3-domain GRB2-like 3	1				-1.59					
98402	Sh3bp4	SH3-domain binding protein 4	1					-1.27				
94281	Sfxn4	sideroflexin 4	1					-1.39				
94279	Sfxn2	sideroflexin 2	1					-1.50				
108735	Sft2d2	SFT2 domain containing 2	1		1.24							
20379	Sfrp4	secreted frizzled-related protein 4	1					-2.68				
353282	Sfmbt2	Scm-like with four mbt domains 2	1					-2.86				
67956	Setd8	SET domain containing (lysine methyltransferase) 8	1					-1.15				
140742	Sesn1	sestrin 1	1					-1.35				
20317	Serpinf1	serine (or cysteine) peptidase inhibitor, clade F, member 1	1					-1.44				
72661	Serp2	stress-associated endoplasmic reticulum protein family member 2	1					-1.41				
218442	Serinc5	serine incorporator 5	1					1.98				
230779	Serinc2	serine incorporator 2	1		-2.26							
378702	Serf2	small EDRK-rich factor 2	1					-1.17				
20365	Serf1	small EDRK-rich factor 1	1					-1.70				
20361	Sema7a	sema domain, immunoglobulin domain (Ig), and GPI membrane anchor, (semaphorin) 7A	1					1.31				
20357	Sema5b	sema domain, seven thrombospondin repeats (type 1 and type 1-like), transmembrane domain (TM) and short cytoplasmic domain, (semaphorin) 5B	1					-1.41				

Gene ID	Symbol	Description	DEG/Set count	2mWT_2mKO	20mWT_20mKO	2mKO_20mKO	2mWT_20mWT	2mWT_30mWT	20mWT_30mWT	Literature-Mining (Aging-Cholesterol literature)		
				9	230	406	48	1904	1157	#Paper	Enrichment	BH P-value
20354	Sema4d	sema domain, immunoglobulin domain (Ig), transmembrane domain (TM) and short cytoplasmic domain, (semaphorin) 4D	1					1.60				
27054	Sec23b	SEC23B (S. cerevisiae)	1					1.24				
317717	Sec22a	SEC22 vesicle trafficking protein homologe A (S. cerevisiae)	1					-1.32				
330222	Sdk1	sidekick homolog 1 (chicken)	1					-1.37				
53378	Sdcbp	syndecan binding protein	1					1.51				
105782	Scrib	scribbled homolog (Drosophila)	1					-1.51				
20277	Scnn1b	sodium channel, nonvoltage-gated 1 beta	1					-1.48				
399548	Scn4b	sodium channel, type IV, beta	1		-2.30							
219151	Scara3	scavenger receptor class A, member 3	1					-1.60				
319934	Sbf2	SET binding factor 2	1					-1.27				
20230	Satb1	special AT-rich sequence binding protein 1	1					-1.35				
69215	Sat2	spermidine/spermine N1-acetyl transferase 2	1					-1.30				
192166	Sardh	sarcosine dehydrogenase	1		1.43							
20220	Sap18	Sin3-associated polypeptide 18	1					-1.33				
235636	Rtp3	receptor transporter protein 3	1					-1.96				
18114	Rrp1	ribosomal RNA processing 1 homolog (S. cerevisiae)	1					1.29				
20130	Rras	Harvey rat sarcoma oncogene, subgroup R	1					-1.33				
76846	Rps9	ribosomal protein S9	1					1.36				
57294	Rps27	ribosomal protein S27	1					1.76				
20054	Rps15	ribosomal protein S15	1					-1.15				
66646	Rpe	ribulose-5-phosphate-3-epimerase	1					1.41				
66049	Rogdi	rogdi homolog (Drosophila)	1					1.32				
71673	Rnf215	ring finger protein 215	1					-1.27				
75234	Rnf19b	ring finger protein 19B	1					1.71				
59044	Rnf130	ring finger protein 130	1					-1.20				
67845	Rnf115	ring finger protein 115	1					1.37				
50849	Rnf10	ring finger protein 10	1					1.36				
56532	Ripk3	receptor-interacting serine-threonine kinase 3	1		1.41							
66878	Riok3	RIO kinase 3 (yeast)	1					1.31				
78757	Rictor	RPTOR independent companion of MTOR, complex 2	1					1.49				
66611	Ribc1	RIB43A domain with coiled-coils 1	1					-1.42				
14787	Rhpn1	rhophilin, Rho GTPase binding protein 1	1					-1.55				
59040	Rhot1	ras homolog gene family, member T1	1					-1.34				
11853	Rhoc	ras homolog gene family, member C	1					1.52				
215160	Rhbdd2	rhomboid domain containing 2	1					-1.35				
76867	Rhbdd1	rhomboid domain containing 1	1					1.28				
319758	Rfx7	regulatory factor X, 7	1					1.58				
67830	Rer1	RER1 retention in endoplasmic reticulum 1 homolog (S. cerevisiae)	1					-1.12				
140743	Rem2	rad and gem related GTP binding protein 2	1		-2.66							
70335	Reep6	receptor accessory protein 6	1					-2.02				
13476	Reep5	receptor accessory protein 5	1					-1.10				
319817	Rc3h2	ring finger and CCCH-type zinc finger domains 2	1					1.30				
207181	Rbms3	RNA binding motif, single stranded interacting protein	1		-1.69							
71684	Rbm43	RNA binding motif protein 43	1					1.31				
66810	Rbm22	RNA binding motif protein 22	1					1.45				
19651	Rbl2	retinoblastoma-like 2	1					1.60				
320292	Rasgef1b	RasGEF domain family, member 1B	1					1.98				
19385	Ranbp1	RAN binding protein 1	1					1.48				
54409	Ramp2	receptor (calcitonin) activity modifying protein 2	1					-1.58				
75646	Rai14	retinoic acid induced 14	1					-1.34				
56715	Rabgef1	RAB guanine nucleotide exchange factor (GEF) 1	1					-1.17				
224624	Rab40c	Rab40c, member RAS oncogene family	1					-1.47				
76877	Rab36	RAB36, member RAS oncogene family	1					-1.31				
19376	Rab34	RAB34, member of RAS oncogene family	1		1.47							
59021	Rab2a	RAB2A, member RAS oncogene family	1					-1.24				
19332	Rab20	RAB20, member RAS oncogene family	1					1.79				
19330	Rab18	RAB18, member RAS oncogene family	1					1.30				
72135	Pygo1	pygopus 1	1					-1.56				
218699	Pxk	PX domain containing serine/threonine kinase	1					1.54				
103136	Pwp1	PWP1 homolog (S. cerevisiae)	1					1.49				
108705	Pttg1ip	pituitary tumor-transforming 1 interacting protein	1					-1.18				
329384	Ptrh1	peptidyl-tRNA hydrolase 1 homolog (S. cerevisiae)	1					2.31				
19267	Ptpre	protein tyrosine phosphatase, receptor type, E	1					-1.24				
19262	Ptpra	protein tyrosine phosphatase, receptor type, A	1					1.32				
19250	Ptpn14	protein tyrosine phosphatase, non-receptor type 14	1					-1.33				
66461	Ptpmt1	protein tyrosine phosphatase, mitochondrial 1	1					-1.36				
218232	Ptpdc1	protein tyrosine phosphatase domain containing 1	1					-1.32				
19219	Ptger4	prostaglandin E receptor 4 (subtype EP4)	1					1.53				
19218	Ptger3	prostaglandin E receptor 3 (subtype EP3)	1		2.89							
27388	Ptdss2	phosphatidylserine synthase 2	1					-1.40				
19210	Ptdss1	phosphatidylserine synthase 1	1					-1.16				
19200	Pstpip1	proline-serine-threonine phosphatase-interacting protein 1	1					2.35				

Gene ID	Symbol	Description	DEG/Set count	2mWT_2mKO	20mWT_20mKO	2mKO_20mKO	2mWT_20mWT	2mWT_30mWT	20mWT_30mWT	Literature-Mining (Aging-Cholesterol literature)		
				9	230	406	48	1904	1157	#Paper	Enrichment	BH P-value
100678	Psph	phosphoserine phosphatase	1					1.34				
66506	Psmg3	proteasome (prosome, macropain) assembly chaperone 3	1					1.42				
228769	Psmf1	proteasome (prosome, macropain) inhibitor subunit 1	1					-1.32				
19192	Psme3	proteaseome (prosome, macropain) 28 subunit, 3	1					1.62				
19186	Psme1	proteasome (prosome, macropain) 28 subunit, alpha	1					1.49				
57296	Psmd8	proteasome (prosome, macropain) 26S subunit, non-ATPase, 8	1					1.17				
19185	Psmd4	proteasome (prosome, macropain) 26S subunit, non-ATPase, 4	1					1.42				
22123	Psmd3	proteasome (prosome, macropain) 26S subunit, non-ATPase, 3	1					1.22				
66997	Psmd12	proteasome (prosome, macropain) 26S subunit, non-ATPase, 12	1					1.28				
69077	Psmd11	proteasome (prosome, macropain) 26S subunit, non-ATPase, 11	1					1.78				
19175	Psmb6	proteasome (prosome, macropain) subunit, beta type 6	1					1.23				
19170	Psmb1	proteasome (prosome, macropain) subunit, beta type 1	1					1.14				
19153	Prx	periaxin	1					-1.18				
19142	Prss12	protease, serine, 12 neurotrypsin (motopsin)	1					-1.23				
227723	Prrc2b	proline-rich coiled-coil 2B	1					-1.16				
328099	Prps1l3	phosphoribosyl pyrophosphate synthetase 1-like 3	1					1.33				
19128	Pros1	protein S (alpha)	1					1.20				
109042	Prkcdbp	protein kinase C, delta binding protein	1					-1.56				
18753	Prkcd	protein kinase C, delta	1					1.81				
19072	Prep	prolyl endopeptidase	1					1.54				
116847	Prelp	proline arginine-rich end leucine-rich repeat	1					-1.47				
53381	Prdx4	peroxiredoxin 4	1					-1.21				
11757	Prdx3	peroxiredoxin 3	1					1.44				
77630	Prdm8	PR domain containing 8	1		-1.59							
70779	Prdm5	PR domain containing 5	1					-1.28				
114604	Prdm15	PR domain containing 15	1					-1.36				
12142	Prdm1	PR domain containing 1, with ZNF domain	1					2.86				
217430	Pqlc3	PQ loop repeat containing	1					1.72				
52036	Ppp6r3	protein phosphatase 6, regulatory subunit 3	1					1.34				
72930	Ppp2r2b	protein phosphatase 2 (formerly 2A), regulatory subunit B (PR 52), beta isoform	1		-2.51							
71978	Ppp2r2a	protein phosphatase 2 (formerly 2A), regulatory subunit B (PR 52), alpha isoform	1					1.37				
228852	Ppp1r16b	protein phosphatase 1, regulatory (inhibitor) subunit 16B	1					-1.50				
242083	Ppm1l	protein phosphatase 1 (formerly 2C)-like	1					-1.26				
14208	Ppm1g	protein phosphatase 1G (formerly 2C), magnesium-dependent, gamma isoform	1					1.24				
19035	Ppib	peptidylprolyl isomerase B	1					1.15				
217734	Pomt2	protein-O-mannosyltransferase 2	1					-1.31				
66537	Pomp	proteasome maturation protein	1					1.14				
68273	Pomgnt1	protein O-linked mannose beta1,2-N-acetylglucosaminyltransferase	1					-1.26				
69920	Polr2i	polymerase (RNA) II (DNA directed) polypeptide I	1					-1.20				
69241	Polr2d	polymerase (RNA) II (DNA directed) polypeptide D	1					1.33				
64424	Polr1e	polymerase (RNA) I polypeptide E	1					1.59				
20018	Polr1d	polymerase (RNA) I polypeptide D	1					1.21				
26447	Poli	polymerase (DNA directed), iota	1					-1.34				
69745	Pold4	polymerase (DNA-directed), delta 4	1					1.46				
80294	Pofut2	protein O-fucosyltransferase 2	1					-1.36				
242608	Podn	podocan	1					-1.89				
68603	Pmvk	phosphomevalonate kinase	1					-1.60				
102502	Pls1	plastin 1 (I-isoform)	1					2.54				
18823	Plp1	proteolipid protein (myelin) 1	1					1.30				
67801	Pllp	plasma membrane proteolipid	1					-1.25				
269608	Plekhg5	pleckstrin homology domain containing, family G (with RhoGef domain) member 5	1					-1.57				
263406	Plekhg3	pleckstrin homology domain containing, family G (with RhoGef domain) member 3	1					-1.48				
27276	Plekhb1	pleckstrin homology domain containing, family B (evectins) member 1	1					-1.17				
18807	Pld3	phospholipase D family, member 3	1					1.83				
18772	Pkp1	plakophilin 1	1					2.40				
56305	Pitpnb	phosphatidylinositol transfer protein, beta	1					-1.16				
236604	Pisd-ps1	phosphatidylserine decarboxylase, pseudogene 1	1					1.94				
216505	Pik3ip1	phosphoinositide-3-kinase interacting protein 1	1					-1.32				
74769	Pik3cb	phosphatidylinositol 3-kinase, catalytic, beta polypeptide	1					1.48				
14755	Pigq	phosphatidylinositol glycan anchor biosynthesis, class Q	1					-1.16				
244650	Phlpp2	PH domain and leucine rich repeat protein phosphatase 2	1					-1.41				

Gene ID	Symbol	Description	DEG/Set count	2mWT_2mKO	20mWT_20mKO	2mKO_20mKO	2mWT_20mWT	2mWT_30mWT	20mWT_30mWT	Literature-Mining (Aging-Cholesterol literature)		
				9	230	406	48	1904	1157	#Paper	Enrichment	BH P-value
78246	Phf23	PHD finger protein 23	1					1.22				
230936	Phf13	PHD finger protein 13	1					-1.19				
13619	Phc1	polyhomeotic-like 1 (Drosophila)	1		1.31							
67078	Pgp	phosphoglycolate phosphatase	1					-1.27				
54381	Pgcp	plasma glutamate carboxypeptidase	1					1.35				
72542	Pgam5	phosphoglycerate mutase family member 5	1					1.35				
56612	Pfdn5	prefoldin 5	1					-1.12				
68559	Pdrg1	p53 and DNA damage regulated 1	1					-1.17				
68797	Pdgfrl	platelet-derived growth factor receptor-like	1					-1.45				
18596	Pdgfrb	platelet derived growth factor receptor, beta polypeptide	1					-1.32				
18595	Pdgfra	platelet derived growth factor receptor, alpha polypeptide	1					-1.71				
18585	Pde9a	phosphodiesterase 9A	1					-1.75				
18578	Pde4b	phosphodiesterase 4B, cAMP specific	1		-1.44							
211948	Pde12	phosphodiesterase 12	1					1.62				
18570	Pdcd6	programmed cell death 6	1					1.21				
18551	Pcsk4	proprotein convertase subtilisin/kexin type 4	1		1.31							
18546	Pcp4	Purkinje cell protein 4	1		-1.50							
93874	Pcdhb3	protocadherin beta 3	1					1.94				
23983	Pcbp1	poly(rC) binding protein 1	1					1.20				
72562	Pcbd2	pterin 4 alpha carbinolamine dehydratase/dimerization cofactor of hepatocyte nuclear factor 1 alpha (TCF1) 2	1					-1.39				
18516	Pbx3	pre B-cell leukemia transcription factor 3	1					1.75				
66923	Pbrm1	polybromo 1	1					1.22				
243771	Parp12	poly (ADP-ribose) polymerase family, member 12	1					1.31				
101187	Parp11	poly (ADP-ribose) polymerase family, member 11	1					1.58				
93737	Pard6g	par-6 partitioning defective 6 homolog gamma (C. elegans)	1					-1.27				
71904	Paqr7	progestin and adipoQ receptor family member VII	1					-1.49				
55991	Panx1	pannexin 1	1					2.01				
210622	Pamr1	peptidase domain containing associated with muscle regeneration 1	1					-1.87				
70584	Pak4	p21 protein (Cdc42/Rac)-activated kinase 4	1					-1.37				
18481	Pak3	p21 protein (Cdc42/Rac)-activated kinase 3	1		-1.74							
18451	P4ha1	procollagen-proline, 2-oxoglutarate 4-dioxygenase (proline 4-hydroxylase), alpha 1 polypeptide	1					-1.45				
14628	Ostm1	osteopetrosis associated transmembrane protein 1	1					1.28				
66844	Ormdl2	ORM1-like 2 (S. cerevisiae)	1					1.44				
227102	Ormdl1	ORM1-like 1 (S. cerevisiae)	1					1.79				
216157	ORF61	open reading frame 61	1					-1.25				
94190	Ophn1	oligophrenin 1	1					-1.39				
27047	Omd	osteomodulin	1					-2.63				
18263	Odc1	ornithine decarboxylase, structural 1	1					-1.73				
98733	Obsl1	obscurin-like 1	1					-1.38				
18242	Oat	ornithine aminotransferase	1					1.63				
18230	Nxn	nucleoredoxin	1					-1.51				
68051	Nutf2	nuclear transport factor 2	1		-1.28							
218210	Nup153	nucleoporin 153	1					1.84				
68564	Nufip2	nuclear fragile X mental retardation protein interacting protein 2	1					1.32				
18208	Ntn1	netrin 1	1					-1.32				
18203	Ntan1	N-terminal Asn amidase	1					-1.18				
103850	Nt5m	5',3'-nucleotidase, mitochondrial	1					-1.34				
18194	Nsdhl	NAD(P) dependent steroid dehydrogenase-like	1					-1.49				
106582	Nrm	nurim (nuclear envelope membrane protein)	1					2.22				
27206	Nrk	Nik related kinase	1					-4.80				
268903	Nrip1	nuclear receptor interacting protein 1	1					1.52				
18227	Nr4a2	nuclear receptor subfamily 4, group A, member 2	1					-1.22				
18159	Nppc	natriuretic peptide type C	1					-1.56				
108176	Npm3-ps1	nucleoplasmin 3, pseudogene 1	1					1.54				
74091	Npl	N-acetylneuraminate pyruvate lyase	1					1.58				
54615	Npff	neuropeptide FF-amide peptide precursor	1					-1.72				
70769	Nolc1	nucleolar and coiled-body phosphoprotein 1	1					1.23				
226518	Nmnat2	nicotinamide nucleotide adenylyltransferase 2	1		-1.75							
75533	Nme5	non-metastatic cells 5, protein expressed in (nucleoside-diphosphate kinase)	1					-1.39				
79059	Nme3	non-metastatic cells 3, protein expressed in	1					1.55				
70701	Nipal1	NIPA-like domain containing 1	1					-1.34				
93790	Nipa2	non imprinted in Prader-Willi/Angelman syndrome 2 homolog (human)	1					1.70				
105193	Nhlrc1	NHL repeat containing 1	1					-1.38				
56748	Nfu1	NFU1 iron-sulfur cluster scaffold homolog (S. cerevisiae)	1					1.24				
18037	Nfkbie	nuclear factor of kappa light polypeptide gene enhancer in B-cells inhibitor, epsilon	1					2.38				
18007	Neo1	neogenin	1					-1.32				
54003	Nell2	NEL-like 2 (chicken)	1					-1.45				

Gene ID	Symbol	Description	DEG/Set count	2mWT_2mKO	20mWT_20mKO	2mKO_20mKO	2mWT_20mWT	2mWT_30mWT	20mWT_30mWT	Literature-Mining (Aging-Cholesterol literature)		
				9	230	406	48	1904	1157	#Paper	Enrichment	BH P-value
56876	Nelf	nasal embryonic LHRH factor	1					-1.29				
104130	Ndufb11	NADH dehydrogenase (ubiquinone) 1 beta subcomplex, 11	1					1.35				
68202	Ndufa5	NADH dehydrogenase (ubiquinone) 1 alpha subcomplex, 5	1					-1.24				
67203	Nde1	nuclear distribution gene E homolog 1 (A nidulans)	1					-1.20				
320024	Nceh1	arylacetamide deacetylase-like 1	1					1.48				
68298	Ncapd2	non-SMC condensin I complex, subunit D2	1		1.42							
72560	Naalad2	N-acetylated alpha-linked acidic dipeptidase 2	1					-1.79				
72117	Naa50	N(alpha)-acetyltransferase 50, NatE catalytic subunit	1					1.31				
56292	Naa10	N(alpha)-acetyltransferase 10, NatA catalytic subunitNalpha acetyltransferase 10	1					1.38				
245049	Myrip	myosin VIIA and Rab interacting protein	1					-1.62				
17926	Myoc	myocilin	1					-1.56				
17919	Myo5b	myosin VB	1					-1.89				
17918	Myo5a	myosin VA	1					2.10				
17909	Myo10	myosin X	1					1.35				
74761	Mxra8	matrix-remodelling associated 8	1					-1.37				
67622	Mxra7	matrix-remodelling associated 7	1					-1.35				
17122	Mxd4	Max dimerization protein 4	1					-1.54				
17772	Mtm1	X-linked myotubular myopathy gene 1	1					1.97				
108156	Mthfd1	methylenetetrahydrofolate dehydrogenase (NADP+ dependent), methenyltetrahydrofolate cyclohydrolase, formyltetrahydrofolate synthase	1					-1.30				
23942	Mta2	metastasis-associated gene family, member 2	1					1.57				
17751	Mt3	metallothionein 3	1					-1.70				
545279	Ms4a15	membrane-spanning 4-domains, subfamily A, member 15	1					-1.38				
79044	Mrps34	mitochondrial ribosomal protein S34	1					1.26				
64660	Mrps24	mitochondrial ribosomal protein S24	1					-1.26				
68565	Mrps18a	mitochondrial ribosomal protein S18A	1					1.38				
67994	Mrps11	mitochondrial ribosomal protein S11	1					1.40				
94066	Mrpl36	mitochondrial ribosomal protein L36	1					1.16				
67707	Mrpl24	mitochondrial ribosomal protein L24	1					1.23				
66448	Mrpl20	mitochondrial ribosomal protein L20	1					1.52				
67681	Mrpl18	mitochondrial ribosomal protein L18	1					1.43				
246221	Mpst	mercaptopyruvate sulfurtransferase	1					1.97				
70380	Mospd1	motile sperm domain containing 1	1					1.41				
67247	Mosc2	MOCO sulphurase C-terminal domain containing 2	1					-1.32				
378462	Morn2	MORN repeat containing 2	1					-1.41				
77697	Mmab	methylmalonic aciduria (cobalamin deficiency) type B homolog (human)	1					-1.45				
210719	Mkx	mohawk homeobox	1		-2.05							
59090	Midn	midnolin	1					1.30				
76580	Mib2	mindbomb homolog 2 (Drosophila)	1					-1.45				
216864	Mgl2	macrophage galactose N-acetyl-galactosamine specific lectin 2	1					-2.23				
269181	Mgat4a	mannoside acetylglucosaminyltransferase 4, isoenzyme A	1					1.60				
217721	Mfsd7c	major facilitator superfamily domain containing 7C	1					-2.01				
69900	Mfsd11	major facilitator superfamily domain containing 11	1					1.51				
66868	Mfsd1	major facilitator superfamily domain containing 1	1					1.32				
30060	Mfi2	antigen p97 (melanoma associated) identified by monoclonal antibodies 133.2 and 96.5	1					-1.53				
76293	Mfap4	microfibrillar-associated protein 4	1					-4.69				
56335	Mettl3	methyltransferase like 3	1					-1.18				
17289	Mertk	c-mer proto-oncogene tyrosine kinase	1					1.94				
76890	Memo1	mediator of cell motility 1	1					1.48				
70417	Megf10	multiple EGF-like-domains 10	1					1.60				
20933	Med22	mediator complex subunit 22	1					-1.42				
17219	Mcm6	minichromosome maintenance deficient 6 (MIS5 homolog, S. pombe) (S. cerevisiae)	1		1.46							
230125	Mcart1	mitochondrial carrier triple repeat 1	1					1.23				
84004	Mcam	melanoma cell adhesion molecule	1					1.61				
77582	Mboat7	membrane bound O-acyltransferase domain containing 7	1					-1.17				
26390	Mapkbp1	mitogen-activated protein kinase binding protein 1	1					-1.52				
227743	Mapkap1	mitogen-activated protein kinase associated protein 1	1					-1.22				
23939	Mapk7	mitogen-activated protein kinase 7	1					-1.29				
69546	Mapk1ip1	mitogen-activated protein kinase 1 interacting protein 1	1					-1.35				
227619	Man1b1	mannosidase, alpha, class 1B, member 1	1					-1.38				
17153	Mal	myelin and lymphocyte protein, T-cell differentiation protein	1					-1.23				
75625	Mageh1	melanoma antigen, family H, 1	1					1.43				
80884	Maged2	melanoma antigen, family D, 2	1					-1.50				
18777	Lypla1	lysophospholipase 1	1					1.68				
17089	Lyar	Ly1 antibody reactive clone	1					-1.29				

Gene ID	Symbol	Description	DEG/Set count	2mWT_2mKO	20mWT_20mKO	2mKO_20mKO	2mWT_20mWT	2mWT_30mWT	20mWT_30mWT	Literature-Mining (Aging-Cholesterol literature)		
				9	230	406	48	1904	1157	#Paper	Enrichment	BH P-value
17084	Ly86	lymphocyte antigen 86	1					1.66				
114654	Ly6g6d	lymphocyte antigen 6 complex, locus G6D	1					-1.37				
17069	Ly6e	lymphocyte antigen 6 complex, locus E	1					1.31				
16997	Ltbp2	latent transforming growth factor beta binding protein 2	1		2.30							
16981	Lrrn3	leucine rich repeat protein 3, neuronal	1					-1.51				
241568	Lrrc4c	leucine rich repeat containing 4C	1		-1.33							
102747	Lrrc49	leucine rich repeat containing 49	1		-2.00							
67867	Lrrc28	leucine rich repeat containing 28	1					-1.24				
64898	Lpin2	lipin 2	1					1.51				
99633	Lphn2	latrophilin 2	1					1.35				
100503890	LOC100503890	RIKEN cDNA 2900053A13 gene	1					-1.29				
100503337	LOC100503337	predicted gene 9895	1					-1.73				
100503057	LOC100503057	uncharacterized LOC100503057	1					-1.77				
381236	Lipo1	lipase, member O1	1					1.64				
225341	Lims2	LIM and senescent cell antigen like domains 2	1					-1.31				
16859	Lgals9	lectin, galactose binding, soluble 9	1					1.49				
56401	Lepre1	leprecan 1	1					-1.44				
69757	Leng1	leukocyte receptor cluster (LRC) member 1	1					-1.61				
223732	Ldoc1l	leucine zipper, down-regulated in cancer 1-like	1					-1.44				
18826	Lcp1	lymphocyte cytosolic protein 1	1					2.62				
329504	Lcmt2	leucine carboxyl methyltransferase 2	1					1.31				
225010	Lclat1	lysocardiolipin acyltransferase 1	1					1.40				
66344	Lce3b	late cornified envelope 3B	1					-1.41				
16803	Lbp	lipopolysaccharide binding protein	1					-1.74				
16796	Lasp1	LIM and SH3 protein 1	1					-1.30				
76130	Las1l	LAS1-like (S. cerevisiae)	1					-1.24				
217980	Larp4b	La ribonucleoprotein domain family, member 4B	1					1.21				
236285	Lancl3	LanC lantibiotic synthetase component C-like 3 (bacterial)	1		-2.07							
16777	Lamb1	laminin B1	1		1.26							
69815	Krtcap3	keratinocyte associated protein 3	1					-1.29				
68239	Krt42	keratin 42	1					-1.51				
16670	Krt32	keratin 32	1					-1.48				
66809	Krt20	keratin 20	1					-1.91				
16648	Kpna3	karyopherin (importin) alpha 3	1					1.38				
16646	Kpna1	karyopherin (importin) alpha 1	1					1.31				
16631	Klra13-ps	killer cell lectin-like receptor subfamily A, member 13, pseudogene	1					-1.67				
16604	Klk1b7-ps	kallikrein 1-related peptidase b7, pseudogene	1					-1.51				
234378	Klhl26	kelch-like 26 (Drosophila)	1					-1.23				
277396	Klhl23	kelch-like 23 (Drosophila)	1					-1.62				
231003	Klhl17	kelch-like 17 (Drosophila)	1					-1.31				
232943	Klc3	kinesin light chain 3	1					1.46				
16576	Kif7	kinesin family member 7	1					-1.33				
75605	Kdm5b	lysine (K)-specific demethylase 5B	1					-1.33				
67516	Kctd4	potassium channel tetramerisation domain containing 4	1					-1.38				
16511	Kcnh2	potassium voltage-gated channel, subfamily H (eag-related), member 2	1		-1.39							
16502	Kcnc1	potassium voltage gated channel, Shaw-related subfamily, member 1	1		-2.14							
242553	Kank4	KN motif and ankyrin repeat domains 4	1					-1.17				
235041	Kank2	KN motif and ankyrin repeat domains 2	1					-1.24				
338523	Jhdm1d	jumonji C domain-containing histone demethylase 1 homolog D (S. cerevisiae)	1					1.51				
16451	Jak1	Janus kinase 1	1					1.40				
67767	Jagn1	jagunal homolog 1 (Drosophila)	1					1.23				
16449	Jag1	jagged 1	1					-1.39				
16431	Itm2a	integral membrane protein 2A	1					-1.61				
16400	Itga3	integrin alpha 3	1					-1.34				
75847	Ispd	isoprenoid synthase domain containing	1					1.71				
15944	Irgm1	immunity-related GTPase family M member 1	1					1.87				
108960	Irak2	interleukin-1 receptor-associated kinase 2	1					2.31				
232227	Iqsec1	IQ motif and Sec7 domain 1	1					-1.19				
16190	Il4ra	interleukin 4 receptor, alpha	1					1.50				
16186	Il2rg	interleukin 2 receptor, gamma chain	1					5.29				
16172	Il17ra	interleukin 17 receptor A	1					2.12				
16170	Il16	interleukin 16	1					-1.36				
67454	Ikbip	IKBKB interacting protein	1					-1.32				
16069	Igj	immunoglobulin joining chain	1					28.96				
18518	Igbp1	immunoglobulin (CD79A) binding protein 1	1					1.46				
15939	Ier5	immediate early response 5	1					1.40				
15937	Ier3	immediate early response 3	1					1.82				
15903	Id3	inhibitor of DNA binding 3	1					1.43				
105148	Iars	isoleucine-tRNA synthetase	1					1.37				
67732	Iah1	isoamyl acetate-hydrolyzing esterase 1 homolog (S. cerevisiae)	1					1.23				
64704	Htra2	HtrA serine peptidase 2	1					-1.23				
15512	Hspa2	heat shock protein 2	1					1.36				
72630	Hspa12b	heat shock protein 12B	1					-1.71				
15473	Hrsp12	heat-responsive protein 12	1					1.53				
15446	Hpgd	hydroxyprostaglandin dehydrogenase 15 (NAD)	1					-1.45				
15438	Hoxd9	homeobox D9	1		-1.33							

Gene ID	Symbol	Description	DEG/Set count	2mWT_2mKO	20mWT_20mKO	2mKO_20mKO	2mWT_20mWT	2mWT_30mWT	20mWT_30mWT	Literature-Mining (Aging-Cholesterol literature)		
				9	230	406	48	1904	1157	#Paper	Enrichment	BH P-value
15402	Hoxa5	homeobox A5	1					-1.44				
26557	Homer2	homer homolog 2 (Drosophila)	1					-1.60				
15384	Hnrnpab	heterogeneous nuclear ribonucleoprotein A/B	1					1.24				
15273	Hivep2	human immunodeficiency virus type I enhancer binding protein 2	1					1.25				
319190	Hist2h2be	histone cluster 2, H2be	1					-1.38				
71059	Hexim2	hexamethylene bis-acetamide inducible 2	1					-1.29				
223658	Heatr7a	HEAT repeat containing 7A	1					1.46				
29877	Hdgfrp3	hepatoma-derived growth factor, related protein 3	1					-1.24				
208727	Hdac4	histone deacetylase 4	1					1.53				
330790	Hapln4	hyaluronan and proteoglycan link protein 4	1		-1.75							
56794	Hacl1	2-hydroxyacyl-CoA lyase 1	1					-1.61				
77605	H2afv	H2A histone family, member V	1					-1.36				
14955	H19	H19 fetal liver mRNA	1					-1.67				
232493	Gys2	glycogen synthase 2	1		3.64							
107999	Gtpbp6	GTP binding protein 6 (putative)	1					-1.36				
14894	Gtl3	gene trap locus 3	1					-1.26				
98053	Gtf2f1	general transcription factor IIF, polypeptide 1	1					1.22				
68153	Gtf2e2	general transcription factor II E, polypeptide 2 (beta subunit)	1					1.72				
235459	Gtf2a2	general transcription factor II A, 2	1					-1.13				
68214	Gsto2	glutathione S-transferase omega 2	1					-1.60				
68312	Gstm7	glutathione S-transferase, mu 7	1					-1.43				
227753	Gsn	gelsolin	1					-1.15				
382034	Gse1	genetic suppressor element 1	1					1.61				
231413	Grsf1	G-rich RNA sequence binding factor 1	1					-1.16				
28015	Grinl1a	polymerase (RNA) II (DNA directed) polypeptide M	1					1.29				
170483	Grin3b	glutamate receptor, ionotropic, NMDA3B	1					-1.29				
14809	Grik5	glutamate receptor, ionotropic, kainate 5 (gamma 2)	1					-1.67				
14790	Grcc10	gene rich cluster, C10 gene	1					1.21				
67839	Gpsm1	G-protein signalling modulator 1 (AGS3-like, C. elegans)	1					-1.58				
64095	Gpr35	G protein-coupled receptor 35	1					2.16				
239530	Gpr20	G protein-coupled receptor 20	1					-1.57				
58245	Gpr180	G protein-coupled receptor 180	1					-1.20				
269053	Gpr152	G protein-coupled receptor 152	1					-1.54				
100210	Gpn2	GPN-loop GTPase 2	1					1.50				
14733	Gpc1	glypican 1	1					1.21				
70231	Gorasp2	golgi reassembly stacking protein 2	1					1.42				
71146	Golga7b	golgi autoantigen, golgin subfamily a, 7B	1		-1.76							
30877	Gnl3	guanine nucleotide binding protein-like 3 (nucleolar)	1		1.18							
14694	Gnb2l1	guanine nucleotide binding protein (G protein), beta polypeptide 2 like 1	1					1.15				
14674	Gna13	guanine nucleotide binding protein, alpha 13	1					1.32				
668339	Gm9112	predicted gene 9112	1					-1.65				
546143	Gm5918	predicted gene 5918	1					-1.36				
242037	Gm410	predicted gene 410	1		2.18							
241289	Gm347	protein phosphatase 1, regulatory subunit 26	1					-1.53				
100040297	Gm2695	predicted gene 2695	1					1.34				
100504674	Gm20346	predicted gene, 20346	1					-1.26				
100504048	Gm20033	predicted gene, 20033	1					-1.81				
100503686	Gm19831	predicted gene, 19831	1					1.72				
100504345	Gm17456	predicted gene, 17456	1		-2.84							
100503986	Gm17227	predicted gene 17227	1					-1.53				
24083	Gm16515	predicted gene, Gm16515	1		1.36							
100503471	Gm15867	predicted gene 15867	1					-1.72				
100042125	Gm15535	predicted gene 15535	1					-2.38				
100044509	Gm14378	predicted gene 14378	1					-1.58				
100036520	Gm14318	predicted gene 14318	1					-1.72				
100503381	Gm14022	predicted gene 14022	1					-1.96				
545681	Gm12992	predicted gene 12992	1					-1.48				
629750	Gm11517	ubiquitin A-52 residue ribosomal protein fusion product 1 pseudogene	1					-1.64				
226866	Gm106	predicted gene 106	1					-1.31				
100038398	Gm10567	predicted gene 10567	1					1.33				
328186	Gm10336	predicted gene 10336	1					-1.31				
68077	Gltscr2	glioma tumor suppressor candidate region gene 2	1					1.35				
109801	Glo1	glyoxalase 1	1					-1.15				
170823	Glmn	glomulin, FKBP associated protein	1					-1.24				
14622	Gjb5	gap junction protein, beta 5	1					-1.64				
83408	Gimap3	GTPase, IMAP family member 3	1					7.29				
16205	Gimap1	GTPase, IMAP family member 1	1		1.45							
74105	Gga2	golgi associated, gamma adaptin ear containing, ARF binding protein 2	1					1.31				
233552	Gdpd5	glycerophosphodiester phosphodiesterase domain containing 5	1					-1.47				
229900	Gbp7	guanylate binding protein 7	1					1.41				
55932	Gbp3	guanylate binding protein 3	1					1.57				
74185	Gbe1	glucan (1,4-alpha-), branching enzyme 1	1					2.31				

Gene ID	Symbol	Description	DEG/Set count	2mWT_2mKO	20mWT_20mKO	2mKO_20mKO	2mWT_20mWT	2mWT_30mWT	20mWT_30mWT	Literature-Mining (Aging-Cholesterol literature)		
				9	230	406	48	1904	1157	#Paper	Enrichment	BH P-value
384281	Gatc	glutamyl-tRNA(Gln) amidotransferase, subunit C homolog (bacterial)	1					-1.20				
14456	Gas6	growth arrest specific 6	1					-1.39				
14451	Gas1	growth arrest specific 1	1					-1.73				
207839	Galnt6	UDP-N-acetyl-alpha-D-galactosamine:polypeptide N-acetylgalactosaminyltransferase 6	1					2.63				
171212	Galnt10	UDP-N-acetyl-alpha-D-galactosamine:polypeptide N-acetylgalactosaminyltransferase 10	1					-1.52				
14420	Galc	galactosylceramidase	1					1.59				
231580	Gak	cyclin G associated kinase	1					1.28				
14405	Gabrg1	gamma-aminobutyric acid (GABA) A receptor, subunit gamma 1	1		-2.75							
14086	Fscn1	fascin homolog 1, actin bundling protein (Strongylocentrotus purpuratus)	1					-1.44				
68655	Fndc1	fibronectin type III domain containing 1	1					-1.53				
14263	Fmo5	flavin containing monooxygenase 5	1					-1.42				
71409	Fmnl2	formin-like 2	1					-1.16				
14255	Flt3	FMS-like tyrosine kinase 3	1		-1.67							
286940	Flnb	filamin, beta	1					-1.31				
14231	Fkbp7	FK506 binding protein 7	1					-1.25				
14229	Fkbp5	FK506 binding protein 5	1					1.61				
231997	Fkbp14	FK506 binding protein 14	1					-1.23				
60344	Fign	fidgetin	1					1.42				
58249	Fibp	fibroblast growth factor (acidic) intracellular binding protein	1					-1.28				
67529	Fgfr1op2	FGFR1 oncogene partner 2	1					1.48				
14172	Fgf18	fibroblast growth factor 18	1		-2.22							
59083	Fetub	fetuin beta	1		2.90							
207278	Fchsd2	FCH and double SH3 domains 2	1					1.74				
215384	Fcgbp	Fc fragment of IgG binding protein	1					-2.06				
14127	Fcer1g	Fc receptor, IgE, high affinity I, gamma polypeptide	1		1.38							
50753	Fbxo8	F-box protein 8	1					1.85				
69754	Fbxo7	F-box protein 7	1					1.23				
66822	Fbxo25	F-box protein 25	1					-1.40				
101358	Fbxl14	F-box and leucine-rich repeat protein 14	1					1.28				
14114	Fbln1	fibulin 1	1					-1.64				
14089	Fap	fibroblast activation protein	1					-1.70				
237211	Fancb	Fanconi anemia, complementation group B	1					-1.53				
385658	Fam55c	family with sequence similarity 55, member C	1		-1.56							
66294	Fam3a	family with sequence similarity 3, member A	1					-1.26				
381217	Fam189a2	family with sequence similarity 189, member A2	1					-1.43				
70638	Fam189a1	family with sequence similarity 189, member A1	1					-1.64				
381337	Fam178b	family with sequence similarity 178, member B	1					-1.27				
226151	Fam178a	family with sequence similarity 178, member A	1					1.49				
68675	Fam172a	family with sequence similarity 172, member A	1					1.57				
74349	Fam160a2	family with sequence similarity 160, member A2	1					-1.30				
230579	Fam151a	family with sequence similarity 151, member A	1					-1.37				
227298	Fam134a	family with sequence similarity 134, member A	1					-1.18				
218236	Fam120a	family with sequence similarity 120, member A	1					1.22				
72750	Fam117b	family with sequence similarity 117, member B	1					1.36				
68303	Fam114a1	family with sequence similarity 114, member A1	1					-1.31				
268709	Fam107a	family with sequence similarity 107, member A	1					-1.50				
14038	Expi	extracellular proteinase inhibitor	1		3.12							
211446	Exoc3	exocyst complex component 3	1					-1.24				
14011	Etv6	ets variant gene 6 (TEL oncogene)	1					1.45				
23943	Esyt1	extended synaptotagmin-like protein 1	1					1.35				
207920	Esrp1	epithelial splicing regulatory protein 1	1					-2.52				
67397	Erp29	endoplasmic reticulum protein 29	1					1.40				
66366	Ergic3	ERGIC and golgi 3	1					-1.19				
22592	Ercc5	excision repair cross-complementing rodent repair deficiency, complementation group 5	1					-1.21				
111173	Erc1	ELKS/RAB6-interacting/CAST family member 1	1					-1.28				
59079	Erbb2ip	Erbb2 interacting protein	1					1.48				
13516	Epyc	epiphycan	1		1.84							
13848	Ephb6	Eph receptor B6	1					-1.46				
270190	Ephb1	Eph receptor B1	1		1.50							
18606	Enpp2	ectonucleotide pyrophosphatase/phosphodiesterase 2	1					-1.33				
67870	Enoph1	enolase-phosphatase 1	1					-1.22				
54325	Elovl1	elongation of very long chain fatty acids (FEN1/Elo2, SUR4/Elo3, yeast)-like 1	1					-1.17				
192170	Eif4a3	eukaryotic translation initiation factor 4A3	1		1.21							
54709	Eif3i	eukaryotic translation initiation factor 3, subunit I	1					1.17				
55944	Eif3d	eukaryotic translation initiation factor 3, subunit D	1					1.42				
66235	Eif1ax	eukaryotic translation initiation factor 1A, X-linked	1					1.32				
98878	Ehd4	EH-domain containing 4	1					1.19				
259300	Ehd2	EH-domain containing 2	1					-1.34				
112405	Egln1	EGL nine homolog 1 (C. elegans)	1					-1.30				
353156	Egfl7	EGF-like domain 7	1					-1.50				
71877	Efhc1	EF-hand domain (C-terminal) containing 1	1					-1.44				

Gene ID	Symbol	Description	DEG/Set count	2mWT_2mKO	20mWT_20mKO	2mKO_20mKO	2mWT_20mWT	2mWT_30mWT	20mWT_30mWT	Literature-Mining (Aging-Cholesterol literature)		
				9	230	406	48	1904	1157	#Paper	Enrichment	BH P-value
216238	Eea1	early endosome antigen 1	1					1.43				
107522	Ece2	endothelin converting enzyme 2	1					1.47				
55960	Ebag9	estrogen receptor-binding fragment-associated gene 9	1					1.67				
223593	E430025E21Rik	RIKEN cDNA E430025E21 gene	1					1.15				
66573	Dzip1	DAZ interacting protein 1	1					-1.32				
80915	Dusp12	dual specificity phosphatase 12	1					-1.44				
209200	Dtx3l	deltex 3-like (Drosophila)	1					1.60				
434423	Dppa5a	developmental pluripotency associated 5A	1					-1.39				
13482	Dpp4	dipeptidylpeptidase 4	1					-1.85				
238130	Dock4	dedicator of cytokinesis 4	1					2.40				
13421	Dnase1l3	deoxyribonuclease 1-like 3	1		-1.71							
13002	Dnajc5	DnaJ (Hsp40) homolog, subfamily C, member 5	1					1.27				
69408	Dnajc17	DnaJ (Hsp40) homolog, subfamily C, member 17	1					-1.46				
30045	Dnajc12	DnaJ (Hsp40) homolog, subfamily C, member 12	1					2.28				
106794	Dhx57	DEAH (Asp-Glu-Ala-Asp/His) box polypeptide 57	1					1.58				
20148	Dhrs3	dehydrogenase/reductase (SDR family) member 3	1					1.39				
52585	Dhrs1	dehydrogenase/reductase (SDR family) member 1	1					1.42				
116891	Derl2	Der1-like domain family, member 2	1					1.30				
232449	Dera	2-deoxyribose-5-phosphate aldolase homolog (C. elegans)	1		1.44							
211896	Depdc7	DEP domain containing 7	1					-1.43				
54006	Deaf1	deformed epidermal autoregulatory factor 1 (Drosophila)	1					-1.46				
53817	Ddx39b	DEAD (Asp-Glu-Ala-Asp) box polypeptide 39B	1					1.14				
56200	Ddx21	DEAD (Asp-Glu-Ala-Asp) box polypeptide 21	1					1.50				
67040	Ddx17	DEAD (Asp-Glu-Ala-Asp) box polypeptide 17	1					-1.19				
114874	Ddhd1	DDHD domain containing 1	1					1.43				
69305	Dcps	decapping enzyme, scavenger	1					1.45				
73379	Dcbld2	discoidin, CUB and LCCL domain containing 2	1					-1.24				
226414	Dars	aspartyl-tRNA synthetase	1					1.87				
57373	D930014E17Rik	RIKEN cDNA D930014E17 gene	1					-1.46				
654810	D630032N06Rik	RIKEN cDNA D630032N06 gene	1					-1.40				
52392	D1Ertd622e	DNA segment, Chr 1, ERATO Doi 622, expressed	1					1.64				
52318	D1Ertd448e	DNA segment, Chr 1, ERATO Doi 448, expressed	1					1.40				
27762	D17H6S56E-3	DNA segment, Chr 17, human D6S56E 3	1		-2.33							
100504016	D130020L05Rik	NA	1					-1.54				
27528	D0H4S114	DNA segment, human D4S114	1					-1.55				
225995	D030056L22Rik	RIKEN cDNA D030056L22 gene	1					-1.28				
13113	Cyp3a13	cytochrome P450, family 3, subfamily a, polypeptide 13	1		1.69							
13107	Cyp2f2	cytochrome P450, family 2, subfamily f, polypeptide 2	1		-6.13							
56448	Cyp2d22	cytochrome P450, family 2, subfamily d, polypeptide 22	1					-1.30				
66427	Cyb5b	cytochrome b5 type B	1					-1.29				
319478	Cxxc4	CXXC finger 4	1		-2.14							
12778	Cxcr7	chemokine (C-X-C motif) receptor 7	1					-1.47				
80744	Cwc22	CWC22 spliceosome-associated protein homolog (S. cerevisiae)	1					1.82				
66070	Cwc15	CWC15 homolog (S. cerevisiae)	1					1.21				
13043	Cttn	cortactin	1					1.20				
13039	Ctsl	cathepsin L	1					1.41				
13032	Ctsc	cathepsin C	1					1.34				
12385	Ctnna1	catenin (cadherin associated protein), alpha 1	1					-1.13				
269037	Ctif	CBP80/20-dependent translation initiation factor	1		-1.42							
227292	Ctdsp1	CTD (carboxy-terminal domain, RNA polymerase II, polypeptide A) small phosphatase 1	1					-1.10				
217615	Ctage5	CTAGE family, member 5	1		1.24							
83691	Crispld1	cysteine-rich secretory protein LCCL domain containing 1	1					-1.33				
208677	Creb3l3	cAMP responsive element binding protein 3-like 3	1					-1.61				
12873	Cpa3	carboxypeptidase A3, mast cell	1		2.24							
20463	Cox7a2l	cytochrome c oxidase subunit VIIa polypeptide 2-like	1					1.12				
12864	Cox6c	cytochrome c oxidase, subunit VIc	1					-1.17				
12721	Coro1a	coronin, actin binding protein 1A	1					2.02				
53419	Corin	corin	1					1.98				
56358	Copz2	coatomer protein complex, subunit zeta 2	1					-1.57				
66200	Commd6	COMM domain containing 6	1					1.32				
66398	Commd5	COMM domain containing 5	1					1.22				
66199	Commd4	COMM domain containing 4	1					1.38				
69456	Commd10	COMM domain containing 10	1					-1.22				
17846	Commd1	COMM domain containing 1	1					1.49				
12841	Col9a3	collagen, type IX, alpha 3	1		1.63							
12833	Col6a1	collagen, type VI, alpha 1	1					-1.51				
53867	Col5a3	collagen, type V, alpha 3	1					-1.66				

Gene ID	Symbol	Description	DEG/Set count	2mWT_2mKO	20mWT_20mKO	2mKO_20mKO	2mWT_20mWT	2mWT_30mWT	20mWT_30mWT	Literature-Mining (Aging-Cholesterol literature)		
				9	230	406	48	1904	1157	#Paper	Enrichment	BH P-value
12826	Col4a1	collagen, type IV, alpha 1	1					-1.33				
12819	Col15a1	collagen, type XV, alpha 1	1					-1.28				
12810	Coch	coagulation factor C homolog (Limulus polyphemus)	1					1.86				
12801	Cnr1	cannabinoid receptor 1 (brain)	1		-1.71							
104625	Cnot6	CCR4-NOT transcription complex, subunit 6	1					1.60				
94218	Cnnm3	cyclin M3	1					-1.33				
83674	Cnnm1	cyclin M1	1		-2.11							
71994	Cnn3	calponin 3, acidic	1					-1.20				
98417	Cnih4	cornichon homolog 4 (Drosophila)	1					-1.35				
67213	Cmtm6	CKLF-like MARVEL transmembrane domain containing 6	1					-1.48				
69574	Cmbl	carboxymethylenebutenolidase-like (Pseudomonas)	1					-1.19				
17228	Cma1	chymase 1, mast cell	1		2.27							
74325	Cltb	clathrin, light polypeptide (Lcb)	1					-1.31				
12757	Clta	clathrin, light polypeptide (Lca)	1					1.20				
76524	Cln6	ceroid-lipofuscinosis, neuronal 6	1					1.21				
78785	Clip4	CAP-GLY domain containing linker protein family, member 4	1		-1.33							
29876	Clic4	chloride intracellular channel 4 (mitochondrial)	1					1.31				
69454	Clic3	chloride intracellular channel 3	1					-1.43				
114584	Clic1	chloride intracellular channel 1	1					1.57				
69810	Clec4b1	C-type lectin domain family 4, member b1	1					-1.91				
665180	Clec2l	C-type lectin domain family, member L	1		-1.95							
56173	Cldn14	claudin 14	1					-1.86				
18417	Cldn11	claudin 11	1					2.12				
58187	Cldn10	claudin 10	1					-2.19				
12728	Clcn5	chloride channel 5	1					-1.64				
12716	Ckmt1	creatine kinase, mitochondrial 1, ubiquitous	1		-2.28							
67006	Cisd2	CDGSH iron sulfur domain 2	1					1.20				
100910	Chpf2	chondroitin polymerizing factor 2	1					1.80				
246048	Chodl	chondrolectin	1		-2.65							
12662	Chm	choroidermia	1					1.28				
218865	Chdh	choline dehydrogenase	1					-1.41				
269610	Chd5	chromodomain helicase DNA binding protein 5	1					1.83				
214685	Chadl	chondroadherin-like	1					-1.55				
12633	Cflar	CASP8 and FADD-like apoptosis regulator	1					1.35				
234673	Ces2e	carboxylesterase 2E	1					-1.57				
223753	Cerk	ceramide kinase	1					1.49				
99151	Cercam	cerebral endothelial cell adhesion molecule	1					-1.58				
12609	Cebpd	CCAAT/enhancer binding protein (C/EBP), delta	1					1.38				
52626	Cdkn2aipnl	CDKN2A interacting protein N-terminal like	1					1.67				
237459	Cdk17	cyclin-dependent kinase 17	1					1.83				
12560	Cdh3	cadherin 3	1					-1.50				
214498	Cdc73	cell division cycle 73, Paf1/RNA polymerase II complex component, homolog (S. cerevisiae)	1					1.51				
56699	Cdc42ep4	CDC42 effector protein (Rho GTPase binding) 4	1					-1.24				
12517	Cd72	CD72 antigen	1					2.70				
12500	Cd3d	CD3 antigen, delta polypeptide	1					6.90				
235505	Cd109	CD109 antigen	1					1.39				
12469	Cct8	chaperonin containing Tcp1, subunit 8 (theta)	1					1.21				
12462	Cct3	chaperonin containing Tcp1, subunit 3 (gamma)	1					1.24				
227210	Ccnyl1	cyclin Y-like 1	1					-1.18				
17151	Ccndbp1	cyclin D-type binding-protein 1	1					1.89				
12425	Cckar	cholecystokinin A receptor	1		-2.95							
67200	Ccdc77	coiled-coil domain containing 77	1					-1.40				
208908	Ccdc62	coiled-coil domain containing 62	1					-1.63				
232933	Ccdc61	coiled-coil domain containing 61	1					-1.50				
52469	Ccdc56	coiled-coil domain containing 56	1					1.32				
76380	Ccdc46	coiled-coil domain containing 46	1					-1.48				
52715	Ccdc43	coiled-coil domain containing 43	1					1.39				
108811	Ccdc122	coiled-coil domain containing 122	1					1.39				
66815	Ccdc109b	coiled-coil domain containing 109B	1					1.30				
232821	Ccdc106	coiled-coil domain containing 106	1					-1.26				
12416	Cbx2	chromobox homolog 2 (Drosophila Pc class)	1					-1.38				
12390	Cav2	caveolin 2	1					-1.37				
12364	Casp12	caspase 12	1					1.61				
107239	Carns1	carnosine synthase 1	1					-1.63				
105844	Card10	caspase recruitment domain family, member 10	1					-1.54				
12351	Car4	carbonic anhydrase 4	1					-1.85				
71934	Car13	carbonic anhydrase 13	1		1.51							
12343	Capza2	capping protein (actin filament) muscle Z-line, alpha 2	1					1.26				
12338	Capn6	calpain 6	1					-1.68				
12332	Capg	capping protein (actin filament), gelsolin-like	1					1.15				
12331	Cap1	CAP, adenylate cyclase-associated protein 1 (yeast)	1					1.55				
12796	Camp	cathelicidin antimicrobial peptide	1					-2.03				
67488	Calcoco1	calcium binding and coiled coil domain 1	1					-1.29				
12308	Calb2	calbindin 2	1					-2.32				
320405	Cadps2	Ca2+-dependent activator protein for secretion 2	1		-1.80							
12289	Cacna1d	calcium channel, voltage-dependent, L type, alpha 1D subunit	1					-1.43				

Gene ID	Symbol	Description	DEG/Set count	2mWT_2mKO	20mWT_20mKO	2mKO_20mKO	2mWT_20mWT	2mWT_30mWT	20mWT_30mWT	Literature-Mining (Aging-Cholesterol literature)		
				9	230	406	48	1904	1157	#Paper	Enrichment	BH P-value
320508	Cachd1	cache domain containing 1	1		1.54							
12283	Cab39	calcium binding protein 39	1					1.25				
320295	C920006O11Rik	RIKEN cDNA C920006O11 gene	1					-1.43				
97402	C86187	expressed sequence C86187	1					-1.56				
12274	C6	complement component 6	1					2.19				
77644	C330007P06Rik	RIKEN cDNA C330007P06 gene	1					1.39				
101744	C330005M16Rik	RIKEN cDNA C330005M16 gene	1					-1.43				
101831	C230052I12Rik	RIKEN cDNA C230052I12 gene	1					1.50				
320842	C230035I16Rik	RIKEN cDNA C230035I16 gene	1					-1.34				
12263	C2	complement component 2 (within H-2S)	1					-1.56				
69183	C1qtnf2	C1q and tumor necrosis factor related protein 2	1					-1.74				
12261	C1qbp	complement component 1, q subcomponent binding protein	1					1.20				
109284	C030046I01Rik	RIKEN cDNA C030046I01 gene	1					1.69				
12215	Bsg	basigin	1					1.31				
114642	Brdt	bromodomain, testis-specific	1					-1.35				
109880	Braf	Braf transforming gene	1					1.24				
228802	Bpifb5	BPI fold containing family B, member 5	1					-1.38				
66162	Bola2	bolA-like 2 (E. coli)	1					1.21				
51800	Bok	BCL2-related ovarian killer protein	1					-1.51				
224630	Bnip1	BCL2/adenovirus E1B interacting protein 1	1					1.59				
12162	Bmp7	bone morphogenetic protein 7	1					-1.58				
12159	Bmp4	bone morphogenetic protein 4	1					-1.83				
12151	Bmi1	Bmi1 polycomb ring finger oncogene	1					1.23				
233016	Blvrb	biliverdin reductase B (flavin reductase (NADPH))	1					1.41				
30948	Bin1	bridging integrator 1	1					1.99				
24115	Best1	bestrophin 1	1					-1.37				
66165	Bccip	BRCA2 and CDKN1A interacting protein	1					1.37				
12035	Bcat1	branched chain aminotransferase 1, cytosolic	1		-2.10							
216292	BC067068	cDNA sequence BC067068	1					-1.39				
414077	BC056474	cDNA sequence BC056474	1					1.12				
408058	BC048507	cDNA sequence BC048507	1					-1.67				
270802	BC048403	cDNA sequence BC048403	1					1.29				
192976	BC046404	cDNA sequence BC046404	1					-1.32				
226527	BC026585	cDNA sequence BC026585	1					-1.38				
216152	BC005764	cDNA sequence BC005764	1					1.37				
23825	Banf1	barrier to autointegration factor 1	1					1.37				
228536	Bahd1	bromo adjacent homology domain containing 1	1					1.40				
56336	B4galt5	UDP-Gal:betaGlcNAc beta 1,4-galactosyltransferase, polypeptide 5	1					1.61				
26877	B3galt1	UDP-Gal:betaGlcNAc beta 1,3-galactosyltransferase, polypeptide 1	1		-1.94							
319527	B230325K18Rik	RIKEN cDNA B230325K18 gene	1					-1.43				
54375	Azin1	antizyme inhibitor 1	1					1.53				
26362	Axl	AXL receptor tyrosine kinase	1					1.53				
11966	Atp6v1b2	ATPase, H+ transporting, lysosomal V1 subunit B2	1					1.56				
11964	Atp6v1a	ATPase, H+ transporting, lysosomal V1 subunit A	1					1.61				
242341	Atp6v0d2	ATPase, H+ transporting, lysosomal V0 subunit D2	1		3.66							
114143	Atp6v0b	ATPase, H+ transporting, lysosomal V0 subunit B	1					1.22				
67972	Atp2b1	ATPase, Ca++ transporting, plasma membrane 1	1					1.36				
11793	Atg5	autophagy-related 5 (yeast)	1					1.31				
54343	Atf7ip	activating transcription factor 7 interacting protein	1		1.43							
66695	Aspn	asporin	1					-2.52				
11484	Aspa	aspartoacylase	1					1.38				
56495	Asna1	arsA arsenite transporter, ATP-binding, homolog 1 (bacterial)	1					1.48				
11890	Asgr2	asialoglycoprotein receptor 2	1					-1.82				
230837	Asap3	ArfGAP with SH3 domain, ankyrin repeat and PH domain 3	1					-1.55				
76219	Arxes1	adipocyte-related X-chromosome expressed sequence 1	1					-1.97				
11877	Arvcf	armadillo repeat gene deleted in velo-cardio-facial syndrome	1					-1.33				
11881	Arsb	arylsulfatase B	1					1.22				
56378	Arpc3	actin related protein 2/3 complex, subunit 3	1					1.29				
56443	Arpc1a	actin related protein 2/3 complex, subunit 1A	1					1.38				
494468	Armcx5	armadillo repeat containing, X-linked 5	1					1.54				
80981	Arl4d	ADP-ribosylation factor-like 4D	1					-1.80				
68146	Arl13b	ADP-ribosylation factor-like 13B	1					1.52				
23806	Arih1	ariadne ubiquitin-conjugating enzyme E2 binding protein homolog 1 (Drosophila)	1					1.26				
58996	Arhgap23	Rho GTPase activating protein 23	1					-1.23				
73910	Arhgap18	Rho GTPase activating protein 18	1					-1.36				
99371	Arfgef2	ADP-ribosylation factor guanine nucleotide-exchange factor 2 (brefeldin A-inhibited)	1					1.28				
11843	Arf4	ADP-ribosylation factor 4	1					1.51				
11840	Arf1	ADP-ribosylation factor 1	1					1.89				

Gene ID	Symbol	Description	DEG/Set count	2mWT_2mKO	20mWT_20mKO	2mKO_20mKO	2mWT_20mWT	2mWT_30mWT	20mWT_30mWT	Literature-Mining (Aging-Cholesterol literature)		
				9	230	406	48	1904	1157	#Paper	Enrichment	BH P-value
212285	Arap2	ArfGAP with RhoGAP domain, ankyrin repeat and PH domain 2	1					1.53				
11821	Aprt	adenine phosphoribosyl transferase	1					1.27				
11425	Apoc4	apolipoprotein C-IV	1					3.76				
23796	Aplnr	apelin receptor	1					-2.66				
71770	Ap2b1	adaptor-related protein complex 2, beta 1 subunit	1					1.26				
11769	Ap1s1	adaptor protein complex AP-1, sigma 1	1					1.33				
71872	Aox4	aldehyde oxidase 4	1		-3.20							
11752	Anxa8	annexin A8	1					-2.00				
11747	Anxa5	annexin A5	1					1.36				
225187	Ankrd29	ankyrin repeat domain 29	1		-1.92							
232339	Ankrd26	ankyrin repeat domain 26	1					-1.38				
72713	Angptl1	angiopoietin-like 1	1					-2.10				
66156	Anapc11	anaphase promoting complex subunit 11	1					-1.23				
11717	Ampd3	adenosine monophosphate deaminase 3	1					1.29				
72041	Alkbh4	alkB, alkylation repair homolog 4 (E. coli)	1					1.31				
56752	Aldh9a1	aldehyde dehydrogenase 9, subfamily A1	1					-1.34				
621603	Aldh3b2	aldehyde dehydrogenase 3 family, member B2	1		4.41							
11997	Akr1b7	aldo-keto reductase family 1, member B7	1					-1.85				
11641	Akap2	A kinase (PRKA) anchor protein 2	1					1.32				
100041194	Ahnak2	AHNAK nucleoprotein 2	1					1.44				
66395	Ahnak	AHNAK nucleoprotein (desmoyokin)	1		1.22							
52906	Ahi1	Abelson helper integration site 1	1					-1.26				
230793	Ahdc1	AT hook, DNA binding motif, containing 1	1					-1.48				
28169	Agpat3	1-acylglycerol-3-phosphate O-acyltransferase 3	1					-1.23				
216549	Aftph	aftiphilin	1					1.19				
11529	Adh7	alcohol dehydrogenase 7 (class IV), mu or sigma polypeptide	1					-1.54				
69117	Adh6a	alcohol dehydrogenase 6A (class V)	1					-2.13				
11532	Adh5	alcohol dehydrogenase 5 (class III), chi polypeptide	1		1.10							
11514	Adcy8	adenylate cyclase 8	1					-1.53				
268822	Adck5	aarF domain containing kinase 5	1					-1.38				
231821	Adap1	ArfGAP with dual PH domains 1	1					-1.61				
216725	Adamts2	a disintegrin-like and metallopeptidase (reprolysin type) with thrombospondin type 1 motif, 2	1					-1.77				
11499	Adam5	a disintegrin and metallopeptidase domain 5	1					-1.55				
11496	Adam22	a disintegrin and metallopeptidase domain 22	1		-1.62							
11488	Adam11	a disintegrin and metallopeptidase domain 11	1					2.19				
66204	Acyp1	acylphosphatase 1, erythrocyte (common) type	1					1.47				
71670	Acy3	aspartoacylase (aminoacylase) 3	1					-1.74				
74117	Actr3	ARP3 actin-related protein 3 homolog (yeast)	1					1.38				
11468	Actg2	actin, gamma 2, smooth muscle, enteric	1					2.92				
11595	Acan	aggrecan	1					-1.59				
113868	Acaa1a	acetyl-Coenzyme A acyltransferase 1A	1					-1.32				
320712	Abi3bp	ABI gene family, member 3 (NESH) binding protein	1					-1.49				
19299	Abcd3	ATP-binding cassette, sub-family D (ALD), member 3	1					-1.12				
239273	Abcc4	ATP-binding cassette, sub-family C (CFTR/MRP), member 4	1					1.39				
18669	Abcb1b	ATP-binding cassette, sub-family B (MDR/TAP), member 1B	1					1.87				
27404	Abca8b	ATP-binding cassette, sub-family A (ABC1), member 8b	1		1.77							
76184	Abca6	ATP-binding cassette, sub-family A (ABC1), member 6	1		1.90							
30956	Aass	aminoadipate-semialdehyde synthase	1					-1.90				
239435	Aard	alanine and arginine rich domain containing protein	1		-1.88							
68074	A930013F10Rik	RIKEN cDNA A930013F10 gene	1					-1.47				
100503044	A730020M07Rik	RIKEN cDNA A730020M07 gene	1					-1.85				
269855	A430110N23Rik	RIKEN cDNA A430110N23 gene	1					-1.67				
380977	A330009N23Rik	RIKEN cDNA A330009N23 gene	1					-1.51				
223267	A2ld1	AIG2-like domain 1	1					1.54				
319469	A230056J06Rik	RIKEN cDNA A230056J06 gene	1					-2.84				
212448	9330159F19Rik	RIKEN cDNA 9330159F19 gene	1	1.49								
229550	9130204L05Rik	RIKEN cDNA 9130204L05 gene	1		-2.65							
100043133	9130023H24Rik	RIKEN cDNA 9130023H24 gene	1					1.38				
229613	6330549D23Rik	RIKEN cDNA 6330549D23 gene	1					-1.80				
70717	6330406I15Rik	RIKEN cDNA 6330406I15 gene	1					-1.49				
109198	6030407O03Rik	RIKEN cDNA 6030407O03 gene	1					-3.06				
70617	5730508B09Rik	RIKEN cDNA 5730508B09 gene	1					1.32				
66648	5730494M16Rik	RIKEN cDNA 5730494M16 gene	1		-1.91							
329735	4933431E20Rik	RIKEN cDNA 4933431E20 gene	1					-1.52				
71101	4933407H18Rik	RIKEN cDNA 4933407H18 gene	1					-1.30				
229227	4932438A13Rik	RIKEN cDNA 4932438A13 gene	1				1.54					
69034	4930579G22Rik	RIKEN cDNA 4930579G22 gene	1					-1.35				
75269	4930564D02Rik	RIKEN cDNA 4930564D02 gene	1					-1.44				
75180	4930538K18Rik	RIKEN cDNA 4930538K18 gene	1					-1.79				
320163	4930525G20Rik	RIKEN cDNA 4930525G20 gene	1					-2.25				
78185	4930524L23Rik	RIKEN cDNA 4930524L23 gene	1					-1.70				

Gene ID	Symbol	Description	DEG/Set count	2mWT_2mKO	20mWT_20mKO	2mKO_20mKO	2mWT_20mWT	2mWT_30mWT	20mWT_30mWT	Literature-Mining (Aging-Cholesterol literature)		
				9	230	406	48	1904	1157	#Paper	Enrichment	BH P-value
74704	4930518I15Rik	RIKEN cDNA 4930518I15 gene	1					-1.61				
71653	4930506M07Rik	RIKEN cDNA 4930506M07 gene	1					1.41				
66730	4921531C22Rik	RIKEN cDNA 4921531C22 gene	1					-1.44				
97820	4833439L19Rik	RIKEN cDNA 4833439L19 gene	1					-1.19				
74597	4833418N02Rik	RIKEN cDNA 4833418N02 gene	1		1.41							
320869	4732415M23Rik	RIKEN cDNA 4732415M23 gene	1					-2.01				
100040322	3830408C21Rik	RIKEN cDNA 3830408C21 gene	1					-1.41				
67290	3110040N11Rik	RIKEN cDNA 3110040N11 gene	1					1.38				
67289	3110021A11Rik	RIKEN cDNA 3110021A11 gene	1					-1.89				
100502745	2900009J06Rik	RIKEN cDNA 2900009J06 gene	1					-1.54				
70419	2810408A11Rik	RIKEN cDNA 2810408A11 gene	1					-1.69				
69953	2810025M15Rik	RIKEN cDNA 2810025M15 gene	1					-1.23				
100503178	2810013P06Rik	RIKEN cDNA 2810013P06 gene	1					-1.31				
72146	2810001A02Rik	RIKEN cDNA 2810001A02 gene	1		1.29							
72658	2700097O09Rik	RIKEN cDNA 2700097O09 gene	1					-1.39				
72657	2700094K13Rik	RIKEN cDNA 2700094K13 gene	1					1.82				
72203	2610507I01Rik	RIKEN cDNA 2610507I01 gene	1					-1.27				
72128	2610008E11Rik	RIKEN cDNA 2610008E11 gene	1					-1.27				
67513	2610002J02Rik	RIKEN cDNA 2610002J02 gene	1					-1.19				
66520	2610001J05Rik	RIKEN cDNA 2610001J05 gene	1					1.46				
76799	2510006D16Rik	RIKEN cDNA 2510006D16 gene	1					1.44				
71970	2410018M08Rik	RIKEN cDNA 2410018M08 gene	1					-1.35				
69221	2410006H16Rik	RIKEN cDNA 2410006H16 gene	1					1.39				
217732	2310044G17Rik	RIKEN cDNA 2310044G17 gene	1					1.38				
69573	2310016C08Rik	hypoxia inducible lipid droplet associated	1					1.35				
69548	2310015A10Rik	RIKEN cDNA 2310015A10 gene	1					-1.51				
71885	2310003H01Rik	RIKEN cDNA 2310003H01 gene	1					-1.40				
70134	2210011C24Rik	RIKEN cDNA 2210011C24 gene	1					-1.62				
70261	2010110P09Rik	RIKEN cDNA 2010110P09 gene	1					-1.60				
67892	1810063B05Rik	RIKEN cDNA 1810063B05 gene	1					-1.29				
75602	1810062O18Rik	RIKEN cDNA 1810062O18 gene	1					-1.69				
66291	1810030N24Rik	RIKEN cDNA 1810030N24 gene	1					-1.35				
73467	1700066M21Rik	RIKEN cDNA 1700066M21 gene	1		-1.55							
66337	1700025K23Rik	RIKEN cDNA 1700025K23 gene	1					-1.42				
66602	1700020I14Rik	RIKEN cDNA 1700020I14 gene	1		1.20							
69351	1700008A04Rik	RIKEN cDNA 1700008A04 gene	1					-1.84				
665268	1600029O15Rik	ribosomal protein L17 pseudogene	1					-1.73				
68075	1520402A15Rik	RIKEN cDNA 1520402A15 gene	1					-1.63				
78896	1500015O10Rik	RIKEN cDNA 1500015O10 gene	1					-1.96				
223776	1300018J18Rik	RIKEN cDNA 1300018J18 gene	1					-1.42				
74152	1300002K09Rik	RIKEN cDNA 1300002K09 gene	1					8.82				
66214	1190002H23Rik	RIKEN cDNA 1190002H23 gene	1					-1.53				
68497	1110018G07Rik	RIKEN cDNA 1110018G07 gene	1					1.43				
56372	1110004F10Rik	RIKEN cDNA 1110004F10 gene	1					1.36				
68550	1110002N22Rik	RIKEN cDNA 1110002N22 gene	1					1.29				
68554	1110001A16Rik	RIKEN cDNA 1110001A16 gene	1					1.38				
66096	0910001L09Rik	RIKEN cDNA 0910001L09 gene	1					1.17				
66086	0610037P05Rik	RIKEN cDNA 0610037P05 gene	1					1.29				
78687	0610025J13Rik	RIKEN cDNA 0610025J13 gene	1					-1.51				
57438	March7	membrane-associated ring finger (C3HC4) 7	1					1.54				
100503583		fibrinogen silencer-binding protein	1					-1.51				
100504348		NA	1					1.71				
